# Risk factors for pain and functional impairment in people with knee and hip osteoarthritis: a systematic review and meta-analysis

**DOI:** 10.1136/bmjopen-2020-038720

**Published:** 2020-08-07

**Authors:** Sandeep Sandhar, Toby O Smith, Kavanbir Toor, Franklyn Howe, Nidhi Sofat

**Affiliations:** 1 Institute for Infection and Immunity, University of London St George’s, London, UK; 2 Nuffield Department of Orthopaedics and Musculoskeletal Sciences, University of Oxford, Oxford, UK; 3 Molecular and Clinical Sciences Research Institute, University of London St George’s, London, UK

**Keywords:** knee, hip, pain management, rheumatology

## Abstract

**Objective:**

To identify risk factors for pain and functional deterioration in people with knee and hip osteoarthritis (OA) to form the basis of a future ‘stratification tool’ for OA development or progression.

**Design:**

Systematic review and meta-analysis.

**Methods:**

An electronic search of the literature databases, Medline, Embase, CINAHL, and Web of Science (1990–February 2020), was conducted. Studies that identified risk factors for pain and functional deterioration to knee and hip OA were included. Where data and study heterogeneity permitted, meta-analyses presenting mean difference (MD) and ORs with corresponding 95% CIs were undertaken. Where this was not possible, a narrative analysis was undertaken. The Downs & Black tool assessed methodological quality of selected studies before data extraction. Pooled analysis outcomes were assessed and reported using the Grading of Reccomendation, Assessment, Development and Evaluation (GRADE) approach.

**Results:**

82 studies (41 810 participants) were included. On meta-analysis: there was moderate quality evidence that knee OA pain was associated with factors including: Kellgren and Lawrence≥2 (MD: 2.04, 95% CI 1.48 to 2.81; p<0.01), increasing age (MD: 1.46, 95% CI 0.26 to 2.66; p=0.02) and whole-organ MRI scoring method (WORMS) knee effusion score ≥1 (OR: 1.35, 95% CI 0.99 to 1.83; p=0.05). On narrative analysis: knee OA pain was associated with factors including WORMS meniscal damage ≥1 (OR: 1.83). Predictors of joint pain in hip OA were large acetabular bone marrow lesions (BML; OR: 5.23), chronic widespread pain (OR: 5.02) and large hip BMLs (OR: 4.43).

**Conclusions:**

Our study identified risk factors for clinical pain in OA by imaging measures that can assist in predicting and stratifying people with knee/hip OA. A ‘stratification tool’ combining verified risk factors that we have identified would allow selective stratification based on pain and structural outcomes in OA.

**PROSPERO registration number:**

CRD42018117643.

**Strengths and limitations of this study**

- This study has been reported in accordance with the Preferred Reporting Items for Systematic Reviews and Meta-Analyses reporting checklist.
- Analyses have been undertaken respecting potential sources of known statistical heterogeneity.
- Searches included both published and unpublished sources of literature to reduce the risk of omitting potentially eligible data.
- There was a paucity of available data to permit meta-analyses of risk factors for pain and functional impairment.
- The variability in methods of assessing risk and reporting of frequency of risk characteristics limited analyses.

## Introduction

It has been reported that over 30.8 million US adults suffer from osteoarthritis (OA).^1^ Between 1990 and 2010, the years lived with disability worldwide caused by OA increased from 10.5 million to 17.1 million, an increase of 62.9%.^2^ Current OA treatment lacks any disease-modifying treatments with a predominance to manage symptoms rather than modify underlying disease.^3^ The clinical symptoms of OA can be assessed using several questionnaires, the most common of which is the Western Ontario and Mcmaster Universities Osteoarthritis Index (WOMAC).^4–6^ Although pain is recognised as an important outcome measure in OA, it is not clear what the optimal assessment tools are in OA and how they relate to other risk factors.

OA has various subtypes and since current therapies cannot prevent OA progression, early detection and stratification of those at risk may enable effective presymptomatic interventions.^7 8^ Several methods are used to define, diagnose and measure OA progression, including imaging techniques (eg, plain radiography, CT and MRI). Plain radiography provides high contrast and high-resolution images for cortical and trabecular bone, but not for non-ossified structures (eg, synovial fluid).^9^ The most recognised radiographic measure classifying OA severity is Kellgren and Lawrence (KL) grading which assesses osteophytes, joint space narrowing (JSN), sclerosis and bone deformity.^10 11^ However, it has been argued that MRI may be more suitable for imaging arthritic joints, providing a whole organ image of the joint.^12^ Whole-organ MRI scoring method (WORMS) is used in MRI for OA assessing damage, providing a detailed analysis of the joint.

Recently, Outcome Measures in Rheumatology-Osteoarthritis Research Society International (OMERACT-OARSI) have published a core domain set for clinical trials in hip and/or knee OA.^13^ Six domains were assessed as mandatory in the assessment of OA, including pain, physical function, quality of life, patient’s global assessment of the target joint and adverse events including mortality and/or joint structure, depending on the intervention tested. However, there remains a need to identify risk factors for pain and structural damage in OA so that potential interventions can be studied in a timely manner. The purpose of this systematic review was therefore to identify risk factors for pain, worsening function and structural damage that can predict knee/hip OA development and progression. By identifying risk factors for OA pain and structural damage, tools for stratifying specific disease groups could be developed in the future.

## Methods

This systematic review has been reported in accordance with the Preferred Reporting Items for Systematic Reviews and Meta-Analyses reporting guidelines.

### Search strategy

A systematic search of the literature was undertaken from 1 January 1990 to 1 February 2020 using electronic databases: Medline (Ovid), Embase (Ovid), Medline, Web of Science and CINAHL (EBSCO). An example of the Embase search strategy of included search terms and Boolean operators is presented in online supplementary file 1. Unpublished literature databases including Clinicaltrials.gov, the WHO International Registry of Clinical Trials and OpenGrey were also searched.

10.1136/bmjopen-2020-038720.supp1 Supplementary data

### Study identification

Studies were eligible for inclusion if they were a full-text article that satisfied all of the following:

1. One hundred or more participants analysed in the study (to increase power for comparisons).
2. Convincing definition of OA using American College of Rheumatology criteria,^14^ based on symptoms of sustained pain and stiffness in the affected joint, radiographic changes including osteophytes, cartilage loss, bone cysts/sclerosis and JSN, with normal inflammatory markers.
3. Abstract/title that must refer to pain and/or structure in relation to OA as a primary disease.
4. Knee or hip OA.
5. Pain and/or function scores.
6. Joint imaged.
7. Minimum 6-month follow-up of pain/function outcome measures.

Non-English studies, letters, conference articles and reviews were excluded.

The titles and abstracts were reviewed by one reviewer (SS). The full text for each paper was assessed for eligibility by one reviewer (SS) and double-checked by a second (TOS). Any disagreements were addressed through discussion and adjudicated by a third reviewer (NS or FH). All studies that satisfied the criteria were included in the review.

### Quality assessment

To assess the risk of bias and the power of the methodology, the Downs & Black (D&B) tool was applied.^15^ These tools assessed the following aspects of each study: reporting quality, external validity, internal validity-bias, selection bias and power. The modified D&B tool was used. Accordingly, the 27-item randomised controlled trial (RCT) version was used for RCTs while the 18-item non-RCT version was used for non-RCT designs (online supplementary file 2). Both 18-item and 27-item tools have been demonstrated to be valid and reliable tools to assess RCT and non-RCT papers.^14^ Critical appraisal was performed by one reviewer (SS) and verified by a second (KT). Any disagreements were dealt with by discussion and adjudicated through a third reviewer (TOS). In previous literature, D&B score ranges were given corresponding quality: excellent (scored 26–28); good (scored 20–25); fair (scored 15–19); and poor (scored <14).^14^ Item 4 on the non-RCT and item 5 from the RCT tool are scored two points; hence, the total scores equate to 19 and 28 points, respectively. The D&B tool was used to exclude poor quality studies with a score 15/28 or lower in RCTs and 10/19 or lower in non-RCTs.

### Data extraction

Data were extracted including: subject demographic data, study design, pain and function outcome measures, imaging used, OA severity scores, change in pain and function outcomes and change in OA severity scores. After all relevant data had been extracted, authors of these papers were approached to try and attain individual patient data related to baseline and change in pain, function and structural scores for each study. No data were received from authors to inform this analysis.

### Outcomes

The primary outcome was to determine the development of pain and functional impairment for those with knee and hip OA. The secondary outcome was to determine which factors are associated with structural changes in knee and hip OA.

### Data analysis

All data were assessed for study heterogeneity through scrutiny of the data extraction tables. These identified that there was minimum study-based heterogeneity based on: population, study design and interventions-exposure variabilities for given outcomes. Where there was study heterogeneity, a narrative analysis was undertaken. In this instance, the ORs of all predictor variables were tabulated with a range of OR presented. Where there was sufficient data to pool (two or more studies with data available to analyse) and study homogeneity evident, a pooled meta-analysis was deemed appropriate. As interpreted by the Cochrane Collaboration,^16^ when I^2^ was 50% or greater representing high-statistical heterogeneity, a random-effect model meta-analysis was undertaken. When I^2^ was less than this figure, a fixed effects model approach was adopted. Continuous outcomes were assessed using mean difference (MD) scores of measures for developing severe OA, whereas dichotomous variables were assessed through OR data. All data were presented with 95% CIs and forest plots.

Due to the presentation of the data, there were minimal data to permit meta-analyses. Where there were insufficient data to pool the analysis (data only available from one study), a narrative analysis was undertaken to assess risk factors for the development of increased pain and functional impairment. Planned subgroup analyses included determine whether there was a difference in risk factors based on: (1) anatomical regions (ie, difference between hip OA and knee OA); (2) geographical region. Analyses were undertaken on STATA V.14.0 (Stata Corp) with forest plots constructed using RevMan Review Manager (RevMan; Computer program; V.5.3. Copenhagen: The Nordic Cochrane Centre, The Cochrane Collaboration, 2014.)

### Patient and public involvement

The research team acknowledges the assistance of both the OA tech network and Engineering and Physical Sciences Research Council. The authors also acknowledge receiving assistance from a meeting that enabled a consensus to be met on the eligibility criteria to be used, and this meeting consisted of the following people: Angela Kedgley, Abiola Harrison, Alan Boyde, Alan Silman, Amara Ezeonyeji, Caroline Hing, Cathy Holt, Debbie Rolfe, Enrica Papi, Freija Ter Heegde, Jingsong Wang, John Garcia, Mark Elliott, Mary Sheppard, Natasha Kapella, Richard Rendle, Shafaq Sikandar, Sherif Hosny, Soraia Silva, Soraya Koushesh, Susanna Cooper and Thomas Barrick. No writing assistance was used.

## Results

### Search strategy

**Figure 1:**
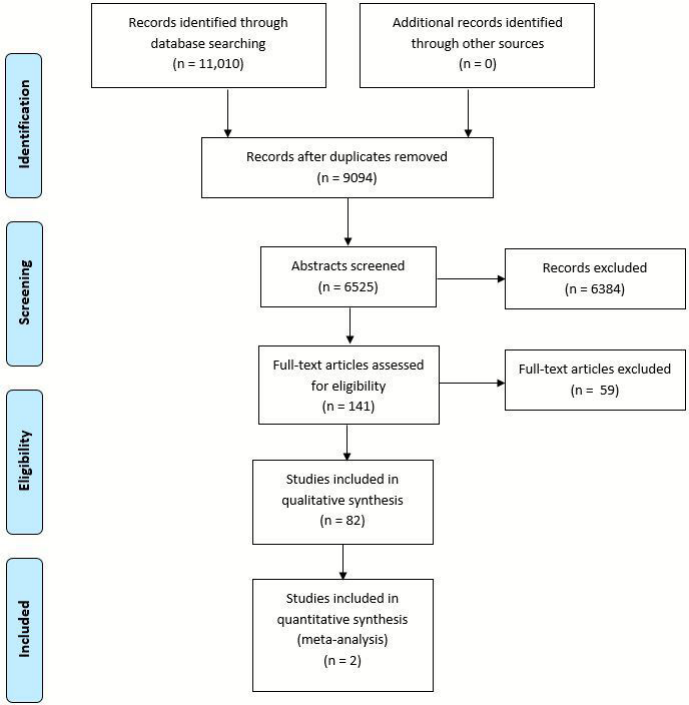
Preferred Reporting Items for Systematic Reviews and Meta-Analyses flow chart.

The results of the search strategy are presented in figure 1. In total, 11 010 citations were identified. Of these, 141 papers were deemed potentially eligible and screened at full-text level. Of these, 82 met the selected criteria and were included.^17–98^

### Characteristics of included studies

A summary of the included studies is presented as table 1. This consisted of 31 non-RCTs (27 observational cohort studies/four case-control studies) and 51 RCTs.

**Table 1:** Characteristics of included studies

	Study design	Number joints (hip/knees)	Gender (male:female)	Country origin	Mean age (years)	Follow-up duration (months)	Pain outcome measures	Functional outcome measures
Ahedi *et al* 54	Observational cohort	198 hips	111:87	Australia	UTD	132	WOMAC Pain	NA
Akelman *et al* 20	RCT	107 knee	UTD	USA	23.5	84	KOOS pain; SF-36 Body pain	SF-36 Physical; AP laxity; IKDC2000
Amin *et al* 55	Observational cohort	265 knees	152:113	USA	67	30	VAS Pain	WOMAC Function
Antony *et al* 56	Observational cohort	463 knees	245:218	USA	63	24	WOMAC Pain	NA
Arden *et al* 57	RCT	474 knees	185:289	UK	64	36	WOMAC Pain	WOMAC Function
Ayral *et al* 58	RCT	665 knees	259:406	Australia, Belgium, Canada, Denmark, Finland, France, Hungary, Norway, Spain, UK, USA	61.3	12	WOMAC Pain	WOMAC Function
Baselga Garcia-Escudero and Miguel Hernández Trillos^59^	Observational cohort	118 knees	43:75	Spain	59.1	24	NRS; WOMAC Pain	WOMAC Function
Bevers *et al* 60	Observational cohort	125 knees	57:68	The Netherlands	57	24	WOMAC Pain	WOMAC Function
Bingham *et al* 53	RCT	2483 knees	735:1748	USA Canada Austria Czech Republic France Germany Hungary Ireland Italy The Netherlands Poland Croatia	UTD	24	WOMAC Pain	WOMAC Function
Birmingham *et al* 61	Observational cohort	126 knees	100:26	Canada	47.5	24	KOOS Pain	KOOS Function; SF-36 Physical; LEFS
Bisicchia *et al* 52	RCT	150 knees	47:103	Italy	UTD	12	VAS Pain; SF-36	SF-36
Brandt *et al* 62	RCT	431 knees	0:431	USA	54.9	30	WOMAC Pain; VAS Pain	WOMAC Function
Brown *et al* 51	RCT	690 knees	270:420	USA	UTD	32 weeks	WOMAC Pain; NRS weekly pain	WOMAC Function; SF-36 Function
Brown *et al* 50	RCT	621 hips	237:384	USA	UTD	32 weeks	WOMAC Pain	WOMAC Function
Bruyere *et al* 63	RCT	319 knee	0:319	Belgium	64.0	36	WOMAC Pain	WOMAC Function
Campbell *et al* 49	RCT	100 knees	28:72	Australia	UTD	120	American Knee Society Score; WOMAC Pain	American Knee Society Score (function); WOMAC Function
Chandrasekaran *et al* 48	Case control	111 hips	66:45	USA	UTD	24	Modified Harris Hip Score; Nonarthritic hip score; VAS Pin	Modified Harris Hip Score; Nonarthritic hip score; Hip Outcome Score; Sports & ADLs
Chandrasekaran *et al* 47	Case control	186 hips	96:90	USA	UTD	24	Modified Harris Hip Score; Nonarthritic hip score; VAS Pin	Modified Harris Hip Score; Nonarthritic hip score; Hip Outcome Score; Sports & ADLs
Conrozier *et al* 64	RCT	205 knees	88:117	France	65	26	WOMAC Pain; NRS walking pain	WOMAC Function
Davis *et al* 19	Case control	3132 knees	UTD	USA	UTD	48	WOMAC Pain; KOOS Pain	WOMAC Function
Dougados *et al* 46	RCT	507 hips	202:305	France	UTD	36	VAS Pain	Lequesne Index
Dowsey *et al* 65	Observational cohort	478 knees	147:331	Australia	70.8	24	IKSS Pain	IKSS Function
Eckstein *et al* 45	RCT	1412 knees	611:801	Austria	UTD	48	WOMAC Pain	NA
Ettinger *et al* 44	RCT	439 knees	131:308	USA	UTD	18	Pain intensity score	Physical Test
Felson *et al* 66	Observational cohort	3498 knees	867:1206	USA	61.2	30	WOMAC Pain	PASE
Felson *et al* 67	Observational cohort	330 knees	111:2111	USA	62.1	15	NA	Quadriceps strength (N)
Filardo *et al* 43	RCT	183 knees	112:71	Italy	UTD	48	KOOS Pain; IKDC	KOOS Function; Tegner; IKDC
Glass *et al* 42	Observational cohort	4648 knees	918:1486	USA	UTD	24	WOMAC Pain; NRS Pain	WOMAC Function
Guermazi *et al* 41	Case control	493 knees	185:308	USA	UTD	60	WOMAC Pain	PASE
Hamilton *et al* 68	Observational cohort	805 knees	416:289	UK	66	30	WOMAC Pain	WOMAC Function
Hellio le Graverand *et al* 69	RCT	1457 knees	343:1114	USA Canada Australia, Belgium, Czech Republic, Germany, Hungary, Italy, Poland, Russian Federation, Slovakia, Spain, Argentina Peru	61.0	180	Oxford Knee Score	Oxford Knee Score; American Knee Society Score; Tegner
Henriksen *et al* 40	RCT	157 knees	28:129	Denmark	UTD	24	WOMAC Pain	WOMAC Function
Hill *et al* 5	RCT	202 knees	102:100	Australia	61	12	KOO Pain	KOOS Function and kinematic assessment
Hochberg *et al* 70	RCT	522 knees	84:438	France Germany Poland Spain	62.7	24	WOMAC Pain	WOMAC Function
Hoeksma *et al* 71	RCT	109 hips	33:76	The Netherlands	72	6	WOMAC Pain; Huskisson’s VAS; EQ-5D Pain	WOMAC Function; EQ-5D Function
Housman *et al* 39	RCT	391 knees	130:261	USA Canada France UK Germany	UTD	6	SF-36 Body Pain; Harris Hip Score; VAS Pain	SF-36 Function; Harris Hip Score; ROM
Huang *et al* 72	RCT	264 knees	39:93	Taiwan	62	6	WOMAC Pain	NA
Huizinga *et al* 73	Observational cohort	298 knees	201:97	The Netherlands	51	12	VAS Pain	Lequesne index; walking speed
Jin *et al* 6	RCT	413 knees	205:208	Australia	63.2	24	WOMAC Pain; VAS Pain	WOMAC Function
Kahn *et al* 74	Observational cohort	174 knees	70:102	USA	67.0	6	WOMAC Pain	WOMAC Function
Karsdal *et al* 38	RCT	2207 knees	773:1424	Denmark	UTD	24	WOMAC Pain	WOMAC Function
Katz *et al* 37	RCT	330 knees	143:187	USA	UTD	12	KOO Pain	WOMAC Function; SF-36 Function
Kim *et al* 75	RCT	352 knees	9:153	Republic of Korea	68.1	144	WOMAC	Knee Society Knee Score Function; ROM; UCLA Activity
Kinds *et al* 18	RCT	565 knees	UTD	The Netherlands	UTD	60	WOMAC Pain	WOMAC Function
Kongtharvonskul *et al* 36	RCT	148 knees	25:123	Thailand	UTD	6	WOMAC Pain; VAS Pain	WOMAC Function
Lequesne *et al* 76	RCT	163 hips	102:61	France	63.2	24	VAS Pain	Lequesne Index
Lohmander *et al* 35	RCT	170 knees	52:116	Bulgaria Canada Croatia Finland Germany Poland Serbia Africa Sweden USA	UTD	12	WOMAC Pain	WOMAC Function
Maheu *et al* 8	RCT	345 hips	159:186	France	62.2	36	WOMAC Pain; Global Hip Pain	Lequesne Index; WOMAC Function; Global handicap NRS
Marsh *et al* 34	RCT	168 knees	57:112	Canada	UTD	24	WOMAC	WOMAC
McAlindion *et al* 33	RCT	146 knees	57:89	USA	UTD	24	WOMAC Pain	WOMAC Function; Physical Test
Messier *et al* 32	RCT	316 knees	89:227	USA	UTD	18	WOMAC Pain	WOMAC Function; Physical Test
Messier *et al* 77	RCT	142 knees	37:105	USA	68.5	18	WOMAC Pain	WOMAC Function; Physical Test
Messier *et al* 78	RCT	454 knees	128:325	USA	66	18	WOMAC Pain	WOMAC Function; Physical Test; SF-36 Physical
Michel *et al* 31	RCT	300 knees	146:154	Switzerland	UTD	24	WOMAC Pain	WOMAC Function; Physical Test
Muraki *et al* 79	Observational cohort	1558 knees	553:1005	Japan	67.0	40	WOMAC Pain	WOMAC Function;
Muraki *et al* 80	Observational cohort	1525 knees	546:979	Japan	67.0	40	WOMAC Pain	WOMAC Function
Pavelka *et al* 30	RCT	277 knees; 117 hips	109:285	Czech Republic	58	60	NA	Lequesne Index
Pavelka *et al* 81	RCT	202 knees	45:157	Czech Republic	UTD	36	WOMAC Pain	WOMAC Function; Lequesne Index
Pham *et al* 29	Observational cohort	301 knees	97:204	France	UTD	12	VAS Pain	Lequesne Index
Podsiadlo *et al* 28	Observational cohort	114 knees	49:65	Australia	UTD	72	WOMAC Pain	WOMAC Function
Rat *et al* 82	RCT	300 knees	118:182	France	67	6	SF-36 Body Pain; OAKHQOL Pain; VAS Pain	Lequense Index; SF-36 Physical; OAKHQOL Physical Activity
Raynauld *et al* 27	RCT	123 knees	44:79	Canada	UTD	24	WOMAC Pain	WOMAC Function
Reginster *et al* 26	RCT	212 knees	50:162	Belgium	UTD	36	WOMAC Pain	WOMAC Function
Reginster *et al* 83	RCT	1371 knees	425:946	Australia Austria Belgium Canada Czech Republic Denmark Estonia France Germany Italy Lithuania The Netherlands Poland Portugal Romania Russian Federation Spain UK	62.9	36	WOMAC Pain; VAS Pain	WOMAC Function
Riddle and Jiranek^25^	Observational cohort	467 knees	209:258	USA	UTD	24	KOOS Pain	WOMAC Function
Romagnoli *et al* 84	Observational cohort	105 knees	16:69	Italy	67.7	66	Knee Society Score Clinical; VAS Pain	Knee Society Score Function; ROM
Roman-Blas *et al* 24	RCT	158 knees	26:132	Spain	UTD	6	WOMAC Pain; VAS Pain	WOMAC Function
Rozendaal *et al* 31	RCT	222 hips	68:154	The Netherlands	UTD	24	WOMAC Pain; VAS Pain	WOMAC Function
Sanchez-Ramirez *et al* 85	Observational cohort	186 knees	59:127	Canada	61	24	WOAMC Pain	WOMAC Function; Physical Test
Sawitzke *et al* 86	RCT	662 knees	215:447	USA	57	24	WOMAC Pain	WOMAC Function
Skou *et al* 87	Observational cohort	1682 knees	434:818	Denmark	62.2	84	WOMAC Pain	PASE; Physical Test
Sowers *et al* 88	Observational cohort	724 knees	0:363	USA	56	132	NA	WOMAC Function; Physical Test
Spector *et al* 89	RCT	284 knees	115:169	UK	63.3	12	WOMAC Pain	WOMAC Function
Sun *et al* 90	RCT	121 knees	31:90	Taiwan	63	6	WOMAC Pain; VAS Pain	WOMAC Function; Lequesne Index; Physical Test
Urish *et al* 22	RCT	336 knees	96:67	USA	UTD	36	WOMAC	WOMAC
Valdes *et al* 17	Observational cohort	860 knees; 928 hips	UTD	UK	UTD	38	WOMAC Pain	NA
Van der Esch *et al* 98	Observational cohort	402 knees	64:137	The Netherlands	61.2	24	NRS Pain	WOMAC Function; Physical Test
Weng *et al* 91	RCT	264 knees	26:106	Taiwan	64	12	VAS Pain	Lequesne Index; ROM; Physical Test
White *et al* 92	Observational cohort	2110 knees	992:118	USA	61.0	84	VAS Pain	WOMAC Function
Witt *et al* 93	RCT	294 knees	70:154	Germany	64.0	12	WOMAC Pain; SF-36 Body Pain; VAS Pain	WOMAC Function; SF-36 Function
Yu *et al* 21	Observational cohort	204 knees	74:130	Australia	UTD	12	KOOS Pain; VAS Pain	KOOS ADL; Physical Function
Yusuf *et al* 94	Observational cohort	74 knees; 31 hips; 11 hip and knees	19:98	The Netherlands	60	72	WOMAC Pain; SF-36 Body Pain; Pain on movement	WOMAC Function; SF-36 Function; Physical Test

ADLs, activities of daily living; IKDC, International Knee Documentation Committee; KOOS, Knee Injury and Osteoarthritis Outcome Score; LEFS, Lower Extremity Functional Scale; NA, not applicable; NRS, Numerical Rating Scale; OAKHQOL, Osteoarthritis Knee and Hip quality of Life Questionnaire; PASE, Physical Activity Scale for the Elderly; RCT, randomised controlled trial; ROM, range of motion; SF-36, Short Form-36; UTD, unable to determine; VAS, Visual Analogue Scale; WOMAC, Western Ontario and Mcmaster Universities Osteoarthritis Index.

In total, 45 767 knees were included in the analysis. This consisted of 13 870 men and 23 497 women; 4 studies did not report the gender of their cohorts.^17–20^ Thirty-six studies were undertaken in the USA; 30 were undertaken in Europe; 9 were conducted in Australasia and 7 in Asia. Mean age of the cohorts was 61.7 years (SD: 7.56); 36 studies did not report age.^17 21–54^ Mean follow-up period was 35.4 months (SD: 33.6). The most common measures of pain were WOMAC pain (n=55; 50%) and Visual Analogue Scale (VAS) Pain (n=21; 19%). The most frequently used measures of function were WOMAC function (n=52; 44%), physical tests (n=16; 14%) and SF-36 (n=10; 9%).

### Methodological quality assessment

The methodological quality of the evidence was moderate (online supplementary file 2;. Based on the results of the D&B non-RCT tool (31 studies; online supplementary file 2), recurrent strengths of the evidence were clear description of the participants recruited (29 studies; 94%), the representative nature that participants were to the population (31 studies; 100%), and variability in data presented for the main outcomes (31 studies; 100%). Furthermore, the main outcome measures were deemed reliable and valid in all studies (31 studies; 100%) with 89% (27 studies; 87%) studies adopting appropriate statistical analyses for their datasets. Recurrent limitations were not clearly reporting the main findings (20 studies; 65%), issues regarding the representation of the cohort from the wider public (18 studies; 58%) and only 6 studies (19%) basing their sample sizes on an *a prior* power calculation.

The results from the D&B RCT checklist (51 studies; online supplementary file 3) similarly reported findings with strength of the evidence around clear reporting of the cohort characteristics (49 studies; 96%) and interventions (50 studies; 98%), adoption of reliable/valid outcome measures (51 studies; 100%) and reported high compliance to study processes (37 studies; 73%). Recurrent weaknesses included recruiting cohorts which may not have been reflective of the wider population (19 studies; 37%), in clinic settings which may not have represented typical clinical practice (21 studies; 41%) and poorly adjusting for potential confounders in analyses (26 studies; 51%).

### Knee OA

#### Narrative review

Findings from the narrative analysis found the following were predictors for worsening joint pain: KL3 or 4 in women (OR: 11.3; 95% CI 6.2 to 20.4), a WORMS lateral meniscal cyst (MC) score of 1 (OR: 4.3; 95% CI 1.2 to 15.4), presence of chronic widespread pain (CWP; OR: 3.2; 95% CI 1.9 to 5.3), increase of ≥2 in WORMS BML score after 15 months (OR: 3.2; 95% CI 1.5 to 6.8), meniscal maceration (OR: 2.8; 95% CI 1.8 to 4.4) or damage ≥2 in WORMS (OR: 1.8; 95% CI 0.9 to 3.6). We also found that the following were the highest predictors of worsening function in people with knee OA: KL of <3 (OR: 3.3; 95% CI 0.7 to 15.9), modified KL 3a (OR: 1.7; 95% CI 0.7 to 3.8), modified KL 4a (OR: 1.5; 95% CI 0.7 to 3.0), presence of osteophytes (OR: 1.3; 95% CI 0.7 to 2.4), female gender (OR: 1.8 (95% CI 1.1 to 3.0) to OR: 2.1 (95% CI 1.2 to 3.5)), ethnicity (OR: 1.03; 95% CI 0.59 to 1.83) and synovitis ≥1 (OR: 1.3; 95% CI 0.8 to 1.9).

#### Meta-analysis

**Figure 2:**
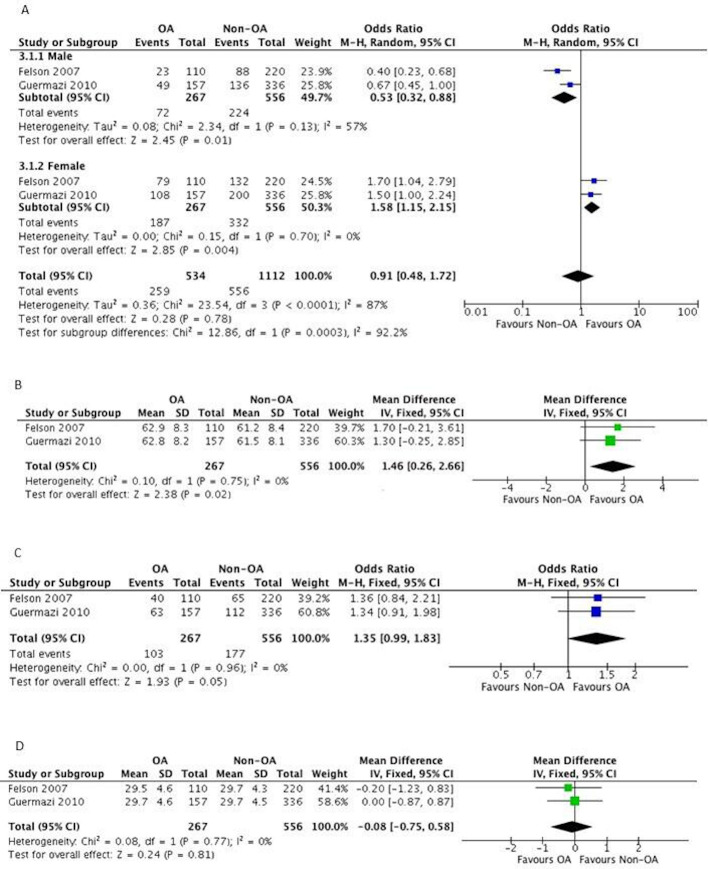
(A) Forest plot to present the association between gender and presentation of knee osteoarthritis (OA). (B) Forest plot to present the association between age and presentation of knee OA. (C) Forest plot to present the association between knee effusion score greater or equal to 1 and presentation of knee OA. (D) Forest plot to present the association between body mass index and presentation of knee OA.

Two studies were identified where data could be evaluated for OA risk factors by meta-analysis.^41 67^ Three variables significantly associated with the development of knee OA. As illustrated in table 2 and figure 2A–D, age (MD: 1.46, 95% CI 0.26 to 2.66; p=0.02; n=823), KL of ≥2 (MD: 2.04, 95% CI 1.48 to 2.81; p<0.01; n=823) and knee effusion score ≥1 (OR: 1.35, 95% CI 0.99 to 1.83; p=0.05; n=823) were all associated with the development of knee OA based on moderate quality evidence. The variables of gender and BMI were not shown to be significantly associated with the knee OA development (table 2).

**Table 2:** Meta-analysis results: exhibit knee osteoarthritis

Variable	N	Effect estimate	P value	Statistical heterogeneity (I^2^ %)	GRADE assessment
Gender	823	0.91 (0.48 to 1.72)*	0.78	87	Low-quality evidence†
Age	823	1.46 (0.26 to 2.66)	0.02	0	Moderate-quality evidence‡
KL ≥2	823	2.04 (1.48 to 2.81)	<0.01	35	Moderate-quality evidence‡
Knee effusion score ≥1	823	1.35 (0.99 to 1.83)	0.05	0	Moderate-quality evidence‡
BMI	823	−0.08 (−0.75 to 0.58)	0.81	0	Moderate-quality evidence‡

*Random effects model analysis.

†GRADE—outcomes downgraded one level due to risk of bias, two level due to imprecision and inconsistency.

‡GRADE—outcomes downgraded one level due to risk of bias.

BMI, body mass index; I2, inconsistency squared; KL, Kellgren Lawrence Scale; N, number of participants in analysis; NE, not estimable.

Due to the limited availability of data, it was not possible to conduct the planned subgroup analyses to determine whether there was a difference in risk factors based on anatomical or geographical regions.

### Hip OA

#### Narrative analysis

This was based on low-quality evidence. There was no association between the development of hip BML and BMI or age. Predictors for worsening joint pain for people with hip OA included a large acetabular BML (OR: 5.2; 95% CI 1.2 to 22.9), a large femoral head BML (OR: 4.4; 95% CI 1.4 to 19.7) with any large hip BML (OR: 4.4; 95% CI 1.5 to 13.2), CWP (OR: 5.0; 95% CI 2.8 to 9.1) and depression (OR: 1.9; 95% CI 1.2 to 2.9). Baseline knee pain score (MD:−1.4; 95% CI −1.6 to −1.2) and baseline hip pain score (MD:−0.7; 95% CI −1.0 to −0.5) were significantly associated with the development of hip BMLs and pain.

#### Meta-analysis

There were insufficient data to permit meta-analysis for the hip OA dataset.

## Discussion

Our systematic review and meta-analysis identified risk factors for knee and hip OA pain and structural damage based on evaluation of 82 studies. For the knee, increasing pain in knee OA was associated with KL grade 3 or 4 in women, WORMS lateral MC, presence of CWP, increase of ≥2 in WORMS BML score after 15 months and meniscal maceration. In addition, KL <3, KL 3a, KL 4a, osteophyte presence and female gender were associated with worsening function in people with knee OA. On meta-analysis, age, radiological features (KL score of 2 or more) and knee effusion were associated with development and/or progression of knee OA.

Our meta-analysis identified risk factors that are appreciated only when results were pooled together. These were namely WORMS-defined knee effusion score ≥1. To our knowledge, this is currently the largest and most up to date systematic review of its kind, reviewing 82 primary studies in 41 810 participants. Nonetheless, some risk factors from our meta-analysis have been recognised previously. For example, Silverwood *et al* reported previous injuries are associated to developing knee OA, supporting the present analysis.^95^ Kingsbury *et al* identified age and KL grade as predictive factors for developing knee OA, supporting the present findings.^96^ The meta-analyses provided both novel and supporting findings for risk factors associated with developing and progressing knee OA. A machine learning study assessed risk factors associated with pain and radiological progression in knee OA found that BMLs, osteophytes, medial meniscal extrusion, female gender and urine CTX-II contributed to progression.^97^ Nelson *et al’s* work is supported by other studies.^95 96^ Therefore, the findings of our analysis support previous findings.

After plain radiography, MRI was the most used modality with WORMS as the most common scoring reported for MRI. The MRI Osteoarthritis Knee Score (MOAKS),^99^ expanded on WORMS by scoring entire subregions for BMLs rather than each BML, further division of cartilage regions and refined the features assessed in meniscal morphology. Due to this progression from WORMS, having no MOAKS studies included in our final selection was surprising. This could be due to the eligibility criteria being too restrictive. A future systematic review and meta-analysis focusing on the imaging aspect of evaluating OA will be important. In hip OA, the evaluation of BML size and location is essential in predicting pain progression and these can be assessed effectively using MRI. We recommend that all MRI studies for hip OA evaluate BML size and location.

Gait analysis is considered a risk factor for pain/function and was therefore included as a target outcome measure. However, few studies included gait analysis measures, which could not be included in the analysis, perhaps due to the minimum sample size (n=100) being too restrictive.

There were several limitations within our study. First, despite identifying novel risk factors for exhibiting knee OA, a small dataset was pooled together for the meta-analysis (two studies) compared with Silverwood *et al* (34 studies).^93^ This was particularly apparent for hip OA where only 12 studies assessed this population.^8 17 23 30 46–48 50 54 71 76 94^ Consequently, the small dataset influenced the GRADE assessment that determined the evidence as low to moderate, restricting the strength of the associations of risk factors with OA development and progression. Further work may impact our confidence in the estimated effect, for both studies recruiting participants with hip and knee OA. Second, the eligibility criteria may have been too restrictive, resulting in limited papers including gait analysis or MOAKS. Wet biomarkers were not included in our analyses. Finally, the inability to pool data was partly attributed to variability in methods to report data. Standardising data collection and reporting are important in conducting meta-analyses. We believe the following should be undertaken to improve data pooling in future work: ensuring group comparisons in studies are selected from the same population (people with confirmed OA) to improve internal validity, observational studies should conduct a power analysis to determine sample sizes and all studies should include absolute frequency of events data rather than summary ORs. Such considerations will improve future meta-analyses to identify OA risk factors.

To conclude, our work helps to develop steps towards building a stratification tool for risk factors for knee OA pain and structural damage development. We also highlight the need for collection of core datasets based on defined domains, which has recently also been highlighted by the OMERACT-OARSI core domain set for knee and hip OA.^13^ Collection of future datasets based on standardised core outcomes will assist in more robust identification of risk factors for large joint OA.

## Supplementary Material

Reviewer comments

Author's manuscript
