# Supplementary material for: Risk factors for pain and functional impairment in people with knee and hip osteoarthritis: a systematic review and meta-analysis
Source: BMJ Open. 2020 Aug 7;10(8):e038720. doi: 10.1136/bmjopen-2020-038720 (PMC7418691; doi:10.1136/bmjopen-2020-038720)
Supplement: Supplementary data [file bmjopen-2020-038720supp001.pdf]

Supplementary File 1: Search strategy adopted for the EMBASE database search.

1 exp arthritis/
2 exp osteoarthritis/
3 exp joint diseases/
4 exp arthropathy/
5 exp arthralgia/
6 exp joint pain/
7 exp chronic pain/
8 exp gonarthrosis/
9 exp osteoarthrosis/
10 exp degenerative arthritis/
11 (degenerative adj2 arthritis).tw.
12 or/1-11
13 (Hip adj2 Joint).mp.
14 (Knee adj2 Joint).mp.
15 or/13-14
16 and/12,15
17 Imaging.tw.
18 Radiography.tw.
19 Radiology.tw.
20 Magnetic resonance imagining.tw.
21 MRI.tw.
22 Computed Tomography.tw.
23 CT.tw.
24 Ultrasound.tw.
25 US.tw.
26 USS.tw.
27 Sonography.tw.
28 X-ray.tw.
29 Radiograph.tw.
30 PET.tw.
31 Bone marrow lesions.tw.
32 BML.tw.
33 cytokines.tw.
34 extracellular matrix degradation.tw.
35 ECM degradation.tw.
36 GWAS.tw.
37 cartilage.tw.
38 serum.tw.
39 synovitis.tw.
40 or/17-39
41 exp pain/
42 exp peripheral nervous system disease/
43 exp somatosensory disorders/
44 ((pain* or discomfort*) adj10 (central or complex or nerv* or neuralg* or neuropath*)).mp.
45 ((neur* or nerv*) adj6 (compress* or damag*)).mp.
46 WOMAC.tw.
47 McGill.tw.
48 VAS.tw.
49 Visual analogue.tw.
50 NRS.tw.
51 Numerical rating scale.tw.
52 Analgesic.tw.
53 Analgesia.tw.
54 or/41-53
55 or/40,54
56 and/16,55
57 limit 56 to English language

Supplementary File 2: Methodological appraisal results based on the Downs and Black non-RCT Checklist

	Downs and Black Non-Randomised Controlled Trial Checklist Items																		
	**1**	**2**	**3**	**4**	**5**	**6**	**7**	**8**	**9**	**10**	**11**	**12**	**13**	**14**	**15**	**16**	**17**	**18**	**Total**
Ahedi (54)	1	1	1	0	1	1	0	1	1	1	1	UC	1	1	1	UC	1	0	13
Amin (55)	1	1	0	1	1	1	1	0	0	UC	1	1	1	1	UC	1	1	0	12
Antony (560	1	1	1	2	1	1	1	0	1	0	UC	UC	1	1	1	UC	1	0	13
Baselga Garcia-Escudero (59)	1	1	1	0	1	1	1	1	UC	UC	1	1	1	1	UC	0	1	1	13
Bevers (60)	1	1	1	2	0	1	1	1	UC	0	1	1	1	1	1	1	1	0	15
Birmingham (61)	1	1	1	1	1	1	1	1	1	1	UC	1	1	UC	1	1	1	0	15
Chandrasekaran (48)	1	1	1	1	1	1	1	1	0	UC	1	1	1	1	UC	1	1	1	15
Chandrasekaran (47)	1	1	1	1	1	1	0	1	0	UC	1	1	1	1	UC	1	UC	1	13
Davis (19)	1	1	1	0	0	1	1	0	1	1	1	UC	1	1	1	UC	1	0	12
Dowsey (65)	1	1	1	1	1	1	1	1	1	1	1	UC	1	1	1	1	1	0	16
Felson (66)	1	1	1	1	0	1	1	1	0	UC	1	1	1	UC	1	1	1	0	13
Felson (67)	1	1	1	1	0	1	1	1	0	UC	1	1	1	UC	1	1	1	0	13
Glass (42)	1	1	1	2	0	1	1	1	1	1	1	1	1	1	1	1	1	0	17
Guermazin (41)	1	1	1	2	0	1	1	1	1	1	1	1	1	UC	1	1	1	0	16
Hamilton (68)	1	1	0	0	1	1	1	1	UC	UC	1	1	1	1	UC	UC	1	1	12
Huizinga (73)	1	1	1	0	1	1	1	0	UC	UC	1	1	1	1	UC	0	1	0	11
Khan (74)	1	1	1	1	0	1	1	1	1	1	0	1	1	UC	1	1	1	0	14
Muraki (79)	1	1	1	1	1	1	1	1	1	UC	1	1	1	1	0	1	1	0	15
Muraki (80)	1	1	1	2	1	1	1	1	1	UC	1	1	1	1	0	1	1	0	16
Pham (29)	1	1	1	1	1	1	1	1	UC	UC	1	1	1	0	1	1	1	0	14
Podsiadlo (28)	1	1	1	1	0	1	1	1	UC	UC	1	1	1	UC	UC	1	1	0	12
Riddle (25)	1	1	1	2	1	1	0	0	1	1	0	1	1	1	1	1	1	1	16
Romagnoli (84)	1	0	1	0	0	1	1	1	1	1	1	1	1	UC	1	UC	1	1	13
Sanchez-Ramirez (85)	1	1	1	2	1	1	1	1	1	1	1	1	1	UC	1	1	1	0	17
Skou (87)	1	1	1	2	0	1	1	1	1	1	1	1	1	UC	1	1	1	0	16
Sowers (88)	1	1	1	0	1	1	1	1	1	UC	1	1	1	UC	1	0	0	0	12
Valder (17)	1	1	1	1	1	1	0	1	UC	UC	1	1	1	0	1	1	0	0	12
Van der Esch (99)	1	1	1	1	1	1	1	1	1	1	1	1	1	UC	1	0	1	0	15
White (92)	1	1	1	2	0	1	1	0	1	1	1	1	1	1	0	1	1	0	15
Yu (21)	1	1	1	1	1	1	1	1	1	1	1	1	1	1	1	1	1	0	17
Yusuf (94)	1	1	1	1	1	1	1	0	UC	UC	1	1	1	UC	1	1	1	0	13

UC: Unclear; 2: Yes; 1: Yes/partially; 0: No

Checklist items

1. Is the hypothesis/aim/objective of the study clearly described?
2. Are the main outcomes to be measured clearly described in the Introduction or Methods section?
3. Are the characteristics of the patients included in the study clearly described?
4. Are the distributions of principal confounders in each group of subjects to be compared clearly described?
5. Are the main findings of the study clearly described?
6. Does the study provide estimates of the random variability in the data for the main outcomes?

Sandhar S, *et al. BMJ Open* 2020; 10:e038720. doi: 10.1136/bmjopen-2020-038720

7. Have the characteristics of patients lost to follow-up been described?
8. Have actual probability values been reported (e.g. 0.035 rather than <0.05) for the main outcomes except where the probability value is less than 0.001?
9. Were the subjects asked to participate in the study representative of the entire population from which they were recruited?
10. Were those subjects who were prepared to participate representative of the entire population from which they were recruited?
11. If any of the results of the study were based on “data dredging”, was this made clear?
12. Were the statistical tests used to assess the main outcomes appropriate?
13. Were the main outcome measures used accurate (valid and reliable)?
14. Were study participants in different groups (trials and cohort studies) or were the cases and controls (case-control studies) recruited over the same period of time?
15. Were the participants in different groups (trials and cohort studies) or were the cases and controls (case-control studies) recruited from the same population?
16. Was there adequate adjustment for confounding in the analyses from which the main findings were drawn?
17. Were losses of patients to follow-up taken into account?
18. Did the study mention having conducted a power analysis to determine the sample size needed to detect a significant difference in effect size for one or more outcome measures?

Supplementary File 3: Methodological appraisal results based on the Downs and Black RCT Studies Checklist

	**Downs and Black Randomised Controlled Trial Checklist Items**																											
	**1**	**2**	**3**	**4**	**5**	**6**	**7**	**8**	**9**	**10**	**11**	**12**	**13**	**14**	**15**	**16**	**17**	**18**	**19**	**20**	**21**	**22**	**23**	**24**	**25**	**26**	**27**	**Total**
Akelman (20)	1	1	1	1	2	1	1	1	1	1	1	1	1	1	1	1	1	1	1	1	0	1	1	1	UC	1	1	**26**
Arden (57)	1	1	1	1	1	1	1	1	1	0	1	1	1	1	1	1	UC	1	1	1	1	1	1	1	0	1	UC	**23**
Ayral (58)	1	1	1	1	1	1	1	1	1	1	0	UC	UC	1	1	1	1	1	1	1	0	1	1	UC	UC	1	0	**20**
Bingham (53)	1	1	1	1	1	1	1	1	1	1	1	1	1	1	UC	1	1	1	1	1	0	1	0	UC	1	1	0	**22**
Bisicchia (52)	1	0	1	1	0	1	1	1	1	1	1	1	1	0	1	UC	1	1	1	1	1	1	1	0	0	1	0	**20**
Brandt (62)	1	1	1	1	1	0	1	1	1	1	UC	UC	UC	UC	1	1	1	1	1	1	UC	1	1	UC	0	1	0	**18**
Brown (50)	1	1	1	1	1	1	1	1	1	0	UC	UC	UC	1	1	1	1	1	1	1	UC	UC	1	UC	UC	UC	1	**18**
Brown (51)	1	1	1	1	1	1	1	1	1	0	UC	UC	UC	1	1	1	1	1	1	1	UC	UC	1	1	UC	1	1	**19**
Bruyere (63)	1	1	1	1	1	0	1	0	1	1	UC	UC	1	1	1	1	1	1	UC	1	UC	UC	1	UC	UC	1	1	**18**
Campbell (49)	1	1	1	1	0	0	0	1	1	0	1	UC	1	1	1	1	1	1	1	1	1	1	1	1	UC	1	0	**20**
Conrozier (64)	1	1	1	1	0	0	1	1	1	1	UC	UC	UC	1	1	1	1	0	1	1	0	1	1	1	0	1	UC	**18**
Dougados (46)	1	1	1	1	1	1	1	1	1	0	1	0	0	UC	UC	1	1	1	1	1	0	UC	1	UC	1	1	UC	**18**
Eckstein (45)	1	1	1	1	2	1	1	1	1	1	1	1	1	1	1	1	1	1	1	1	UC	1	1	1	1	1	0	**26**
Ettinger (44)	1	1	1	1	1	0	1	1	1	1	UC	UC	0	0	UC	1	1	1	UC	1	0	1	1	1	1	1	1	**19**
Filardo (43)	1	1	1	1	2	1	1	1	1	1	1	1	1	1	1	1	1	1	1	1	1	1	1	1	1	1	0	**25**
Hellio le Graverand (69)	1	1	1	1	1	0	1	1	1	1	UC	UC	UC	UC	1	1	UC	1	1	1	0	UC	1	1	UC	1	0	**17**
Henriksen (40)	1	1	1	1	2	1	1	1	0	1	UC	UC	1	0	1	1	1	1	1	1	1	1	1	1	1	1	1	**24**
Hill (5)	1	1	1	1	0	0	1	1	1	1	1	0	UC	1	1	1	1	1	1	1	0	1	1	1	0	1	1	**21**
Hochberg (70)	1	1	1	1	1	1	1	1	1	1	UC	UC	UC	1	UC	1	1	1	UC	1	0	UC	1	1	UC	1	0	**18**
Hoeksma (71)	1	1	1	1	0	1	1	1	1	0	1	1	0	0	1	1	1	1	0	1	1	1	1	1	UC	1	1	**21**
Housman (39)	1	1	1	1	0	0	1	1	1	0	0	UC	0	1	0	1	1	1	UC	1	0	1	1	UC	0	1	1	**16**
Huang (72)	1	1	1	1	0	1	1	0	1	0	UC	UC	UC	UC	1	1	UC	1	1	1	1	UC	1	UC	0	1	1	**16**
Jin (6)	1	1	1	1	0	1	1	1	0	1	UC	UC	0	1	1	1	1	1	UC	1	0	1	1	1	0	1	0	**18**
Karsdal (38)	1	1	1	1	1	0	1	1	1	0	UC	UC	UC	1	1	1	1	1	0	1	0	UC	1	1	1	1	UC	UC
Katz (37)	1	1	1	1	2	1	1	1	1	0	0	0	0	0	0	1	0	1	0	1	0	1	1	0	1	1	UC	**17**
Kim (75)	1	0	1	1	0	1	1	0	1	1	UC	UC	UC	1	1	1	0	1	1	1	UC	1	1	1	0	1	0	**17**

Kinds (18)	1	1	1	1	1	0	1	1	1	1	1	1	1	1	1	1	1	1	1	1	1	1	1	1	1	1	0	**25**
Kongtharvonskul (36)	1	1	1	1	2	1	1	1	0	1	1	UC	0	1	1	1	1	1	0	1	1	1	1	1	1	1	0	**23**
Lequesne (76)	1	1	1	1	1	1	1	1	1	1	UC	UC	0	1	1	1	1	1	1	1	0	UC	1	1	1	1	0	**21**
Lohmander (35)	1	1	1	1	0	1	1	1	1	1	UC	UC	0	1	1	0	1	1	1	1	0	1	1	1	0	1	1	**20**
Maheu (8)	1	1	1	1	0	1	0	1	0	1	UC	UC	0	1	1	1	1	UC	1	1	0	1	1	1	1	0	0	**17**
Marsh (34)	1	1	0	1	2	1	1	0	1	1	UC	UC	1	0	0	1	1	1	UC	1	UC	UC	UC	UC	1	1	0	**16**
McAlindion (33)	1	1	1	1	1	1	1	1	0	1	0	UC	0	1	1	1	1	1	1	1	0	1	1	1	UC	1	0	**20**
Messier (32)	1	1	1	1	1	0	1	1	0	0	0	UC	0	0	1	1	1	1	0	1	UC	1	1	1	1	0	1	**17**
Meissier (77)	1	1	0	1	2	1	1	1	0	1	1	1	1	1	1	1	1	1	1	1	1	1	1	1	1	1	0	**25**
Messier (78)	1	1	1	1	2	1	1	1	0	1	1	1	0	0	1	1	1	1	0	1	1	1	1	UC	1	1	1	**23**
Michel (31)	1	1	1	1	0	0	1	1	0	1	UC	UC	1	1	1	1	1	1	1	1	UC	1	1	1	0	0	1	**19**
Pavelka (30)	1	1	1	0	1	0	1	1	0	1	UC	UC	0	1	1	1	1	1	1	1	0	1	1	UC	1	1	0	**18**
Pavelka (81)	1	1	1	1	1	0	1	1	1	1	1	UC	1	1	1	1	1	1	1	1	1	1	1	1	1	1	1	**25**
Rat (82)	1	1	1	1	1	1	1	1	1	1	1	1	1	1	1	1	1	1	1	1	1	0	1	1	UC	1	1	**25**
Raynauld (27)	1	1	1	1	2	1	1	1	1	1	1	1	1	0	1	1	1	1	1	1	1	1	1	1	1	1	0	**26**
Reginster (83)	1	1	1	1	1	1	1	1	0	1	UC	UC	0	1	1	1	1	1	1	1	0	1	1	1	1	1	1	**22**
Reginster (26)	1	1	1	1	1	0	1	1	0	1	0	UC	1	1	1	1	1	1	1	1	1	1	1	1	UC	1	1	**22**
Roman-Blas (24)	1	1	1	1	1	0	1	1	1	1	UC	UC	0	1	1	1	1	1	0	1	0	UC	1	1	1	1	0	**19**
Rozendaal (31)	1	1	1	1	2	1	1	1	1	0	UC	UC	0	1	1	0	1	1	1	1	UC	1	1	1	1	1	UC	**21**
Sawitzke (86)	1	0	1	1	2	0	1	1	0	1	1	UC	0	1	1	1	1	1	1	1	UC	UC	1	UC	1	UC	UC	UC
Spector (89)	1	1	1	1	2	0	1	1	1	1	UC	UC	0	UC	UC	1	1	1	0	1	0	UC	1	UC	1	1	0	**17**
Sun (90)	1	1	1	1	1	1	1	1	1	1	UC	UC	1	1	1	1	1	1	1	1	UC	1	1	1	1	1	1	**24**
Urish (22)	1	1	1	1	1	1	1	1	1	1	1	1	1	0	1	1	1	1	1	1	1	1	1	1	0	1	0	**24**
Weng (91)	1	1	1	1	0	1	1	0	1	0	UC	UC	1	0	UC	1	1	1	1	1	UC	UC	1	1	0	1	1	**17**
Witt (93)	1	1	1	1	1	1	1	1	1	1	UC	UC	1	0	0	1	1	1	1	1	UC	1	1	1	1	1	1	**22**

UC: Unclear; 2: Yes; 1: Yes/partially; 0: No

Checklist items

1. Is the hypothesis/aim/objective of the study clearly described?
2. Are the main outcomes to be measured clearly described in the Introduction or Methods section?
3. Are the characteristics of the patients included in the study clearly described?
4. Are the interventions of interest clearly described?

Sandhar S, *et al. BMJ Open* 2020; 10:e038720. doi: 10.1136/bmjopen-2020-038720

5. Are the distributions of principal confounders in each group of subjects to be compared clearly described?
6. Are the main findings of the study clearly described?
7. Does the study provide estimates of the random variability in the data for the main outcomes?
8. Have all important adverse events that may be a consequence of the intervention been reported?
9. Have the characteristics of patients lost to follow-up been described?
10. Have actual probability values been reported (e.g. 0.035 rather than <0.05) for the main outcomes except where the probability value is less than 0.001?
11. Were the subjects asked to participate in the study representative of the entire population from which they were recruited?
12. Were those subjects who were prepared to participate representative of the entire population from which they were recruited?
13. Were the staff, places, and facilities where the patients were treated, representative of the treatment the majority of patients receive?
14. Was an attempt made to blind study subjects to the intervention they have received?
15. Was an attempt made to blind those measuring the main outcomes of the Intervention?
16. If any of the results of the study were based on "data dredging", was this made clear?
17. In trials and cohort studies, do the analyses adjust for different lengths of follow-up of patients, or in case-control studies, is the time period between the intervention and outcome the same for cases and controls?
18. Were the statistical tests used to assess the main outcomes appropriate?
19. Was compliance with the intervention/s reliable?
20. Were the main outcome measures used accurate (valid and reliable)?
21. Were the patients in different intervention groups (trials and cohort studies) or were the cases and controls (case-control studies) recruited from the same population?
22. Were study subjects in different intervention groups (trials and cohort studies) or were the cases and controls (case-control studies) recruited over the same period of time?
23. Were study subjects randomized to intervention groups?
24. Was the randomized intervention assignment concealed from both patients and health care staff until recruitment was complete and irrevocable?
25. Was there adequate adjustment for confounding in the analyses from which the main findings were drawn?
26. Were losses of patients to follow-up taken into account?
27. Was there sufficient power to detect treatment effect at significance level of 0.05?
