# Supplementary material for: Risk factors for pain and functional impairment in people with knee and hip osteoarthritis: a systematic review and meta-analysis
Source: BMJ Open. 2020 Aug 7;10(8):e038720. doi: 10.1136/bmjopen-2020-038720 (PMC7418691; doi:10.1136/bmjopen-2020-038720)
Supplement: Author's manuscript [file bmjopen-2020-038720.draft_revisions.pdf]

BMJ Open

BMJ Open is committed to open peer review. As part of this commitment we make the peer review history of every article we publish publicly available.

When an article is published we post the peer reviewers' comments and the authors' responses online. We also post the versions of the paper that were used during peer review. These are the versions that the peer review comments apply to.

The versions of the paper that follow are the versions that were submitted during the peer review process. They are not the versions of record or the final published versions. They should not be cited or distributed as the published version of this manuscript.

BMJ Open is an open access journal and the full, final, typeset and author-corrected version of record of the manuscript is available on our site with no access controls, subscription charges or pay-per-view fees (http://bmjopen.bmj.com).

If you have any questions on BMJ Open's open peer review process please email info.bmjopen@bmj.com

BMJ Open

Risk factors for pain and functional impairment in people with knee and hip osteoarthritis: a systematic review and meta-analysis

Journal:	*BMJ Open*
Manuscript ID	bmjopen-2020-038720
Article Type:	Original research
Date Submitted by the Author:	23-Mar-2020

Complete List of Authors:	Sandhar, Sandeep; University of London St George's, Institute for Infection and Immunity<br>Smith, Toby O.; University of Oxford, Nuffield Department of Orthopaedics and Musculoskeletal Sciences<br>Toor, Kavanbir; University of London St George's, Institute for Infection and Immunity<br>Howe, Franklyn ; University of London St George's, Molecular and Clinical Sciences Research Institute<br>Sofat, Nidhi; University of London St George's, Institute for Infection and Immunity

Keywords:	Knee < ORTHOPAEDIC & TRAUMA SURGERY, Hip < ORTHOPAEDIC & TRAUMA SURGERY, PAIN MANAGEMENT, RHEUMATOLOGY

SCHOLARONE™
Manuscripts

I, the Submitting Author has the right to grant and does grant on behalf of all authors of the Work (as defined in the below author licence), an exclusive licence and/or a non-exclusive licence for contributions from authors who are: i) UK Crown employees; ii) where BMJ has agreed a CC-BY licence shall apply, and/or iii) in accordance with the terms applicable for US Federal Government officers or employees acting as part of their official duties; on a worldwide, perpetual, irrevocable, royalty-free basis to BMJ Publishing Group Ltd ("BMJ") its licensees and where the relevant Journal is co-owned by BMJ to the co-owners of the Journal, to publish the Work in this journal and any other BMJ products and to exploit all rights, as set out in our licence.

The Submitting Author accepts and understands that any supply made under these terms is made by BMJ to the Submitting Author unless you are acting as an employee on behalf of your employer or a postgraduate student of an affiliated institution which is paying any applicable article publishing charge ("APC") for Open Access articles. Where the Submitting Author wishes to make the Work available on an Open Access basis (and intends to pay the relevant APC), the terms of reuse of such Open Access shall be governed by a Creative Commons licence – details of these licences and which Creative Commons licence will apply to this Work are set out in our licence referred to above.

Other than as permitted in any relevant BMJ Author's Self Archiving Policies, I confirm this Work has not been accepted for publication elsewhere, is not being considered for publication elsewhere and does not duplicate material already published. I confirm all authors consent to publication of this Work and authorise the granting of this licence.

For peer review only

Title:

Risk factors for pain and functional impairment in people with knee and hip osteoarthritis: a systematic review and meta-analysis

Concise Title: Factors associated with pain and impaired function in OA

Authors: Sandeep Sandhar (SS) *, Toby Smith (TS) ^, Franklyn Howe (FH) #, Kavanbir Toor (KT) * and Nidhi Sofat (NS) *

Affiliations

*: Institute for Infection and Immunity Research, St Georges, University of London

#: Neurosciences Research Centre, St Georges, University of London

^: Nuffield Department of Orthopaedics, Rheumatology and Musculoskeletal Sciences, University of Oxford

Corresponding author: Professor Nidhi Sofat (nsofat@sgul.ac.uk), Institute for Infection and Immunity, St Georges, University of London, London, SW17 0RE

Work done on behalf of the OA Tech working research group.

Email addresses: ssandhar@sgul.ac.uk (Sandeep Sandhar), toby.smith@ndorms.ox.ac.uk (Toby Smith), howefa@sgul.ac.uk (Franklyn Howe), m1604502@sgul.ac.uk (Kavanbir Toor), nsofat@sgul.ac.uk (Nidhi Sofat)

Article summary
Strengths and limitations of this study
5 bullet points

iew only

ABSTRACT

Objective: Osteoarthritis is the most common form of arthritis. Although prevalent, the factors influencing reporting of symptoms and the progression of disease are not well understood. In this study, we aimed to identify risk factors for pain and functional deterioration in primary knee and hip OA subjects to create a 'stratification tool' for OA development or progression.

Methods: This study followed PRISMA guidelines, searching MEDLINE, EMBASE, CINAHL, MEDLINE and Web of Science (1990-February 2020). The Downs & Black tool assessed methodological quality of selected studies before data extraction. A random-effects or fixed effects meta-analysis was undertaken when study heterogeneity (I^2) was ≥50% or <50% respectively. Standardised mean difference (MD) assessed continuous outcomes with 95% Confidence Intervals (CI), whilst dichotomous variables used odds ratios (OR).

Results: We found 82 studies (41,810 participants) based on our search terms, which were included for analysis. Knee OA pain was associated with: Whole-organ MRI scoring method (WORMS) Knee effusion score ≥1 (OR=1.35,95% CI:0.99,1.83;p=0.05), WORMS Meniscal damage≥1 (OR=1.83, 95% CI:1.23,2.71;p=0.003), Kellgren and Lawrence≥ 2 (MD:2.04, 95% CI:1.48,2.81;p<0.01) and increasing age (MD:1.46,95%CI:0.26,2.66;p=0.02). Predictors for painful hip bone marrow lesion (BML) development were knee pain (MD: -1.42; 95% CI:-1.61,-1.23; p<0.01) and hip pain (MD:-0.72; 95% CI:-0.97, -0.47;p<0.01). Predictors of joint pain in hip OA were large acetabular BMLs (OR=5.23), chronic widespread pain (OR=5.02) and large hip BMLs (OR=4.43).

Conclusions: Our study identified risk factors for clinical pain in OA by imaging measures that can assist in predicting and stratifying subjects with knee/hip OA. A 'stratification tool' combining verified risk factors that we have identified, would allow selective stratification based on pain and structural outcomes in OA.

For peer review only

Article summary

Strengths and limitations of this study

- the factors influencing reporting of symptoms and the progression of osteoarthritis (OA) are not well understood
- we aimed to identify risk factors for pain and functional deterioration in primary knee and hip OA subjects to create a ‘stratification tool’ for OA development or progression
- We found 82 studies with 41,810 participants, which were included for analysis
- Knee OA pain was associated with MRI Knee effusion score ≥1, Meniscal damage≥1 , Kellgren and Lawrence≥ 2 and increasing age. Predictors for painful hip bone marrow lesion development were knee pain and hip pain.
- A ‘stratification tool’ combining verified risk factors that we have identified, would allow selective stratification based on pain and structural outcomes in OA. Future larger prospective studies are now required to validate the risk factors we have identified and assess their impact on interventions for OA.

INTRODUCTION

It has been reported that over 30.8 million US adults suffer from osteoarthritis (OA) (1). Between 1990-2010, the years lived with disability worldwide caused by OA increased from 10.5 million to 17.1 million, an increase of 62.9% (2). Current OA treatment lacks any disease-modifying treatments with a predominance to manage symptoms rather than modify underlying disease (3). The clinical symptoms of OA can be assessed using several questionnaires, the most common of which is the Western Ontario McMaster Arthritic Index (WOMAC) (4, 5, 6). Although pain is a recognised as an important outcome measure in OA, it is not clear what the optimal assessment tools are in OA and how they relate to other risk factors.

OA has various subtypes and since current therapies cannot prevent OA progression, early detection and stratification of those at risk may enable effective pre-symptomatic interventions (7, 8). Several methods are used to define, diagnose and measure OA progression, including imaging techniques [e.g. plain radiography, Computed Tomography (CT) and Magnetic Resonance Imaging (MRI)]. Plain radiography provides high contrast and high resolution images for cortical and trabecular bone, but not for non-ossified structures (e.g. synovial fluid) (9). The most recognised radiographic measure classifying OA severity is Kellgren and Lawrence (KL) grading which assesses osteophytes, joint space narrowing (JSN), sclerosis and bone deformity (10, 11). However, it has been argued that MRI may be more suitable for imaging arthritic joints, providing a whole organ image of the joint (12). Whole-organ MRI scoring method (WORMS) is used in MRI for OA assessing damage, providing a detailed analysis of the joint.

Recently, OMERACT-OARSI (Outcome Measures in Rheumatology-Osteoarthritis Research Society International) have published a core domain set for clinical trials in hip and/or knee OA (13). Six domains were assessed as being mandatory in the assessment of OA, including pain, physical function, quality of life, patient's global assessment of the target joint, and adverse events including mortality and/or joint structure, depending on the intervention tested. However, there still remains

For peer review only

a need to identify risk factors for pain and structural damage in OA so that potential interventions can be studied in a timely manner. In this study, we aimed to identify risk factors for pain, worsening function and structural damage that can predict knee/hip OA development and progression. Our results report a systematic review, with meta-analysis where enough studies were identified for valid comparisons. By identifying risk factors for OA pain and structural damage, tools for stratifying specific disease groups could be developed in the future.

METHODS

This systematic review has been reported in accordance with the PRISMA reporting guidelines. The review protocol was registered *a priori* through PROSPERO (Registration: CRD42018117643).

Search Strategy

A systematic search of the literature was undertaken from 1st January 1990 to 1st February 2020 using electronic databases: MEDLINE (Ovid), EMBASE (Ovid), MEDLINE, Web of Science and CINAHL (EBSCO). An example of the MEDLINE search strategy of included search terms and Boolean operators is presented in Supplementary File 1. Unpublished literature databases including Clinicaltrials.gov and the WHO International Registry of Clinical Trials were also searched, in addition to OpenGrey.

Study Identification

Studies were eligible for inclusion if they were a full text article that satisfied all of the following:

1) 100 or more participants analysed in the study (to increase power for comparisons);
2) convincing definition of OA using American College of Rheumatology criteria;
3) abstract/title that must refer to pain and/or structure in relation to OA as a primary disease;
4) Knee or hip osteoarthritis;

For peer review only

5) pain and/or function scores;

6) joint imaged and

7) minimum 6-month follow-up of pain/function outcome measures.

Non-English studies, letters, conference articles and reviews were excluded.

The titles and abstracts were reviewed by one reviewer (SS). The full-text for each paper was assessed for eligibility by one reviewer (SS) and double-checked by a second (TS). Any disagreements were addressed through discussion and adjudicated by a third reviewer (NS or FH). All studies which satisfied the criteria were included in the review.

Quality assessment

To assess the risk of bias and the power of the methodology, the Downs & Black (D&B) tool was applied (14). These tools assessed the following aspects of each study: reporting quality, external validity, internal validity- bias, selection bias and power. The D&B tool was modified to apply to both interventional and observational studies, resulting in an 'observational Downs and Black tool' (18 items) and an 'interventional downs and black tool' (27 items) (Supplementary File 2). Critical appraisal was performed by one reviewer (SS) and verified by a second (KT). Any disagreements were dealt with by discussion and adjudicated through a third reviewer (TS). In previous literature D&B score ranges were given corresponding quality: excellent (26-28); good (20-25); fair (15-19); and poor (<14) (14). The D&B tool was therefore used to exclude poor quality studies with a score 15/28 or lower in interventional studies and 10/19 or lower in observation studies.

Data extraction

Data were extracted including: subject demographic data, study design, pain and function outcome measures, imaging used, OA severity scores, change in pain and function outcome measures and change in OA severity scores. After all relevant data had been extracted, authors of these papers

For peer review only

were approached to try and attain individual patient data (IPD) related to baseline and change in pain, function and structural scores for each study.

Outcomes

The primary outcome was to determine the development of pain and functional impairment for those with KOA. The secondary outcome was to determine which factors are associated with structural changes in KOA.

Data analysis

All data were assessed for study heterogeneity through scrutiny of the data extraction tables. These identified that there was minimum study-based heterogeneity based on: population, study design and interventions-exposure variabilities for given outcomes. Where there was study heterogeneity, as narrative analysis was undertaken. In this instance, the odds ratio (OR) of all predictor variables were tabulated with a range of OR presented. Where the range did not pass through 1, this was interpreted as significant. Where there was sufficient data to pool and study homogeneity evident, a pooled meta-analysis was deemed appropriate. When I^2 was 50% or greater, a random-effects model meta-analysis was undertaken. When I^2 was less than 50%, a fixed effects model approach was adopted. Continuous outcomes were assessed using standardised mean difference (SMD) scores of measures for developing severe OA, whereas dichotomous variables were assessed through OR data. All data were presented with 95% confidence intervals (CI) and forest-plots.

Due to the presentation of the data, there were minimal data to permit meta-analyses. Where there was insufficient data to pool the analysis, a narrative analysis was undertaken to assess risk factors for the development of increased pain and functional impairment. Analyses were undertaken on STATA version 14.0 (Stata Corp, Texas, USA). Planned subgroup analyses included determine whether there was a difference in risk factors based on (1) anatomical regions (i.e. difference between HOA and KOA); (2) geographical region.

For peer review only

RESULTS

Search Strategy

The results of the search strategy are presented in Figure 1. In total, 11,010 citations were identified. Of these, 141 papers were deemed potentially eligible and screened at full-text level. Of these, 82 met the selected criteria and were included.

Characteristics of Included Studies

A summary of the included studies is presented as Table 1. This consisted of 27 observational studies, 51 RCTs whilst four studies were case-control designs.

In total, 45,767 knees were included in the analysis. This consisted of 13,870 males and 23,497 females; four studies did not report the gender of their cohorts (Valdes, 2012 (15); Kinds, 2012 (16); Davis 2017 (17); Akelman, 2016 (18). Thirty-six studies were undertaken in the USA whilst 30 were undertaken in Europe; seven were performed in Asia and nine were conducted in Australasia. Mean age of the cohorts was 61.7 years (standard deviation: 7.56); 36 studies did not report age (15, 19, 20-52). Mean follow-up period was 35.4 months (SD: 33.6). The most common measures of pain were WOMAC pain (n=55; 50%) and Visual Analogue Scale (VAS) Pain (n=21; 19%). The most frequently used measures of function were WOMAC function (n=52; 44%), physical tests (n=16; 14%) and SF-36 (n=10; 9%).

Methodological Quality

The methodological quality of the evidence was moderate (Supplementary Table 1; Supplementary Table 2). Based on the results of the Downs and Black Observational Studies Checklist, recurrent strengths of the evidence were clear description of the methods adopted (35 studies; 95%), appropriate acknowledgment of principal confounders in each group and their distribution presented (30 studies; 81%) and variability in data presented for the main outcomes (37 studies; 100%). Furthermore the main outcome measures were deemed reliable and valid in all studies (37

For peer review only

studies; 100%) with 89% (33 studies) studies adopting appropriate statistical analyses for their datasets. Recurrent limitations were not clearly reporting the main findings (22 studies; 59%), issues regarding the representation of the cohort from the wider public (19 studies; 51%) and only 8 studies (22%) basing their sample sizes on a prior power calculation.

The results from the Downs and Black non-RCT checklist similarly reported findings with strength of the evidence around clear reporting of the cohort characteristics (43 studies; 98%) and interventions (43 studies; 98%), adoption of reliable/valid outcome measures (41 studies; 93%) and reported high compliance to study processes (41 studies; 91%). Recurrent weaknesses included recruiting cohorts which may not have been reflective of the wider population (38 studies; 86%), in clinic settings which may not have represented typical clinical practice (31 studies; 70%) and poorly adjusting for potential confounders in analyses (31 studies; 70%).

Knee OA systematic review and meta-analysis

Findings from the narrative analysis found the following were predictors for worsening joint pain: KL3 or 4 in women (OR=11.3), a WORMS lateral Meniscal Cyst (MC) score of 1 (OR=4.3), presence of CWP (OR=3.15), increase of ≥2 in WORMS BML score after 15 months (3.2), meniscal maceration (OR=2.82). We also found the following were the highest predictors of worsening function in people with KOA: KL of <3 (OR=3.28), modified KL 3a (OR=1.65), modified KL 4a (OR=1.46), presence of osteophytes (OR=1.31) and female gender (OR=1.79 to 2.06).

Two studies were identified where data could be evaluated for OA risk factors by meta-analysis, namely Guermazi et al. (2010) (39) and Felson *et al.* (2007) (65). A summary of the results is provided in the Forest plots in Figure 2. Results show that female gender, increasing age and the presence of a knee effusion score being ≥1 at baseline were all significantly associated an increased probability of knee OA at statistically significant levels (p<0.05). Interestingly, in this meta-analysis, BMI did not reach statistical significance. The analysis conducted revealed six variables significantly associated

For peer review only

with the development of KOA. As illustrated in Table 2, age (MD:1.46, 95% CI: 0.26 to 2.66; p=0.02; N=823), KL of ≥ 2(MD:2.04, 95% CI: 1.48 to 2.81; p<0.01; N=823) and knee effusion score ≥1 (OR=1.35, 95% CI: 0.99 to 1.83: p=0.05; N=823) were all associated with the development of KOA based on moderate quality evidence. The variables baseline function score (MD:-11.50, 95% CI: -20.73 to -2.27; p=0.01; N=330), cartilage loss graded 2 or more (OR=2.11, 95% CI: 1.18 to 3.79; p=0.01; N=493) and meniscal damage graded 1 or more (OR=1.83, 95% CI: 1.23 to 2.71; p=0.003; N=493) were all associated with OA knee development based on lower quality evidence. The variables of gender (when combining male and female), BML score, ethnicity, BMI and synovitis were not shown to be significantly associated with the KOA development (Table 2).

Hip OA systematic review

We found that baseline knee pain score (MD:-1.42; 95% CI: -1.61 to -1.23; p<0.01; N=198) and baseline hip pain score (MD:-0.72; 95% CI: -0.97 to -0.47; p<0.01; N=198) were both significantly associated with the development of hip BMLs and pain. However, our findings were based on low quality evidence. There was no association between the development of hip BML and BMI or age. Our narrative analysis found predictors for worsening joint pain for people with HOA. This included a large acetabular BML (OR=5.23), a large femoral head BML (OR=4.42) with any large hip BML (OR=4.43), Chronic Widespread Pain (CWP) (OR=5.02), depression (OR=1.90) as significant factors for hip pain development.

DISCUSSION

Our systematic review and meta-analysis identified risk factors for knee and hip OA pain and structural damage based on evaluation of 82 studies meeting inclusion criteria. For the knee, increasing pain in KOA was associated with KL grade 3 or 4 in women, WORMS lateral MC, presence of chronic widespread, increase of ≥2 in WORMS BML score after 15 months and meniscal maceration. The narrative analysis also found that KL<3, KL 3a, KL 4a, osteophyte presence and

For peer review only

female gender were associated with worsening function in people with KOA. In contrast, our meta-analysis of two studies which could be analysed showed that age, radiological features (KL score of 2 or more) and osteophyte presence, knee effusion, poor baseline function, cartilage loss graded 2 or more zones and meniscal tears were associated with development and/or KOA progression.

Our meta-analysis identified risk factors that are appreciated only when results were pooled together. These were namely: WORMS-defined knee effusion score ≥1, cartilage loss graded 2 or more, meniscal damage graded 1 or more and baseline function score. To our knowledge, this is the largest and most up to date systematic review of its kind so far, reviewing 82 primary studies in 41,810 participants.

Some risk factors from our meta-analysis have been recognised previously. For example, Silverwood *et al.* reported previous injuries are associated to developing KOA, supporting the present analysis (93). Kingsbury *et al.* identified age and KL grade as predictive factors for developing KOA, supporting the present findings (94). Therefore the meta-analyses provided both novel and supporting findings for risk factors associated with developing and progressing KOA. A machine learning study assessed risk factors associated with pain and radiological progression in KOA found that BMLs, osteophytes, medial meniscal extrusion, female gender and urine CTX-II contributed to progression (95). Nelson *et al's.* work is supported by other studies (93, 94).

We found large variability in PROM scoring, e.g. Aryal *et al.* scored WOMAC pain from 0-100 (56) while Brandt *et al.* scored from 5-25 (60). Many studies captured differing scoring methods of PROMs. We suggest future studies aim to standardise PROM reporting, supporting Kingsbury *et al's.* conclusions that without standardisation it is difficult to pool data from different trials (94).

After plain radiography, MRI was the most used modality with WORMS as the commonest scoring reported for MRI. The MRI Osteoarthritis Knee Score (MOAKS) (96), expanded on WORMS by scoring entire sub-regions for bone marrow lesions (BMLs) rather than each BML, further division of cartilage regions and refined the features assessed in meniscal morphology**.** Due to this progression

For peer review only

from WORMS, having no MOAKS studies included in our final selection was surprising. This could be due to the eligibility criteria being too restrictive. A future systematic review and meta-analysis focusing on the imaging aspect of evaluating OA will be important.

In HOA, the evaluation of BML size and location is essential in predicting pain progression and these can be assessed effectively using MRI. We recommend that all MRI studies for HOA evaluate BML size and location. Due to the few MRI studies included, further work is needed to determine whether MOAKS or WORMS is the most appropriate scoring system to recommend in KOA studies.

Gait analysis is considered a risk factor for pain/function and was therefore included as a target outcome measure. However, few studies included gait analysis measures, which could not be included in the analysis, perhaps due to the minimum sample size (n=100) being too restrictive.

There were several limitations within our study. Despite identifying novel risk factors for exhibiting KOA, a small dataset was pooled together for the meta-analysis (2 studies) compared to Silverwood *et al.* (34 studies) (93). Silverwood *et al.* targeted non-clinical risk factors which were similarly formatted, permitting increased data pooling, while the present meta-analysis targeted a broad range of clinical variables (93). Our small dataset influenced the GRADE assessment that determined the evidence as low to moderate, restricting the strength of the associations of risk factors with OA development and progression. Further work may impact our confidence in the estimated effect. The eligibility criteria may have been too restrictive, resulting in limited papers including gait analysis or MOAKS. Wet biomarkers were not included in our analyses.

Standardising data collection and reporting is important in conducting meta-analyses. We believe the following should be undertaken to improve data pooling in future work: ensuring group comparisons in studies are selected from the same population (people with confirmed OA) to improve internal validity, observational studies should conduct a power analysis to determine

For peer review only

sample sizes and all studies should include absolute frequency of events data rather than summary odds ratios. Such considerations will improve future meta-analyses to identify OA risk factors.

Our work helps to develop steps towards building a stratification tool for risk factors for knee OA pain and structural damage development. We also highlight the need for collection of core datasets based on defined domains, that has recently also been highlighted by the OMERACT-OARSI core domain set for knee and hip OA (13). Collection of future datasets based on standardised core outcomes will assist in more robust identification of risk factors for large joint OA.

Contributorship statement: SS, TS and KT conducted the information searches and primary data analysis for the study. FAH was involved in conception of the study, reviewing the results and assisting in writing the manuscript, NS conceived the study, contributed to data analysis, obtained funding and reviewed the manuscript.

Data sharing statement: Extra data sharing is available by emailing nsofat@sgul.ac.uk

Ethics: No Ethical Approval was required for this study

Patient and Public Involvement: The research team acknowledges the assistance of both the OA tech network and Engineering and Physical Sciences Research Council. The authors also acknowledge receiving assistance from a meeting that enabled a consensus to be met on the eligibility criteria to be used, and this meeting consisted of the following people: Dr Angela Kedgley, Mrs Abiola Harrison, Professor Alan Boyde, Professor Alan Silman, Dr Amara Ezeonyeji, Miss Caroline Hing, Professor Cathy Holt, Ms Debbie Rolfe, Dr Enrica Papi, Ms Freija Ter Heegde, Mr Jingsong Wang, Dr John Garcia, Dr Mark Elliott, Professor Mary Sheppard, Miss Natasha Kapella, Mr Richard Rendle, Dr Shafaq Sikandar, Dr Sherif Hosny, Miss Soraia Silva, Miss Soraya Koushesh, Miss Susanna Cooper and Dr Thomas Barrick. No writing assistance was used.

Role of Funding Source: This study was funded by the Engineering and Physical Sciences Research Council (EPSRC) under the reference code 'EP/N027264/1' and The Wellcome Trust ISSF award to NS [Grant number 204809/Z/16/Z]. The funder had no input on the study design, data collection and analysis, manuscript preparation or the choice to submit it for publication.

Competing interests: None of the authors had any relation or contact with companies whose products or services may be related to the topic of the article.

For peer review only

REFERENCES

1. Cisternas M, Murphy L, Sacks, J, Solomon D, Pasta D and Helmick C. Alternative Methods for Defining Osteoarthritis and the Impact on Estimating Prevalence in a US Population-Based Survey. Arthritis Care & Research 2016; 68:574-580.
2. Cross M, Smith E, Hoy D, Nolte S, Ackerman I, Fransen M, Bridgett L, et al. The global burden of hip and knee osteoarthritis: estimates from the Global Burden of Disease 2010 study. Annals of the Rheumatic Diseases 2014; 73:1323-1330.
3. Wu Y, Goh E, Wang D and Ma S. Novel treatments for osteoarthritis: a recent update. Open Access Rheumatology: Research and Reviews 2018; 10:135-140.
4. Bellamy N, Buchanan WW, Goldsmith CH, Campbell J and Stitt LW. Validation study of WOMAC: a health status instrument for measuring clinically important patient relevant outcomes to antirheumatic drug therapy in patients with osteoarthritis of the hip or knee. J Rheumatol 1988; 15:1833-40.
5. Kraus V, Blanco F, Englund M, Karsdal M and Lohmander L. Call for standardized definitions of osteoarthritis and risk stratification for clinical trials and clinical use. Osteoarthritis and Cartilage 2015;23:1233-1241
6. Jin X, Jones G, Cicuttini F, Wluka A, Zhu Z, Han W, et al. Effect of Vitamin D Supplementation on Tibial Cartilage Volume and Knee Pain Among Patients With Symptomatic Knee Osteoarthritis: A Randomized Clinical Trial. JAMA 2016; 315:1005-13.
7. Hill CL, March LM, Aitken D, Lester SE, Battersby R, Hynes K, et al. Fish oil in knee osteoarthritis: a randomised clinical trial of low dose versus high dose. Ann Rheum Dis 2016; 75:23-9.
8. Maheu E, Cadet C, Marty M, Moyse D, Kerloch I, Coste P, et al. Randomised, controlled trial of avocado-soybean unsaponifiable (Piascledine) effect on structure modification in hip osteoarthritis: the ERADIAS study. Ann.Rheum.Dis 2014;73:376-84.

For peer review only - http://bmjopen.bmj.com/site/about/guidelines.xhtml

9. Peterfy C. Imaging Techniques. J Klippel, P Dieppe (Eds.), Rheumatology 2E, 1, Mosby, Philadelphia 1998; 1:14.1-14.18

10. Kellgren J and Lawrence J. Radiological Assessment of Osteo-Arthrosis. Annals of the Rheumatic Disease 1957; 16:494-502.

11. Schiphof D, Boers M and Bierma-Zeinstra SM. Differences in descriptions of Kellgren and Lawrence grades of knee osteoarthritis. Annals of the Rheumatic Disease 2008; 67:1034–1036.

12. Peterfy C, Guermazi A, Zaim S, Tirman P Miaux Y, White D, et al. Whole-Organ Magnetic Resonance Imaging Score (WORMS) of the knee in osteoarthritis. Osteoarthritis and Cartilage 2004;12:177-190.

13. Toby O. Smith, Gillian A. Hawker, David J. Hunter. Et al. The OMERACT-OARSI core domain set for measurement in clinical trials of hip and/or knee osteoarthritis. The Journal of Rheumatology 2019, 46(8): 981-989

14. Downs S and Black N. The feasibility of creating a checklist for the assessment of the methodological quality both of randomised and non-randomised studies of health care interventions. Journal of Epidemiology & Community Health 1998;52:377-384.

15. Valdes AM, Doherty SA, Zhang W, Muir KR, Maciewicz RA, Doherty M. Inverse relationship between preoperative radiographic severity and postoperative pain in patients with osteoarthritis who have undergone total joint arthroplasty. Seminars in Arthritis & Rheumatism 2012; 41:568-75.

16. Kinds MB, Marijnissen ACA, Vincken KL, Viergever MA, Drossaers-Bakker KW, Bijlsma JWJ, et al. Evaluation of separate quantitative radiographic features adds to the prediction of incident radiographic osteoarthritis in individuals with recent onset of knee pain: 5-year follow-up in the CHECK cohort. 2012; 20:548-56.

For peer review only

17. Davis J, Eaton CB, Lo GH, Lu B, Price LL, McAlindon TE, et al. Knee symptoms among adults at risk for accelerated knee osteoarthritis: data from the Osteoarthritis Initiative. Clin.Rheumatol 2017;36:1083-9.

18. Akelman MR, Fadale PD, Hulstyn MJ, Shalvoy RM, Garcia A, Chin KE, et al. Effect of Matching or Overconstraining Knee Laxity During Anterior Cruciate Ligament Reconstruction on Knee Osteoarthritis and Clinical Outcomes: A Randomized Controlled Trial With 84-Month Follow-up. Am.J.Sports Med 2016; 44:1660-70.

19. Yu SP, Williams M, Eyles JP, Chen JS, Makovey J, Hunter DJ. Effectiveness of knee bracing in osteoarthritis: pragmatic trial in a multidisciplinary clinic. Int.J.Rheum.Dis. 2016;19:279-86.

20. Urish KL, Keffalas MG, Durkin JR, Miller DJ, Chu CR, Mosher TJ. T2 texture index of cartilage can predict early symptomatic OA progression: data from the osteoarthritis initiative. Osteoarthritis & Cartilage 2013; 21:1550-7.

21. Rozendaal RM, Koes BW, van Osch GJ, Uitterlinden EJ, Garling EH, Willemsen SP, et al. Effect of glucosamine sulfate on hip osteoarthritis: a randomized trial. Ann.Intern.Med 2008; 148:268-77.

22. Roman-Blas JA, Castaneda S, Sanchez-Pernaute O, Largo R, Herrero-Beaumont G, CS/GS Combined Therapy Study Group. Combined Treatment With Chondroitin Sulfate and Glucosamine Sulfate Shows No Superiority Over Placebo for Reduction of Joint Pain and Functional Impairment in Patients With Knee Osteoarthritis: A Six-Month Multicenter, Randomized, Double-Blind, Placebo-Controlled Clinical Trial. Arthritis Rheumatol 2017; 69:77-85.

23. Riddle DL and Jiranek WA. Knee osteoarthritis radiographic progression and associations with pain and function prior to knee arthroplasty: a multicenter comparative cohort study. Osteoarthritis and Cartilage 2015; 23:391-6

For peer review only

24. Reginster JY, Deroisy R, Rovati LC, Lee RL, Lejeune E, Bruyere O, et al. Long-term effects of glucosamine sulphate on osteoarthritis progression: a randomised, placebo-controlled clinical trial. Lancet 2001;357 North American Edition:251-6.

25. Raynauld JP, Martel-Pelletier J, Haraoui B, Choquette D, Dorais M, Wildi LM, et al. Risk factors predictive of joint replacement in a 2-year multicentre clinical trial in knee osteoarthritis using MRI: results from over 6 years of observation. Ann.Rheum.Dis 2011; 70:1382-8.

26. Podsiadlo P., Cicuttini F.M., Wolski M., Stachowiak G.W., Wluka AE. Trabecular bone texture detected by plain radiography is associated with an increased risk of knee replacement in patients with osteoarthritis: A 6 year prospective follow up study. Osteoarthritis and Cartilage 2014; 22:71-5.

27. Pham T, Le Henanff A, Ravaud P, Dieppe P, Paolozzi L, Dougados M. Evaluation of the symptomatic and structural efficacy of a new hyaluronic acid compound, NRD101, in comparison with diacerein and placebo in a 1 year randomised controlled study in symptomatic knee osteoarthritis. Ann.Rheum.Dis 2004; 63:1611-7.

28. Pavelka K., Gatterova J., Gollerova V., Urbanova Z., Sedlackova M., Altman RD. A 5-year randomized controlled, double-blind study of glycosaminoglycan polysulphuric acid complex (Rumalon) as a structure modifying therapy in osteoarthritis of the hip and knee. Osteoarthritis and Cartilage 2000; 8:335-42.

29. Michel BA, Stucki G, Frey D, De Vathaire F, Vignon E, Bruehlmann P, et al. Chondroitins 4 and 6 sulfate in osteoarthritis of the knee: a randomized, controlled trial. Arthritis Rheum 2005; 52:779-86.

30. Messier SP, Loeser RF, Miller GD, Morgan TM, Rejeski WJ, Sevick MA, et al. Exercise and dietary weight loss in overweight and obese older adults with knee osteoarthritis: the Arthritis, Diet, and Activity Promotion Trial. Arthritis Rheum 2004; 50:1501-10.

For peer review only

31. McAlindon T, LaValley M, Schneider E, Nuite M, Lee JY, Price LL, et al. Effect of vitamin D supplementation on progression of knee pain and cartilage volume loss in patients with symptomatic osteoarthritis: a randomized controlled trial. JAMA 2013;309:155-62.

32. Marsh JD, Birmingham TB, Giffin JR, Isaranuwatchai W, Hoch JS, Feagan BG, et al. Cost-effectiveness analysis of arthroscopic surgery compared with non-operative management for osteoarthritis of the knee. BMJ Open 2016; 6:e009949,2015-009949.

33. Lohmander LS, Hellot S, Dreher D, Krantz EF, Kruger DS, Guermazi A, et al. Intraarticular sprifermin (recombinant human fibroblast growth factor 18) in knee osteoarthritis: a randomized, double-blind, placebo-controlled trial. Arthritis Rheumatol 2014; 66:1820-31.

34. Kongtharvonskul J, Woratanarat P, McEvoy M, Attia J, Wongsak S, Kawinwonggowit V, et al. Efficacy of glucosamine plus diacerein versus monotherapy of glucosamine: a double-blind, parallel randomized clinical trial. Arthritis Res.Ther 2016; 18:233,016-1124-9.

35. Katz JN, Brophy RH, Chaisson CE, de Chaves L, Cole BJ, Dahm DL, et al. Surgery versus physical therapy for a meniscal tear and osteoarthritis. N.Engl.J.Med 2013;368:1675-84.

36. Karsdal MA, Byrjalsen I, Alexandersen P, Bihlet A, Andersen JR, Riis BJ, et al. Treatment of symptomatic knee osteoarthritis with oral salmon calcitonin: results from two phase 3 trials. Osteoarthritis and Cartilage 2015; 23:532-43.

37. Housman L, Arden N, Schnitzer TJ, Birbara C, Conrozier T, Skrepnik N, et al. Intra-articular hylastan versus steroid for knee osteoarthritis. Knee Surg.Sports Traumatol. Arthrosc 2014;22:1684-92.

38. Henriksen M., Hunter D.J., Dam E.B., Messier S.P., Andriacchi T.P., Lohmander L.S., et al. Is increased joint loading detrimental to obese patients with knee osteoarthritis? A secondary data analysis from a randomized trial. Osteoarthritis and Cartilage 2013; 21:1865-75.

39. Guermazi A., Hayashi D., Roemer F.W., Niu J., Yang M., Lynch J.A., et al. Cyst-like lesions of the knee joint and their relation to incident knee pain and development of radiographic osteoarthritis: The MOST study. Osteoarthritis and Cartilage 2010; 18:1386-92.

For peer review only

40. Glass NA, Torner JC, Frey Law LA, Wang K, Yang T, Nevitt MC, et al. The relationship between quadriceps muscle weakness and worsening of knee pain in the MOST cohort: a 5-year longitudinal study. Osteoarthritis and Cartilage 2013; 21:1154-9.

41. Filardo G, Di Matteo B, Di Martino A, Merli ML, Cenacchi A, Fornasari P, et al. Platelet-Rich Plasma Intra-articular Knee Injections Show No Superiority Versus Viscosupplementation: A Randomized Controlled Trial. Am.J.Sports Med 2015;43:1575-82.

42. Ettinger WH,Jr, Burns R, Messier SP, Applegate W, Rejeski WJ, Morgan T, et al. A randomized trial comparing aerobic exercise and resistance exercise with a health education program in older adults with knee osteoarthritis. The Fitness Arthritis and Seniors Trial (FAST). JAMA 1997; 277:25-31.

43. Eckstein F, Hitzl W, Duryea J, Kent Kwoh C, Wirth W, OAI investigators. Baseline and longitudinal change in isometric muscle strength prior to radiographic progression in osteoarthritic and pre-osteoarthritic knees--data from the Osteoarthritis Initiative. Arthritis & rheumatology 2013; 21:682-90.

44. Dougados M, Nguyen M, Berdah L, Mazieres B, Vignon E, Lequesne M, et al. Evaluation of the structure-modifying effects of diacerein in hip osteoarthritis: ECHODIAH, a three-year, placebo-controlled trial. Evaluation of the Chondromodulating Effect of Diacerein in OA of the Hip. Arthritis Rheum 2001; 44:2539-47

45. Chandrasekaran S, Gui C, Darwish N, Lodhia P, Suarez-Ahedo C, Domb BG. Outcomes of Hip Arthroscopic Surgery in Patients With Tonnis Grade 1 Osteoarthritis With a Minimum 2-Year Follow-up: Evaluation Using a Matched-Pair Analysis With a Control Group With Tonnis Grade 0. Am.J.Sports Med 2016;44:1781-8.

46. Chandrasekaran S, Darwish N, Gui C, Lodhia P, Suarez-Ahedo C, Domb BG. Outcomes of Hip Arthroscopy in Patients with Tonnis Grade-2 Osteoarthritis at a Mean 2-Year Follow-up: Evaluation Using a Matched-Pair Analysis with Tonnis Grade-0 and Grade-1 Cohorts. Journal of Bone & Joint Surgery 2016; 98:973-82.

For peer review only

47. Campbell DG, Duncan WW, Ashworth M, Mintz A, Stirling J, Wakefield L, et al. Patellar resurfacing in total knee replacement: a ten-year randomised prospective trial. J.Bone Joint Surg.Br 2006;88:734-9.

48. Brown MT, Murphy FT, Radin DM, Davignon I, Smith MD, West CR. Tanezumab reduces osteoarthritic hip pain: results of a randomized, double-blind, placebo-controlled phase III trial. Arthritis Rheum 2013; 65:1795-803.

49. Brown MT, Murphy FT, Radin DM, Davignon I, Smith MD, West CR. Tanezumab reduces osteoarthritic knee pain: results of a randomized, double-blind, placebo-controlled phase III trial. J.Pain 2012; 13:790-8.

50. Bisicchia S, Bernardi G, Tudisco C. HYADD 4 versus methylprednisolone acetate in symptomatic knee osteoarthritis: a single-centre single blind prospective randomised controlled clinical study with 1-year follow-up. Clin.Exp.Rheumatol 2016;34:857-63.

51. Bingham CO,3rd, Buckland-Wright JC, Garnero P, Cohen SB, Dougados M, Adami S, et al. Risedronate decreases biochemical markers of cartilage degradation but does not decrease symptoms or slow radiographic progression in patients with medial compartment osteoarthritis of the knee: results of the two-year multinational knee osteoarthritis structural arthritis study. Arthritis Rheum 2006; 54:3494-507.

52. Ahedi H, Aitken D, Blizzard L, Cicuttini F, Jones G. A population-based study of the association between hip bone marrow lesions, high cartilage signal, and hip and knee pain. Clin.Rheumatol 2014;33:369-76.

53. Amin S, Guermazi A, Lavalley MP, Niu J, Clancy M, Hunter DJ, et al. Complete anterior cruciate ligament tear and the risk for cartilage loss and progression of symptoms in men and women with knee osteoarthritis. Osteoarthritis Cartilage 2008; 16:897-902

54. Antony B, Driban JB, Price LL, Lo GH, Ward RJ, Nevitt M, et al. The relationship between meniscal pathology and osteoarthritis depends on the type of meniscal damage visible on

For peer review only

magnetic resonance images: data from the Osteoarthritis Initiative. Osteoarthritis Cartilage 2017; 25:76-84.

55. Arden NK, Cro S, Sheard S, Dore CJ, Bara A, Tebbs SA, et al. The effect of vitamin D supplementation on knee osteoarthritis, the VIDEO study: a randomised controlled trial. Osteoarthritis Cartilage 2016; 24:1858-66.

56. Ayral X, Mackillop N, Genant HK, Kirkpatrick J, Beaulieu A, Pippingskiold P, et al. Arthroscopic evaluation of potential structure-modifying drug in osteoarthritis of the knee. A multicenter, randomized, double-blind comparison of tenidap sodium vs piroxicam. Osteoarthritis Cartilage 2003;11:198-207.

57. Baselga Garcia-Escudero J and Miguel Hernandez Trillos P. Treatment of Osteoarthritis of the Knee with a Combination of Autologous Conditioned Serum and Physiotherapy: A Two-Year Observational Study. 2015;10:e0145551.

58. Bevers K, Vriezekolk JE, Bijlsma JWJ, van den Ende,Cornelia H M., den Broeder AA. Ultrasonographic predictors for clinical and radiological progression in knee osteoarthritis after 2 years of follow-up. Rheumatology 2015;54:2000-3.

59. Birmingham T.B., Giffin J.R., Chesworth B.M., Bryant D.M., Litchfield R.B., Willits K., et al. Medial opening wedge high tibial osteotomy: A prospective cohort study of gait, radiographic, and patient-reported outcomes. Arthritis Care and Research 2009;61:648-57.

60. Brandt KD, Mazzuca SA, Katz BP, Lane KA, Buckwalter KA, Yocum DE, et al. Effects of doxycycline on progression of osteoarthritis: results of a randomized, placebo-controlled, double-blind trial. Arthritis Rheum 2005;52:2015-25.

61. Bruyere O, Pavelka K, Rovati LC, Deroisy R, Olejarova M, Gatterova J, et al. Glucosamine sulfate reduces osteoarthritis progression in postmenopausal women with knee osteoarthritis: evidence from two 3-year studies. Menopause 2004;11:138-43.

62. Conrozier T, Eymard F, Afif N, Balblanc JC, Legre-Boyer V, Chevalier X, et al. Safety and efficacy of intra-articular injections of a combination of hyaluronic acid and mannitol

For peer review only

(HAnOX-M) in patients with symptomatic knee osteoarthritis: Results of a double-blind, controlled, multicenter, randomized trial. Knee 2016; 23:842-8.

63. Dowsey M.M., Nikpour M., Dieppe P., Choong PFM. Associations between pre-operative radiographic changes and outcomes after total knee joint replacement for osteoarthritis. Osteoarthritis and Cartilage 2012; 20:1095-102.

64. Felson DT, Niu J, Yang T, Torner J, Lewis CE, Aliabadi P, et al. Physical activity, alignment and knee osteoarthritis: data from MOST and the OAI. Osteoarthritis and Cartilage 2013; 21:789-95.

65. Felson DT, Niu J, Guermazi A, Roemer F, Aliabadi P, Clancy M, et al. Correlation of the development of knee pain with enlarging bone marrow lesions on magnetic resonance imaging. Arthritis Rheum 2007; 56:2986-92.

66. Hamilton TW, Pandit HG, Maurer DG, Ostlere SJ, Jenkins C, Mellon SJ, et al. Anterior knee pain and evidence of osteoarthritis of the patellofemoral joint should not be considered contraindications to mobile-bearing unicompartmental knee arthroplasty: a 15-year follow-up. Bone & Joint Journal 2017; 99-B:632-9.

67. Hellio le Graverand MP, Clemmer RS, Redifer P, Brunell RM, Hayes CW, Brandt KD, et al. A 2-year randomised, double-blind, placebo-controlled, multicentre study of oral selective iNOS inhibitor, cindunistat (SD-6010), in patients with symptomatic osteoarthritis of the knee. Ann.Rheum.Dis 2013; 72:187-95.

68. Hochberg MC, Martel-Pelletier J, Monfort J, Moller I, Castillo JR, Arden N, et al. Combined chondroitin sulfate and glucosamine for painful knee osteoarthritis: a multicentre, randomised, double-blind, non-inferiority trial versus celecoxib. Ann.Rheum.Dis 2016; 75:37-44.

69. Hoeksma HL, Dekker J, Ronday HK, Heering A, van der Lubbe N, Vel C, et al. Comparison of manual therapy and exercise therapy in osteoarthritis of the hip: a randomized clinical trial. Arthritis Rheum 2004; 51:722-9.

For peer review only

70. Huang MH, Lin YS, Yang RC, Lee CL. A comparison of various therapeutic exercises on the functional status of patients with knee osteoarthritis. Semin.Arthritis Rheum 2003; 32:398-406.

71. Huizinga MR, Gorter J, Demmer A, Bierma-Zeinstra SMA, Brouwer RW. Progression of medial compartmental osteoarthritis 2-8 years after lateral closing-wedge high tibial osteotomy. Knee Surgery, Sports Traumatology, Arthroscopy 2017; 25:3679-86.

72. Kahn T.L., Soheili A., Schwarzkopf R. Outcomes of Total Knee Arthroplasty in Relation to Preoperative Patient-Reported and Radiographic Measures: Data From the Osteoarthritis Initiative. Geriatric Orthopaedic Surgery and Rehabilitation 2013; 4:117-26.

73. Kim YH, Park JW, Kim JS. The Clinical Outcome of Computer-Navigated Compared with Conventional Knee Arthroplasty in the Same Patients: A Prospective, Randomized, Double-Blind, Long-Term Study. J.Bone Joint Surg.Am 2017; 99:989-96.

74. Lequesne M, Maheu E, Cadet C, Dreiser R. Structural effect of avocado/soybean unsaponifiables on joint space loss in osteoarthritis of the hip. Arthritis & Rheumatism: Arthritis Care & Research 2002; 47:50-8

75. Messier SP, Gutekunst DJ, Davis C, DeVita P. Weight loss reduces knee-joint loads in overweight and obese older adults with knee osteoarthritis. Arthritis Rheum 2005; 52:2026-32.

76. Messier SP, Mihalko SL, Legault C, Miller GD, Nicklas BJ, DeVita P, et al. Effects of intensive diet and exercise on knee joint loads, inflammation, and clinical outcomes among overweight and obese adults with knee osteoarthritis: the IDEA randomized clinical trial. JAMA 2013;310: 1263-73.

77. Muraki S., Akune T., Nagata K., Ishimoto Y., Yoshida M., Tokimura F., et al. Association of knee osteoarthritis with onset and resolution of pain and physical functional disability: The ROAD study. Modern Rheumatology 2014; 24:966-73.

For peer review only

78. Muraki S, Akune T, Nagata K, Ishimoto Y, Yoshida M, Tokimura F, et al. Does osteophytosis at the knee predict health-related quality of life decline? A 3-year follow-up of the ROAD study. Clin.Rheumatol 2015; 34:1589-97.

79. Pavelka K, Gatterova J, Olejarova M, Machacek S, Giacovelli G, Rovati LC. Glucosamine sulfate use and delay of progression of knee osteoarthritis: a 3-year, randomized, placebo-controlled, double-blind study. Arch.Intern.Med 2002; 162:2113-23.

80. Rat A, Baumann C, Guillemin F. National, multicentre, prospective study of quality of life in patients with osteoarthritis of the knee treated with hylane G-F 20. Clin.Rheumatol 2011; 30:1285-93.

81. Reginster JY, Badurski J, Bellamy N, Bensen W, Chapurlat R, Chevalier X, et al. Efficacy and safety of strontium ranelate in the treatment of knee osteoarthritis: results of a double-blind, randomised placebo-controlled trial. Ann.Rheum.Dis 2013; 72:179-86.

82. Romagnoli S. and Marullo M. Mid-Term Clinical, Functional, and Radiographic Outcomes of 105 Gender-Specific Patellofemoral Arthroplasties, With or Without the Association of Medial Unicompartmental Knee Arthroplasty. J.Arthroplasty 2017; 33:688-695

83. Sanchez-Ramirez DC, van der Leeden M, van der Esch M, Roorda LD, Verschueren S, van Dieen J, et al. Increased knee muscle strength is associated with decreased activity limitations in established knee osteoarthritis: Two-year follow-up study in the Amsterdam osteoarthritis cohort. J.Rehabil.Med 2015; 47:647-54.

84. Sawitzke AD, Shi H, Finco MF, Dunlop DD, Harris CL, Singer NG, et al. Clinical efficacy and safety of glucosamine, chondroitin sulphate, their combination, celecoxib or placebo taken to treat osteoarthritis of the knee: 2-year results from GAIT. Ann.Rheum.Dis 2010; 69:1459-64.

85. Skou ST, Wise BL, Lewis CE, Felson D, Nevitt M, Segal NA, et al. Muscle strength, physical performance and physical activity as predictors of future knee replacement: a prospective cohort study. Osteoarthritis & Cartilage 2016; 24:1350-6.

For peer review only

86. Sowers M, Karvonen-Gutierrez CA, Jacobson JA, Jiang Y, Yosef M. Associations of anatomical measures from MRI with radiographically defined knee osteoarthritis score, pain, and physical functioning. Journal of Bone & Joint Surgery 2011; 93:241-51.

87. Spector TD, Conaghan PG, Buckland-Wright JC, Garnero P, Cline GA, Beary JF, et al. Effect of risedronate on joint structure and symptoms of knee osteoarthritis: results of the BRISK randomized, controlled trial [ISRCTN01928173. Arthritis Res.Ther 2005; 7:R625-33.

88. Sun SF, Hsu CW, Lin HS, Liou IH, Chen YH, Hung CL. Comparison of Single Intra-Articular Injection of Novel Hyaluronan (HYA-JOINT Plus) with Synvisc-One for Knee Osteoarthritis: A Randomized, Controlled, Double-Blind Trial of Efficacy and Safety. J.Bone Joint Surg.Am 2017; 99:462-71.

89. Weng MC, Lee CL, Chen CH, Hsu JJ, Lee WD, Huang MH, et al. Effects of different stretching techniques on the outcomes of isokinetic exercise in patients with knee osteoarthritis. Kaohsiung J.Med.Sci 2009; 25:306-15.

90. White DK, Neogi T, Nguyen UDT, Niu J, Zhang Y. Trajectories of functional decline in knee osteoarthritis: the Osteoarthritis Initiative. Rheumatology 2016; 55:801-8.

91. Witt C, Brinkhaus B, Jena S, Linde K, Streng A, Wagenpfeil S, et al. Acupuncture in patients with osteoarthritis of the knee: a randomised trial. Lancet 2005; 366:136-43.

92. Yusuf E, Bijsterbosch J, Slagboom PE, Kroon HM, Rosendaal FR, Huizinga TWJ, et al. Association between several clinical and radiological determinants with long-term clinical progression and good prognosis of lower limb osteoarthritis. PLoS ONE 2011; 6:e25426.

93. Silverwood V, Blagojevic-Bucknall M, Jinks C, Jordan J, Protheroe J and Jordan K. Current evidence on risk factors for knee osteoarthritis in older adults: a systematic review and meta-analysis. Osteoarthritis and Cartilage 2015; 23:507-515.

94. Kingsbury S, Corp N, Watt F, Felson D, O'Neill T, Holt C, et al. Harmonising data collection from osteoarthritis studies to enable stratification: recommendations on core data collection from an Arthritis Research UK clinical studies group. Rheumatology 2016; 55:1394-1402.

For peer review only

95. Nelson A, Fang F, Arbeeva L, Cleveland, R, Schwartz T, Callahan L et al. A machine learning approach to knee osteoarthritis phenotyping: data from the FNIH Biomarkers Consortium. Osteoarthritis and Cartilage 2019; 27:994-1001.

96. Hunter D, Guermazi A, Lo G, Grainger A, Conaghan P, Boudreau R, et al. Evolution of semi-quantitative whole joint assessment of knee OA: MOAKS (MRI Osteoarthritis Knee Score). Osteoarthritis and Cartilage 2011; 19:990-1002.

For peer review only

Table 1: Characteristics of included studies

	**Study Design**	**Number joints (hip/knees)**	**Gender (male:female)**	**Country origin**	**Mean age (years)**	**Follow-up duration (months)**	**Pain outcome measures**	**Functional outcome measures**
Ahedi 2014 (52)	Observational cohort	198 hips	111:87	Australia	UTD	132	WOMAC Pain	NA
Akelman 2016 (18)	RCT	107 knee	UTD	USA	23.5	84	KOOS pain; SF-36 Body pain	SF-36 Physical; AP laxity; IKDC2000
Amin 2008 (53)	Observational cohort	265 knees	152:113	USA	67	30	VAS Pain	WOMAC Function
Antony 2017 (54)	Observational cohort	463 knees	245:218	USA	63	24	WOMAC Pain	NA
Arden 2016 (55)	RCT	474 knees	185:289	UK	64	36	WOMAC Pain	WOMAC Function
Ayral 2003 (56)	RCT	665 knees	259:406	Australia, Belgium, Canada,	61.3	12	WOMAC Pain	WOMAC Function

				Denmark, Finland, France, Hungary, Norway, Spain, United Kingdom U.S.A.				
Baselga Garcia-Escudero 2015 (57)	Observational cohort	118 knees	43:75	Spain	59.1	24	NRS; WOMAC Pain	WOMAC Function
Bevers 2015 (58)	Observational cohort	125 knees	57:68	Netherlands	57	24	WOMAC Pain	WOMAC Function
Bingham 2006 (51)	RCT	2483 knees	735:1748	USA Canada Austria Czech Republic France Germany Hungary Ireland Italy Netherlands Poland Croatia	UTD	24	WOMAC Pain	WOMAC Function
Birmingham 2009 (59)	Observational cohort	126 knees	100:26	Canada	47.5	24	KOOS Pain	KOOS Function; SF-36 Physical; LEFS
Bisicchia 2016 (50)	RCT	150 knees	47:103	Italy	UTD	12	VAS Pain; SF-36	SF-36
Brandt 2005 (60)	RCT	431 knees	0:431	USA	54.9	30	WOMAC Pain; VAS Pain	WOMAC Function
Brown 2012 (49)	RCT	690 knees	270:420	USA	UTD	32 weeks	WOMAC Pain; NRS weekly pain	WOMAC Function; SF-36 Function
Brown 2013 (48)	RCT	621 hips	237:384	USA	UTD	32 weeks	WOMAC Pain	WOMAC Function
Bruyere 2004 (61)	RCT	319 knee	0:319	Belgium	64.0	36	WOMAC Pain	WOMAC Function
Campbell 2006 (47)	RCT	100 knees	28:72	Australia	UTD	120	American Knee Society Score; WOMAC Pain	American Knee Society Score (function); WOMAC Function

For peer review only

Chandrasekaran 2016A (46)	Case-Control	111 hips	66:45	USA	UTD	24	Modified Harris Hip Score; Nonarthritic hip score; VAS Pin	Modified Harris Hip Score; Nonarthritic hip score; Hip Outcome Score; Sports & ADLs
Chandrasekaran 2016B (45)	Case-Control	186 hips	96:90	USA	UTD	24	Modified Harris Hip Score; Nonarthritic hip score; VAS Pin	Modified Harris Hip Score; Nonarthritic hip score; Hip Outcome Score; Sports & ADLs
Conrozier 2016 (62)	RCT	205 knees	88:117	France	65	26	WOMAC Pain; NRS walking pain	WOMAC Function
Davis 2017 (17)	Case-control	3132 knees	UTD	USA	UTD	48	WOMAC Pain; KOOS Pain	WOMAC Function
Dougados 2001 (44)	RCT	507 hips	202:305	France	UTD	36	VAS Pain	Lequesne Index
Dowsey 2012 (63)	Observational cohort	478 knees	147:331	Australia	70.8	24	IKSS Pain	IKSS Function
Eckstein 2013 (43)	RCT	1412 knees	611:801	Austria	UTD	48	WOMAC Pain	NA
Ettinger 1997 (42)	RCT	439 knees	131:308	USA	UTD	18	Pain intensity score	Physical Test
Felson 2013 (64)	Observational cohort	3498 knees	867:1206	USA	61.2	30	WOMAC Pain	PASE
Felson 2007 (65)	Observational cohort	330 knees	111:2111	USA	62.1	15	NA	Quadriceps strength (N)
Filardo 2015 (41)	RCT	183 knees	112:71	Italy	UTD	48	KOOS Pain; IKDC	KOOS Function; TEgner; IKDC
Glass 2013 (40)	Observational cohort	4648 knees	918:1486	USA	UTD	24	WOMAC Pain; NRS Pain	WOMAC Function
Guermazi 2010 (39)	Case-control	493 knees	185:308	USA	UTD	60	WOMAC Pain	PASE

For peer review only

Hamilton 2017 (66)	Observational cohort	805 knees	416:289	UK	66	30	WOMAC Pain	WOMAC Function
Hellio le Graverand 2013 (67)	RCT	1457 knees	343:1114	USA Canada Australia, Belgium, Czech Republic, Germany, Hungary, Italy, Poland, Russian Federation, Slovakia, Spain, Argentina Peru	61.0	180	Oxford Knee Score	Oxford Knee Score; American Knee Society Score; Tegner
Henriksen 2013 (38)	RCT	157 knees	28:129	Denmark	UTD	24	WOMAC Pain	WOMAC Function
Hill 2016 (5)	RCT	202 knees	102:100	Australia	61	12	KOO Pain	KOOS Function and kinematic assessment
Hochberg 2016 (68)	RCT	522 knees	84:438	France Germany Poland Spain	62.7	24	WOMAC Pain	WOMAC Function
Hoeksma 2004 (69)	RCT	109 hips	33:76	Netherlands	72	6	WOMAC T Pain; Huskisson's VAS; EQ-5D Pain	WOMAC Function; EQ-5D Function
Housman 2014 (37)	RCT	391 knees	130:261	USA Canada France UK Germany	UTD	6	SF-36 Body Pain; Harris Hip Score; VAS Pain	SF-36 Function; Harris Hip Score; ROM
Huang 2003 (70)	RCT	264 knees	39:93	Taiwan	62	6	WOMAC Pain	NA
Huizinga 2017 (71)	Observational cohort	298 knees	201:97	Netherlands	51	12	VAS Pain	Lequesne index; Walking speed
Jin 2016 (6)	RCT	413 knees	205:208	Australia	63.2	24	WOMAC Pain; VAS Pain	WOMAC Function

For peer review only

Kahn 2013 (72)	Observational cohort	174 knees	70:102	USA	67.0	6	WOMAC Pain	WOMAC Function
Karsdal 2015 (36)	RCT	2207 knees	773:1424	Denmark	UTD	24	WOMAC Pain	WOMAC Function
Katz 2013 (35)	RCT	330 knees	143:187	USA	UTD	12	KOO Pain	WOMAC Function; SF-36 Function
Kim 2017 (73)	RCT	352 knees	9:153	Republic of Korea	68.1	144	WOMAC	Knee Society Knee Score Function; ROM; UCLA Activity
Kinds 2012 (16)	RCT	565 knees	UTD	Netherlands	UTD	60	WOMAC Pain	WOMAC Function
Kongtharvonskul 2016 (34)	RCT	148 knees	25:123	Thailand	UTD	6	WOMAC Pain; VAS Pain	WOMAC Function
Lequesne 2002 (74)	RCT	163 hips	102:61	France	63.2	24	VAS Pain	Lequesne Index
Lohmander 2014 (33)	RCT	170 knees	52:116	Bulgaria Canada Croatia Finland Germany Poland Serbia Africa Sweden USA	UTD	12	WOMAC Pain	WOMAC Function
Maheu 2014 (8)	RCT	345 hips	159:186	France	62.2	36	WOMAC Pain; Global Hip Pain	Lequesne Index; WOMAC Function; Global handicap NRS
Marsh 2016 (32)	RCT	168 knees	57:112	Canada	UTD	24	WOMAC	WOMAC
McAlindion 2013 (31)	RCT	146 knees	57:89	USA	UTD	24	WOMAC Pain	WOMAC Function; Physical Test
Messier 2004 (30)	RCT	316 knees	89:227	USA	UTD	18	WOMAC Pain	WOMAC Function; Physical Test
Messier 2005 (75)	RCT	142 knees	37:105	USA	68.5	18	WOMAC Pain	WOMAC Function; Physical Test

For peer review only

Messier 2013 (76)	RCT	454 knees	128:325	USA	66	18	WOMAC Pain	WOMAC Function; Physical Test; SF-36 Physical
Michel 2005 (29)	RCT	300 knees	146:154	Switzerland	UTD	24	WOMAC Pain	WOMAC Function; Physical Test
Muraki 2014 (77)	Observational cohort	1558 knees	553:1005	Japan	67.0	40	WOMAC Pain	WOMAC Function;
Muraki 2015 (78)	Observational cohort	1525 knees	546:979	Japan	67.0	40	WOMAC Pain	WOMAC Function
Pavelka 2000 (28)	RCT	277 knees; 117 hips	109:285	Czech Republic	58	60	NA	Lequesne Index
Pavelka 2002 (79)	RCT	202 knees	45:157	Czech Republic	UTD	36	WOMAC Pain	WOMAC Function; Lequesne Index
Pham 2004 (27)	Observational cohort	301 knees	97:204	France	UTD	12	VAS Pain	Lequesne Index
Podsiadlo 2014 (26)	Observational cohort	114 knees	49:65	Australia	UTD	72	WOMAC Pain	WOMAC Function
Rat 2011 (80)	RCT	300 knees	118:182	France	67	6	SF-36 Body Pain; OAKHQOL Pain; VAS Pain	Lequense Index; SF-36 Physical; OAKHQOL Physical Activity
Raynauld 2011 (25)	RCT	123 knees	44:79	Canada	UTD	24	WOMAC Pain	WOMAC Function
Reginster 2001 (24)	RCT	212 knees	50:162	Belgium	UTD	36	WOMAC Pain	WOMAC Function
Reginster 2013 (81)	RCT	1371 knees	425:946	Australia Austria Belgium Canada Czech Republic Denmark Estonia France Germany Italy Lithuania Netherlands Poland Portugal Romania Russian Federation	62.9	36	WOMAC Pain; VAS Pain	WOMAC Function

				Spain United Kingdom				
Riddle 2015 (23)	Observational cohort	467 knees	209:258	USA	UTD	24	KOOS Pain	WOMAC Function
Romagnoli 2017 (82)	Observational cohort	105 knees	16:69	Italy	67.7	66	Knee Society Score Clinical; VAS Pain	Knee Society Score Function; ROM
Roman-Blas 2017 (22)	RCT	158 knees	26:132	Spain	UTD	6	WOMAC Pain; VAS Pain	WOMAC Function
Rozendaal 2008 (21)	RCT	222 hips	68:154	Netherlands	UTD	24	WOMAC Pain; VAS Pain	WOMAC Function
Sanchez-Ramirez 2015 (83)	Observational cohort	186 knees	59:127	Canada	61	24	WOAMC Pain	WOMAC Function; Physical Test
Sawitzke 2010 (84)	RCT	662 knees	215:447	USA	57	24	WOMAC Pain	WOMAC Function
Skou 2016 (85)	Observational cohort	1682 knees	434:818	Denmark	62.2	84	WOMAC Pain	PASE; Physical Test
Sowers 2011 (86)	Observational cohort	724 knees	0:363	USA	56	132	NA	WOMAC Function; Physical Test
Spector 2005 (87)	RCT	284 knees	115:169	UK	63.3	12	WOMAC Pain	WOMAC Function
Sun 2017 (88)	RCT	121 knees	31:90	Taiwan	63	6	WOMAC Pain; VAS Pain	WOMAC Function; Lequesne Index; Physical Test
Urish 2013 (20)	RCT	336 knees	96:67	USA	UTD	36	WOMAC	WOMAC
Valdes 2012 (15)	Observational cohort	860 knees; 928 hips	UTD	UK	UTD	38	WOMAC Pain	NA
Van der Esch 2016 (96)	Observational cohort	402 knees	64:137	Netherlands	61.2	24	NRS Pain	WOMAC Function; Physical Test
Weng 2009 (89)	RCT	264 knees	26:106	Taiwan	64	12	VAS Pain	Lequesne Index; ROM; Physical Test
White 2016 (90)	Observational cohort	2110 knees	992:118	USA	61.0	84	VAS Pain	WOMAC Function
Witt 2005 (91)	RCT	294 knees	70:154	Germany	64.0	12	WOMAC Pain; SF-	WOMAC Function;

For peer review only

							36 Body Pain; VAS Pain	SF-36 Function
Yu 2016 (19)	Observational cohort	204 knees	74:130	Australia	UTD	12	KOOS Pain; VAS Pain	KOOS ADL; Physical Function
Yusuf 2011 (92)	Observational cohort	74 knees; 31 hips; 11 hip and knees	19:98	Netherlands	60	72	WOMAC Pain; SF-36 Body Pain; Pain on movement	WOMAC Function; SF-36 Function; Physical Test

For peer review only

Table 2. Meta-Analysis Results: Exhibit Knee OA

Exhibiting knee OA was defined as having no knee pain at baseline but presence of knee pain in both follow-up assessments for both Felson *et al.* 2007 (65) and Guermazi *et al.* 2010 (39). Studies used for analysis on: Gender, age, KL≥2, Knee effusion score ≥1 and BMI (39,65). Studies used for analysis on: BML score and baseline function score (39, 65).
Studies used for analysis on: Ethnicity, BML≥1, Synovitis ≥1, Cartilage loss ≥2 and Meniscal damage ≥1 (39).

Felson *et al* 2007 (65) measured all non-demographic variables at both baseline and 15 months while Guermazi *et al* 2010 (39) measured all non-demographic variables at baseline, 15 months and 30 months

**Variable**	**N**	**Effect Estimate**	**P-Value**	**Statistical Heterogeneity** (I^2%)	**GRADE Assessment**
Gender	823	0.91 (0.48 to 1.72)*	0.78	87	Low quality evidence[1]
Age	823	1.46 (0.26 to 2.66)	0.02	0	Moderate quality evidence[2]
KL ≥2	823	2.04 (1.48 to 2.81)	<0.01	35	Moderate quality evidence[2]
BML Score	330	0.40 (-0.11 to 0.91)	0.13	NE	Low quality evidence[1]
Knee effusion score ≥1	823	1.35 (0.99 to 1.83)	0.05	0	Moderate quality evidence[2]
Baseline function score	330	-11.50 (-20.73 to -2.27)	0.01	NE	Low quality evidence[1]
Ethnicity (white)	493	1.03 (0.59 to 1.82)	0.91	NE	Low quality evidence[1]
BMI	823	-0.08 (-0.75 to 0.58)	0.81	0	Moderate quality evidence[2]
BML ≥1	493	1.44 (0.94 to 2.21)	0.09	NE	Low quality evidence[1]
Synovitis ≥1	493	1.24 (0.83 to 1.85)	0.30	NE	Low quality evidence[1]
Cartilage loss ≥2	493	2.11 (1.18 to 3.79)	0.01	NE	Low quality evidence[1]
Meniscal damage ≥1	493	1.83 (1.23 to 2.71)	0.003	NE	Low quality evidence[1]

* - random effects model analysis; NE – not estimable

[1]GRADE – Outcomes downgraded one level due to risk of bias, two level due to imprecision and inconsistency; [2]GRADE – Outcomes downgraded one level due to risk of bias

For peer review only

Figure legends

Figure 1: PRISMA flow-chart

Figure 2: A) Forest plot illustrating the probability of knee osteoarthritis by gender, **B)** Forest plot illustrating the difference in age between people with and without knee osteoarthritis, **C)** Forest plot illustrating the probability of knee osteoarthritis by knee effusion score being ≥1 at baseline and **D)** Forest plot illustrating the probability of knee osteoarthritis by BMI

Table 1: Characteristics of included studies

Supplementary Table 1: Methodological appraisal results based on the Downs and Black Observational Studies Checklist

Supplementary Table 2: Methodological appraisal results based on the Downs and Black Interventional (non-RCT) Studies Checklist

For peer review only

For peer review only - http://bmjopen.bmj.com/site/about/guidelines.xhtml

Figure 1

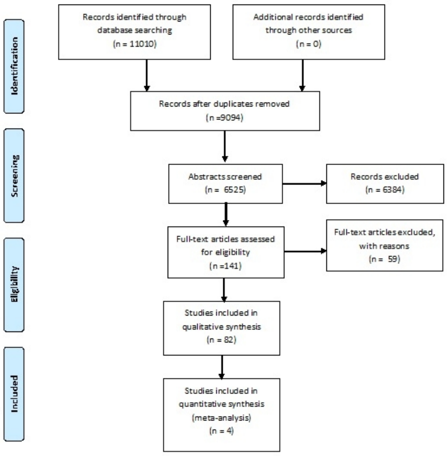

304x190mm (96 x 96 DPI)

Figure 2

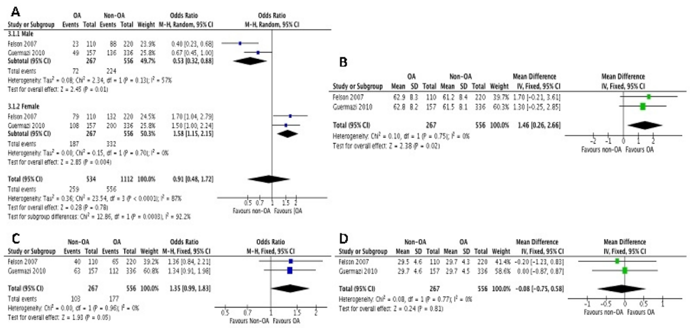

304x190mm (96 x 96 DPI)

Supplementary Table 1: Methodological appraisal results based on the Downs and Black Observational Studies Checklist

	Downs and Black Observational Studies Checklist Items																		
	**1**	**2**	**3**	**4**	**5**	**6**	**7**	**8**	**9**	**10**	**11**	**12**	**13**	**14**	**15**	**16**	**17**	**18**	**Total**
Ahedi 2014	1	1	1	0	1	1	0	1	1	1	1	UTD	1	1	1	UTD	1	0	13
Amin 2008	1	1	0	1	1	1	1	0	0	UTD	1	1	1	1	UTD	1	1	0	12
Antony 2017	1	1	1	2	1	1	1	0	1	0	UTD	UTD	1	1	1	UTD	1	0	13
Baselga Garcia-Escudero 2015	1	1	1	0	1	1	1	1	UTD	UTD	1	1	1	1	UTD	0	1	1	13
Bevers 2015	1	1	1	2	0	1	1	1	UTD	0	1	1	1	1	1	1	1	0	15
Birmingham 2009	1	1	1	1	1	1	1	1	1	1	UTD	1	1	UTD	1	1	1	0	15
Chandrasekaran 2016A	1	1	1	1	1	1	1	1	0	UTD	1	1	1	1	UTD	1	1	1	15
Chandrasekaran 2016B	1	1	1	1	1	1	0	1	0	UTD	1	1	1	1	UTD	1	UTD	1	13
Davis 2017	1	1	1	0	0	1	1	0	1	1	1	UTD	1	1	1	UTD	1	0	12
Dowsey 2012	1	1	1	1	1	1	1	1	1	1	1	UTD	1	1	1	1	1	0	16
Eckstein 2013	1	1	1	2	1	1	1	1	1	1	1	1	1	UTD	1	1	1	0	17
Felson 2013	1	1	1	1	0	1	1	1	0	UTD	1	1	1	UTD	1	1	1	0	13
Filardo 2015	1	1	1	2	0	1	1	1	1	1	1	1	1	UTD	1	1	1	0	16
Glass 2013	1	1	1	2	0	1	1	1	1	1	1	1	1	1	1	1	1	0	17

For peer review only

Guermazin 2010	1	1	1	2	0	1	1	1	1	1	1	1	1	UTD	1	1	1	0	16
Hamilton 2017	1	1	0	0	1	1	1	1	UTD	UTD	1	1	1	1	UTD	UTD	1	1	12
Henriksen 2013	1	1	1	2	1	1	1	1	UTD	UTD	1	1	1	UTD	UTD	1	1	1	15
Huizinga 2017	1	1	1	0	1	1	1	0	UTD	UTD	1	1	1	1	UTD	0	1	0	11
Khan 2013	1	1	1	1	0	1	1	1	1	1	0	1	1	UTD	1	1	1	0	14
Kinds 2012	1	1	1	1	0	1	1	1	1	1	1	1	1	UTD	1	1	1	0	15
Messier 2005	1	1	0	2	1	1	0	1	1	UTD	1	1	1	UTD	1	1	1	0	14
Muraki 2014	1	1	1	1	1	1	1	1	1	UTD	1	1	1	1	0	1	1	0	15
Muraki 2015	1	1	1	2	1	1	1	1	1	UTD	1	1	1	1	0	1	1	0	16
Podsiadlo 2014	1	1	1	1	0	1	1	1	UTD	UTD	1	1	1	UTD	UTD	1	1	0	12
Rat 2011	1	1	1	1	1	1	1	1	UTD	UTD	1	1	1	1	0	UTD	1	1	14
Raynauld 2011	1	0	1	2	1	1	1	1	1	UTD	1	1	1	1	1	1	1	0	16
Riddle 2015	1	1	1	2	1	1	0	0	1	1	0	1	1	1	1	1	1	1	16
Romagnoli 2017	1	0	1	0	0	1	1	1	1	1	1	1	1	UTD	1	UTD	1	1	13
Sanchez-Ramirez 2015	1	1	1	2	1	1	1	1	1	1	1	1	1	UTD	1	1	1	0	17
Skou 2016	1	1	1	2	0	1	1	1	1	1	1	1	1	UTD	1	1	1	0	16
Sowers 2011	1	1	1	0	1	1	1	1	1	UTD	1	1	1	UTD	1	0	0	0	12

For peer review only

For peer review only - http://bmjopen.bmj.com/site/about/guidelines.xhtml

Urish 2013	1	1	1	1	1	1	1	1	1	1	1	1	1	1	1	0	1	0	16
Valder 2012	1	1	1	1	1	1	0	1	UTD	UTD	1	1	1	0	1	1	0	0	12
Van der Esch 2016	1	1	1	1	1	1	1	1	1	1	1	1	1	UTD	1	0	1	0	15
White 2016	1	1	1	2	0	1	1	0	1	1	1	1	1	1	0	1	1	0	15
Yu 2016	1	1	1	1	1	1	1	1	1	1	1	1	1	1	1	1	1	0	17
Yusuf 2011	1	1	1	1	1	1	1	0	UTD	UTD	1	1	1	UTD	1	1	1	0	13
Total with score >0	37	35	34	30	15	37	32	30	24	18	33	33	37	20	25	26	34	8	-

For peer review only

Checklist items

1. Is the hypothesis/aim/objective of the study clearly described?
2. Are the main outcomes to be measured clearly described in the Introduction or Methods section?
3. Are the characteristics of the patients included in the study clearly described?
4. Are the distributions of principal confounders in each group of subjects to be compared clearly described?
5. Are the main findings of the study clearly described?
6. Does the study provide estimates of the random variability in the data for the main outcomes?
7. Have the characteristics of patients lost to follow-up been described?
8. Have actual probability values been reported (e.g. 0.035 rather than <0.05) for the main outcomes except where the probability value is less than 0.001?
9. Were the subjects asked to participate in the study representative of the entire population from which they were recruited?
10. Were those subjects who were prepared to participate representative of the entire population from which they were recruited?
11. If any of the results of the study were based on “data dredging”, was this made clear?
12. Were the statistical tests used to assess the main outcomes appropriate?
13. Were the main outcome measures used accurate (valid and reliable)?
14. Were study participants in different groups (trials and cohort studies) or were the cases and controls (case-control studies) recruited over the same period of time?

15. Were the participants in different groups (trials and cohort studies) or were the cases and controls (case-control studies) recruited from the same population?
16. Was there adequate adjustment for confounding in the analyses from which the main findings were drawn?
17. Were losses of patients to follow-up taken into account?
18. Did the study mention having conducted a power analysis to determine the sample size needed to detect a significant difference in effect size for one or more outcome measures?

Footnote

UTD: Unable To Determine
2: Yes
1: Yes/partially
0: No

For peer review only

Supplementary Table 2: Methodological appraisal results based on the Downs and Black Interventional (non-RCT) Studies Checklist

	Downs and Black Interventional Studies Checklist Items																											
	1	2	3	4	5	6	7	8	9	10	11	12	13	14	15	16	17	18	19	20	21	22	23	24	25	26	27	**Total**
Akelman 2016	1	1	1	1	2	1	1	1	1	1	1	1	1	1	1	1	1	1	1	1	0	1	1	1	UTD	1	1	**26**
Arden 2016	1	1	1	1	1	1	1	1	1	0	1	1	1	1	1	1	UTD	1	1	1	1	1	1	1	0	1	UTD	**23**
Ayral 2003	1	1	1	1	1	1	1	1	1	1	0	UTD	UTD	1	1	1	1	1	1	1	0	1	1	UTD	UTD	1	0	**20**
Bingham 2006	1	1	1	1	1	1	1	1	1	1	1	1	1	1	UTD	1	1	1	1	1	0	1	0	UTD	1	1	0	**22**
Bisicchia 2016	1	0	1	1	0	1	1	1	1	1	1	1	1	0	1	UTD	1	1	1	1	1	1	1	0	0	1	0	**20**
Brandt 2005	1	1	1	1	1	0	1	1	1	1	UTD	UTD	UTD	UTD	1	1	1	1	1	1	UTD	1	1	UTD	0	1	0	**18**
Brown 2013	1	1	1	1	1	1	1	1	1	0	UTD	UTD	UTD	1	1	1	1	1	1	1	UTD	UTD	1	UTD	UTD	UTD	1	**18**
Brown 2012	1	1	1	1	1	1	1	1	1	0	UTD	UTD	UTD	1	1	1	1	UTD	1	1	UTD	UTD	1	1	UTD	1	1	**19**
Bruyere 2004	1	1	1	1	1	0	1	0	1	1	UTD	UTD	1	1	1	1	1	1	UTD	1	UTD	UTD	1	UTD	UTD	1	1	**18**
Campbell 2006	1	1	1	1	0	0	0	1	1	0	1	UTD	1	1	1	1	1	1	1	1	1	1	1	1	UTD	1	0	**20**
Conrozier 2016	1	1	1	1	0	0	1	1	1	1	UTD	UTD	UTD	1	1	1	1	0	1	1	0	1	1	1	0	1	UTD	**18**
Dougados 2001	1	1	1	1	1	1	1	1	1	0	1	0	0	UTD	UTD	1	1	1	1	1	0	UTD	1	UTD	1	1	UTD	**18**
Ettinger 1997	1	1	1	1	1	0	1	1	1	1	UTD	UTD	0	0	UTD	1	1	1	UTD	1	0	1	1	1	1	1	1	**19**
Hellio le Graverand 2013	1	1	1	1	1	0	1	1	1	1	UTD	UTD	UTD	UTD	1	1	UTD	1	1	1	0	UTD	1	1	UTD	1	0	**17**
Hill 2016	1	1	1	1	0	0	1	1	1	1	1	0	UTD	1	1	1	1	1	1	1	0	1	1	1	0	1	1	**21**
Hochberg 2016	1	1	1	1	1	1	1	1	1	1	UTD	UTD	UTD	1	UTD	1	1	1	UTD	1	0	UTD	1	1	UTD	1	0	**18**
Hoeksma 2004	1	1	1	1	0	1	1	1	1	0	1	1	0	0	1	1	1	1	0	1	1	1	1	1	UTD	1	1	**21**
Housman 2014	1	1	1	1	0	0	1	1	1	0	0	UTD	0	1	0	1	1	1	UTD	1	0	1	1	UTD	0	1	1	**16**
Huang 2003	1	1	1	1	0	1	1	0	1	0	UTD	UTD	UTD	UTD	1	1	UTD	1	1	1	1	UTD	1	UTD	0	1	1	**16**
Jin 2016	1	1	1	1	0	1	1	1	0	1	UTD	UTD	0	1	1	1	1	1	UTD	1	0	1	1	1	0	1	0	**18**
Karsdal 2015	1	1	1	1	1	0	1	1	1	0	UTD	UTD	UTD	1	1	1	1	1	0	1	0	UTD	1	1	1	1	UTD	**18**
Katz 2013	1	1	1	1	2	1	1	1	1	0	0	0	0	0	0	1	0	1	0	1	0	1	1	0	1	1	UTD	**17**
Kim 2017	1	0	1	1	0	1	1	0	1	1	UTD	UTD	UTD	1	1	1	0	1	1	1	UTD	1	1	1	0	1	0	**17**
Kongtharvonskul 2016	1	1	1	1	2	1	1	1	0	1	1	UTD	0	1	1	1	1	1	0	1	1	1	1	1	1	1	0	**23**
Lequesne 2002	1	1	1	1	1	1	1	1	1	1	UTD	UTD	0	1	1	1	1	1	1	1	0	UTD	1	1	1	1	0	**21**
Lohmander 2014	1	1	1	1	0	1	1	1	1	1	UTD	UTD	0	1	1	0	1	1	1	1	0	1	1	1	0	1	1	**20**
Maheu 2014	1	1	1	1	0	1	0	1	0	1	UTD	UTD	0	1	1	1	1	UTD	1	1	0	1	1	1	1	0	0	**17**
Marsh 2016	1	1	0	1	2	1	1	0	1	1	UTD	UTD	1	0	0	1	1	1	UTD	1	UTD	UTD	UTD	UTD	1	1	0	**16**
McAlindion 2013	1	1	1	1	1	1	1	1	0	1	0	UTD	0	1	1	1	1	1	1	1	0	1	1	1	UTD	1	0	**20**
Messier 2004	1	1	1	1	1	0	1	1	0	0	0	UTD	0	0	1	1	1	1	0	1	UTD	1	1	1	1	0	1	**17**
Messier 2013	1	1	1	1	2	1	1	1	0	1	1	1	0	0	1	1	1	1	0	1	1	1	1	UTD	1	1	1	**23**
Michel 2005	1	1	1	1	0	0	1	1	0	1	UTD	UTD	1	1	1	1	1	1	1	1	UTD	1	1	1	0	0	1	**19**
Pavelka 2000	1	1	1	0	1	0	1	1	0	1	UTD	UTD	0	1	1	1	1	1	1	1	0	1	1	UTD	1	1	0	**18**
Pavelka 2002	1	1	1	1	1	0	1	1	1	1	1	UTD	1	1	1	1	1	1	1	1	1	1	1	1	1	1	1	**25**
Pham 2004	1	1	1	1	0	0	1	1	1	1	UTD	UTD	0	1	1	1	1	1	UTD	1	0	UTD	1	1	0	1	0	**17**
Reginster 2013	1	1	1	1	1	1	1	1	0	1	UTD	UTD	0	1	1	1	1	1	1	1	0	1	1	1	1	1	1	**22**
Reginster 2001	1	1	1	1	1	0	1	1	0	1	0	UTD	1	1	1	1	1	1	1	1	1	1	1	1	UTD	1	1	**22**
Roman-Blas 2017	1	1	1	1	1	0	1	1	1	1	UTD	UTD	0	1	1	1	1	1	0	1	0	UTD	1	1	1	1	0	**19**
Rozendaal 2008	1	1	1	1	2	1	1	1	1	0	UTD	UTD	0	1	1	0	1	1	1	1	UTD	1	1	1	1	1	UTD	**21**
Sawitzke 2010	1	0	1	1	2	0	1	1	0	1	1	UTD	0	1	1	1	1	1	1	1	UTD	UTD	1	UTD	1	UTD	UTD	**18**
Spector 2005	1	1	1	1	2	0	1	1	1	1	UTD	UTD	0	UTD	UTD	1	1	1	0	1	0	UTD	1	UTD	1	1	0	**17**
Sun 2017	1	1	1	1	1	1	1	1	1	1	UTD	UTD	1	1	1	1	1	1	1	1	UTD	1	1	1	1	1	1	**24**
Weng 2009	1	1	1	1	0	1	1	0	1	0	UTD	UTD	1	0	UTD	1	1	1	1	1	UTD	UTD	1	1	0	1	1	**17**
Witt 2005	1	1	1	1	1	1	1	1	1	1	UTD	UTD	1	0	0	1	1	1	1	1	UTD	1	1	1	1	1	1	**22**
Total score >0	44	41	43	43	30	26	42	39	33	31	12	6	13	30	34	41	39	41	29	44	9	29	42	29	13	39	19	-

Checklist items

1. Is the hypothesis/aim/objective of the study clearly described?
2. Are the main outcomes to be measured clearly described in the Introduction or Methods section?
3. Are the characteristics of the patients included in the study clearly described?
4. Are the interventions of interest clearly described?
5. Are the distributions of principal confounders in each group of subjects to be compared clearly described?
6. Are the main findings of the study clearly described?
7. Does the study provide estimates of the random variability in the data for the main outcomes?
8. Have all important adverse events that may be a consequence of the intervention been reported?
9. Have the characteristics of patients lost to follow-up been described?
10. Have actual probability values been reported (e.g. 0.035 rather than <0.05) for the main outcomes except where the probability value is less than 0.001?
11. Were the subjects asked to participate in the study representative of the entire population from which they were recruited?
12. Were those subjects who were prepared to participate representative of the entire population from which they were recruited?
13. Were the staff, places, and facilities where the patients were treated, representative of the treatment the majority of patients receive?
14. Was an attempt made to blind study subjects to the intervention they have received?
15. Was an attempt made to blind those measuring the main outcomes of the Intervention?
16. If any of the results of the study were based on “data dredging”, was this made clear?
17. In trials and cohort studies, do the analyses adjust for different lengths of follow-up of patients, or in case-control studies, is the time period between the intervention and outcome the same for cases and controls?
18. Were the statistical tests used to assess the main outcomes appropriate?
19. Was compliance with the intervention/s reliable?
20. Were the main outcome measures used accurate (valid and reliable)?
21. Were the patients in different intervention groups (trials and cohort studies) or were the cases and controls (case-control studies) recruited from the same population?
22. Were study subjects in different intervention groups (trials and cohort studies) or were the cases and controls (case-control studies) recruited over the same period of time?
23. Were study subjects randomized to intervention groups?
24. Was the randomized intervention assignment concealed from both patients and health care staff until recruitment was complete and irrevocable?
25. Was there adequate adjustment for confounding in the analyses from which the main findings were drawn?
26. Were losses of patients to follow-up taken into account?
27. Was there sufficient power to detect treatment effect at significance level of 0.05?

For peer review only

Footnote

UTD: Unable To Determine
2: Yes
1: Yes/partially
0: No

For peer review only

BMJ Open

Risk factors for pain and functional impairment in people with knee and hip osteoarthritis: a systematic review and meta-analysis

Journal:	*BMJ Open*
Manuscript ID	bmjopen-2020-038720.R1
Article Type:	Original research
Date Submitted by the Author:	30-Apr-2020
Complete List of Authors:	Sandhar, Sandeep; University of London St George's, Institute for Infection and Immunity<br>Smith, Toby O.; University of Oxford, Nuffield Department of Orthopaedics and Musculoskeletal Sciences<br>Toor, Kavanbir; University of London St George's, Institute for Infection and Immunity<br>Howe, Franklyn ; University of London St George's, Molecular and Clinical Sciences Research Institute<br>Sofat, Nidhi; University of London St George's, Institute for Infection and Immunity
**Primary Subject Heading**:	Rheumatology
Secondary Subject Heading:	Diagnostics
Keywords:	Knee < ORTHOPAEDIC & TRAUMA SURGERY, Hip < ORTHOPAEDIC & TRAUMA SURGERY, PAIN MANAGEMENT, RHEUMATOLOGY

SCHOLARONE™
Manuscripts

I, the Submitting Author has the right to grant and does grant on behalf of all authors of the Work (as defined in the below author licence), an exclusive licence and/or a non-exclusive licence for contributions from authors who are: i) UK Crown employees; ii) where BMJ has agreed a CC-BY licence shall apply, and/or iii) in accordance with the terms applicable for US Federal Government officers or employees acting as part of their official duties; on a worldwide, perpetual, irrevocable, royalty-free basis to BMJ Publishing Group Ltd ("BMJ") its licensees and where the relevant Journal is co-owned by BMJ to the co-owners of the Journal, to publish the Work in this journal and any other BMJ products and to exploit all rights, as set out in our licence.

The Submitting Author accepts and understands that any supply made under these terms is made by BMJ to the Submitting Author unless you are acting as an employee on behalf of your employer or a postgraduate student of an affiliated institution which is paying any applicable article publishing charge ("APC") for Open Access articles. Where the Submitting Author wishes to make the Work available on an Open Access basis (and intends to pay the relevant APC), the terms of reuse of such Open Access shall be governed by a Creative Commons licence – details of these licences and which Creative Commons licence will apply to this Work are set out in our licence referred to above.

Other than as permitted in any relevant BMJ Author's Self Archiving Policies, I confirm this Work has not been accepted for publication elsewhere, is not being considered for publication elsewhere and does not duplicate material already published. I confirm all authors consent to publication of this Work and authorise the granting of this licence.

For peer review only

Title: Risk factors for pain and functional impairment in people with knee and hip osteoarthritis: a systematic review and meta-analysis

Concise Title: Factors associated with pain and impaired function in OA

Authors: Sandeep Sandhar,[1] Toby Smith,[2,3] Franklyn Howe,[4] Kavanbir Toor,[1] Nidhi Sofat[1]

Affiliations

1. Institute for Infection and Immunity Research, St Georges, University of London, London, UK

2. Nuffield Department of Orthopaedics, Rheumatology and Musculoskeletal Sciences, University of Oxford, Oxford, UK

3. Faculty of Medicine and Health Sciences, University of East Anglia, Norwich, UK

4. Neurosciences Research Centre, St Georges, University of London, London, UK

Corresponding author: Professor Nidhi Sofat (nsofat@sgul.ac.uk), Institute for Infection and Immunity, St Georges, University of London, London, SW17 0RE

Work done on behalf of the OA Tech working research group.

Email addresses: ssandhar@sgul.ac.uk (Sandeep Sandhar), toby.smith@ndorms.ox.ac.uk (Toby Smith), howefa@sgul.ac.uk (Franklyn Howe), m1604502@sgul.ac.uk (Kavanbir Toor), nsofat@sgul.ac.uk (Nidhi Sofat)

For peer review only - http://bmjopen.bmj.com/site/about/guidelines.xhtml

ABSTRACT

Objective: To identify risk factors for pain and functional deterioration in people with knee and hip osteoarthritis (OA) to form the basis of a future 'stratification tool' for OA development or progression.

Design: Systematic review and meta-analysis

Methods: An electronic search of the literature databases: MEDLINE, EMBASE, CINAHL, MEDLINE and Web of Science (1990-February 2020) was conducted. Studies which identified risk factors for pain and functional deterioration to knee and hip OA were included. Where data and study heterogeneity permitted, meta-analyses presenting mean difference (MD) and odd ratios (OR) with corresponding 95% confidence intervals (CI) were undertaken. Where this was not possible, a narrative analysis was undertaken. The Downs & Black tool assessed methodological quality of selected studies before data extraction. Pooled analysis outcomes were assessed and reported using the GRADE approach.

Results: 82 studies (41,810 participants) were included. On meta-analysis: there was moderate quality evidence that knee OA pain was associated with factors including: Kellgren and Lawrence≥ 2 (MD:2.04, 95% CI:1.48,2.81; p<0.01), increasing age (MD:1.46, 95% CI:0.26,2.66; p=0.02) and whole-organ MRI scoring method Knee effusion score ≥1 (OR: 1.35, 95% CI: 0.99,1.83; p=0.05). On narrative analysis: knee OA pain was associated with factors including WORMS meniscal damage≥1 (OR: 1.83, 95% CI:1.23,2.71; p=0.003). Predictors of joint pain in hip OA were large acetabular bone marrow lesions (OR: 5.23), chronic widespread pain (OR: 5.02) and large hip BMLs (OR: 4.43).

For peer review only - http://bmjopen.bmj.com/site/about/guidelines.xhtml

Conclusions: Our study identified risk factors for clinical pain in OA by imaging measures that can assist in predicting and stratifying people with knee/hip OA. A 'stratification tool' combining verified risk factors that we have identified, would allow selective stratification based on pain and structural outcomes in OA.

PROSPERO Registration: CRD42018117643

ARTICLE SUMMARY: strengths and limitations of this study

- This study has been reported in accordance with the Preferred Reporting Items for Systematic Reviews and Meta-Analyses reporting checklist.
- Analyses have been undertaken respecting potential sources of know statistical heterogeneity.
- Searches included both published and unpublished sources of literature to reduce the risk of omitting potentially eligible data.
- There was a paucity of available data to permit meta-analyses of risk factors for pain and functional impairment.
- The variability in methods of assessing risk and reporting of frequency of risk characteristics limited analyses

For peer review only

For peer review only - http://bmjopen.bmj.com/site/about/guidelines.xhtml

INTRODUCTION

It has been reported that over 30.8 million US adults suffer from osteoarthritis (OA) (1). Between 1990-2010, the years lived with disability worldwide caused by OA increased from 10.5 million to 17.1 million, an increase of 62.9% (2). Current OA treatment lacks any disease-modifying treatments with a predominance to manage symptoms rather than modify underlying disease (3). The clinical symptoms of OA can be assessed using several questionnaires, the most common of which is the Western Ontario McMaster Arthritic Index (WOMAC) (4, 5, 6). Although pain is recognised as an important outcome measure in OA, it is not clear what the optimal assessment tools are in OA and how they relate to other risk factors.

OA has various subtypes and since current therapies cannot prevent OA progression, early detection and stratification of those at risk may enable effective pre-symptomatic interventions (7, 8). Several methods are used to define, diagnose and measure OA progression, including imaging techniques [e.g. plain radiography, Computed Tomography (CT) and Magnetic Resonance Imaging (MRI)]. Plain radiography provides high contrast and high resolution images for cortical and trabecular bone, but not for non-ossified structures (e.g. synovial fluid) (9). The most recognised radiographic measure classifying OA severity is Kellgren and Lawrence (KL) grading which assesses osteophytes, joint space narrowing (JSN), sclerosis and bone deformity (10, 11). However, it has been argued that MRI may be more suitable for imaging arthritic joints, providing a whole organ image of the joint (12). Whole-organ MRI scoring method (WORMS) is used in MRI for OA assessing damage, providing a detailed analysis of the joint.

Recently, OMERACT-OARSI (Outcome Measures in Rheumatology-Osteoarthritis Research Society International) have published a core domain set for clinical trials in hip and/or knee OA (13). Six domains were assessed as mandatory in the assessment of OA, including pain, physical function,

For peer review only - http://bmjopen.bmj.com/site/about/guidelines.xhtml

quality of life, patient's global assessment of the target joint, and adverse events including mortality and/or joint structure, depending on the intervention tested. However, there remains a need to identify risk factors for pain and structural damage in OA so that potential interventions can be studied in a timely manner. The purpose of this systematic review was therefore to identify risk factors for pain, worsening function and structural damage that can predict knee/hip OA development and progression. By identifying risk factors for OA pain and structural damage, tools for stratifying specific disease groups could be developed in the future.

METHODS

This systematic review has been reported in accordance with the PRISMA reporting guidelines. The review protocol was registered *a priori* through PROSPERO (Registration: CRD42018117643).

Search Strategy

A systematic search of the literature was undertaken from 1st January 1990 to 1st February 2020 using electronic databases: MEDLINE (Ovid), EMBASE (Ovid), MEDLINE, Web of Science and CINAHL (EBSCO). An example of the EMBASE search strategy of included search terms and Boolean operators is presented in **Supplementary File 1**. Unpublished literature databases including Clinicaltrials.gov, the WHO International Registry of Clinical Trials and OpenGrey were also searched.

Study Identification

Studies were eligible for inclusion if they were a full-text article that satisfied all of the following:

1) 100 or more participants analysed in the study (to increase power for comparisons);
2) convincing definition of OA using American College of Rheumatology criteria (14), based on symptoms of sustained pain and stiffness in the affected joint, radiographic changes

For peer review only - http://bmjopen.bmj.com/site/about/guidelines.xhtml

including osteophytes, cartilage loss, bone cysts/sclerosis and joint space narrowing, with normal inflammatory markers;

3) abstract/title that must refer to pain and/or structure in relation to OA as a primary disease;
4) Knee or hip OA;
5) pain and/or function scores;
6) joint imaged and
7) minimum six-month follow-up of pain/function outcome measures.

Non-English studies, letters, conference articles and reviews were excluded.

The titles and abstracts were reviewed by one reviewer (SS). The full-text for each paper was assessed for eligibility by one reviewer (SS) and double-checked by a second (TS). Any disagreements were addressed through discussion and adjudicated by a third reviewer (NS or FH). All studies which satisfied the criteria were included in the review.

<u>*Quality Assessment*</u>

To assess the risk of bias and the power of the methodology, the Downs & Black (D&B) tool was applied (15). These tools assessed the following aspects of each study: reporting quality, external validity, internal validity- bias, selection bias and power. The modified D&B tool was used. Accordingly, the 27-item randomised controlled trial (RCT) version was used for RCTs whilst the 18-item non-RCT version was used for non-RCT designs (**Supplementary File 2**). Both 18-item and 27-item tools have been demonstrated to be valid and reliable tools to assess RCT and non-RCT papers (14). Critical appraisal was performed by one reviewer (SS) and verified by a second (KT). Any disagreements were dealt with by discussion and adjudicated through a third reviewer (TS). In previous literature D&B score ranges were given corresponding quality: excellent (26-28); good (20-25); fair (15-19); and poor (<14) (14). Item 4 on the non-RCT and Item 5 from the RCT tool are scored

For peer review only - http://bmjopen.bmj.com/site/about/guidelines.xhtml

2 points, hence the total scores equate to 19 and 35 points respectively. The D&B tool was used to exclude poor quality studies with a score 15/28 or lower in RCTs and 10/19 or lower in non-RCTs.

Data Extraction

Data were extracted including: subject demographic data, study design, pain and function outcome measures, imaging used, OA severity scores, change in pain and function outcomes and change in OA severity scores. After all relevant data had been extracted, authors of these papers were approached to try and attain individual patient data (IPD) related to baseline and change in pain, function and structural scores for each study. No data was received from authors to inform this analysis.

Outcomes

The primary outcome was to determine the development of pain and functional impairment for those with knee and hip OA. The secondary outcome was to determine which factors are associated with structural changes in knee and hip OA.

Data Analysis

All data were assessed for study heterogeneity through scrutiny of the data extraction tables. These identified that there was minimum study-based heterogeneity based on: population, study design and interventions-exposure variabilities for given outcomes. Where there was study heterogeneity, a narrative analysis was undertaken. In this instance, the odds ratio (OR) of all predictor variables were tabulated with a range of OR presented. Where there was sufficient data to pool (two or more studies with data available to analyse) and study homogeneity evident, a pooled meta-analysis was deemed appropriate. As interpreted by the Cochrane Collaboration (16), when I^2 was 50% or greater representing high-statistical heterogeneity, a random-effects model meta-analysis was undertaken. When I^2 was less than this figure, a fixed effects model approach was adopted. Continuous

For peer review only - http://bmjopen.bmj.com/site/about/guidelines.xhtml

outcomes were assessed using mean difference (MD) scores of measures for developing severe OA, whereas dichotomous variables were assessed through OR data. All data were presented with 95% confidence intervals (CI) and forest-plots.

Due to the presentation of the data, there were minimal data to permit meta-analyses. Where there was insufficient data to pool the analysis (data only available from one study), a narrative analysis was undertaken to assess risk factors for the development of increased pain and functional impairment. Planned subgroup analyses included determine whether there was a difference in risk factors based on: (1) anatomical regions (i.e. difference between hip OA and knee OA); (2) geographical region. Analyses were undertaken on STATA version 14.0 (Stata Corp, Texas, USA) with forest plots constructed using RevMan Review Manager (RevMan) [Computer program]. Version 5.3. Copenhagen: The Nordic Cochrane Centre, The Cochrane Collaboration, 2014.)

RESULTS

Search Strategy

The results of the search strategy are presented in **Figure 1**. In total, 11,010 citations were identified. Of these, 141 papers were deemed potentially eligible and screened at full-text level. Of these, 82 met the selected criteria and were included.

Characteristics of Included Studies

A summary of the included studies is presented as **Table 1**. This consisted of 31 non-RCTs (27 observational cohort studies/four case-control studies) and 51 RCTs.

In total, 45,767 knees were included in the analysis. This consisted of 13,870 males and 23,497 females; four studies did not report the gender of their cohorts (17, 18, 19, 20). Thirty-six studies

For peer review only - http://bmjopen.bmj.com/site/about/guidelines.xhtml

were undertaken in the USA; 30 were undertaken in Europe; nine were conducted in Australasia and seven in Asia. Mean age of the cohorts was 61.7 years (standard deviation (SD): 7.56); 36 studies did not report age (17, 21, 22-54). Mean follow-up period was 35.4 months (SD: 33.6). The most common measures of pain were WOMAC pain (n=55; 50%) and Visual Analogue Scale (VAS) Pain (n=21; 19%). The most frequently used measures of function were WOMAC function (n=52; 44%), physical tests (n=16; 14%) and SF-36 (n=10; 9%).

Methodological Quality Assessment

The methodological quality of the evidence was moderate (**Supplementary File 2; Supplementary File 3**). Based on the results of the D&B non-RCT tool (31 studies; **Supplementary File 2**), recurrent strengths of the evidence were clear description of the participants recruited (29 studies; 94%), the representative nature that participants were to the population (31 studies; 100%), and variability in data presented for the main outcomes (31 studies; 100%). Furthermore the main outcome measures were deemed reliable and valid in all studies (31 studies; 100%) with 89% (27 studies; 87%) studies adopting appropriate statistical analyses for their datasets. Recurrent limitations were not clearly reporting the main findings (20 studies; 65%), issues regarding the representation of the cohort from the wider public (18 studies; 58%) and only six studies (19%) basing their sample sizes on an *a prior* power calculation. All the studies reviewed for the systematic review and meta-analysis are cited in references (17-99).

The results from the D&B RCT checklist (51 studies; **Supplementary File 3**) similarly reported findings with strength of the evidence around clear reporting of the cohort characteristics (49 studies; 96%) and interventions (50 studies; 98%), adoption of reliable/valid outcome measures (51 studies; 100%) and reported high compliance to study processes (37 studies; 73%). Recurrent weaknesses included recruiting cohorts which may not have been reflective of the wider population (19 studies; 37%), in

For peer review only - http://bmjopen.bmj.com/site/about/guidelines.xhtml

clinic settings which may not have represented typical clinical practice (21 studies; 41%) and poorly adjusting for potential confounders in analyses (26 studies; 51%).

Knee OA

Narrative Review

Findings from the narrative analysis found the following were predictors for worsening joint pain: KL3 or 4 in women (OR: 11.3), a WORMS lateral meniscal cyst (MC) score of 1 (OR: 4.3), presence of chronic widespread pain (CWP) (OR: 3.15), increase of ≥2 in WORMS BML score after 15 months (OR: 3.2), meniscal maceration (OR: 2.82). We also found the following were the highest predictors of worsening function in people with knee OA: KL of <3 (OR: 3.28), modified KL 3a (OR: 1.65), modified KL 4a (OR: 1.46), presence of osteophytes (OR: 1.31) and female gender (OR: 1.79 to 2.06).

Meta-Analysis

Two studies were identified where data could be evaluated for OA risk factors by meta-analysis (41, 67). Six variables significantly associated with the development of knee OA. As illustrated in **Table 2** and **Figures 2a-d**, age (MD:1.46, 95% CI: 0.26 to 2.66; p=0.02; N=823), KL of ≥ 2(MD:2.04, 95% CI: 1.48 to 2.81; p<0.01; N=823) and knee effusion score ≥1 (OR: 1.35, 95% CI: 0.99 to 1.83: p=0.05; N=823) were all associated with the development of knee OA based on moderate quality evidence. The variables baseline function score (MD:-11.50, 95% CI: -20.73 to -2.27; p=0.01; N=330), cartilage loss graded 2 or more (OR: 2.11, 95% CI: 1.18 to 3.79; p=0.01; N=493) and meniscal damage graded 1 or more (OR: 1.83, 95% CI: 1.23 to 2.71; p=0.003; N=493) were all associated with OA knee development based on lower quality evidence (**Table 2**). The variables of gender (when combining male and female), BML score, ethnicity, BMI and synovitis were not shown to be significantly associated with the knee OA development (**Table 2**).

For peer review only - http://bmjopen.bmj.com/site/about/guidelines.xhtml

Due to the limited availability of data it was not possible to conduct the planned subgroup analyses to determine whether there was a difference in risk factors based on anatomical or geographical regions.

Hip OA

Narrative Analysis

This was based on low-quality evidence. There was no association between the development of hip BML and BMI or age. Predictors for worsening joint pain for people with hip OA included a large acetabular BML (OR: 5.23), a large femoral head BML (OR: 4.42) with any large hip BML (OR: 4.43), CWP (OR: 5.02) and depression (OR: 1.90). Baseline knee pain score (MD:-1.42; 95% CI: -1.61 to -1.23; p<0.01; N=198) and baseline hip pain score (MD:-0.72; 95% CI: -0.97 to -0.47; p<0.01; N=198) were significantly associated with the development of hip BMLs and pain.

Meta-Analysis

There were insufficient data to permit meta-analysis for the hip OA dataset.

DISCUSSION

Our systematic review and meta-analysis identified risk factors for knee and hip OA pain and structural damage based on evaluation of 82 studies. For the knee, increasing pain in knee OA was associated with KL grade 3 or 4 in women, WORMS lateral MC, presence of CWP, increase of ≥2 in WORMS BML score after 15 months and meniscal maceration. In addition, KL<3, KL 3a, KL 4a, osteophyte presence and female gender were associated with worsening function in people with knee OA. On meta-analysis, age, radiological features (KL score of 2 or more) and osteophyte

For peer review only - http://bmjopen.bmj.com/site/about/guidelines.xhtml

For peer review only

presence, knee effusion, poor baseline function, cartilage loss graded 2 or more zones and meniscal tears were associated with development and/or progression of knee OA.

Our meta-analysis identified risk factors that are appreciated only when results were pooled together. These were namely: WORMS-defined knee effusion score ≥1, cartilage loss graded 2 or more, meniscal damage graded 1 or more and baseline function score. To our knowledge, this is the currently the largest and most up to date systematic review of its kind, reviewing 82 primary studies in 41,810 participants. Nonetheless, some risk factors from our meta-analysis have been recognised previously. For example, Silverwood *et al.* reported previous injuries are associated to developing knee OA, supporting the present analysis (95). Kingsbury *et al.* identified age and KL grade as predictive factors for developing knee OA, supporting the present findings (96). Therefore the meta-analyses provided both novel and supporting findings for risk factors associated with developing and progressing knee OA. A machine learning study assessed risk factors associated with pain and radiological progression in knee OA found that BMLs, osteophytes, medial meniscal extrusion, female gender and urine CTX-II contributed to progression (95). Nelson *et al's.* work is supported by other studies (95, 96). Therefore the findings of this analysis support previous findings.

After plain radiography, MRI was the most used modality with WORMS as the commonest scoring reported for MRI. The MRI Osteoarthritis Knee Score (MOAKS) (98), expanded on WORMS by scoring entire sub-regions for BMLs rather than each BML, further division of cartilage regions and refined the features assessed in meniscal morphology**.** Due to this progression from WORMS, having no MOAKS studies included in our final selection was surprising. This could be due to the eligibility criteria being too restrictive. A future systematic review and meta-analysis focusing on the imaging aspect of evaluating OA will be important. In hip OA, the evaluation of BML size and location is essential in predicting pain progression and these can be assessed effectively using MRI. We recommend that all MRI studies for hip OA evaluate BML size and location.

For peer review only - http://bmjopen.bmj.com/site/about/guidelines.xhtml

Gait analysis is considered a risk factor for pain/function and was therefore included as a target outcome measure. However, few studies included gait analysis measures, which could not be included in the analysis, perhaps due to the minimum sample size (n=100) being too restrictive.

There were several limitations within our study. Firstly, despite identifying novel risk factors for exhibiting knee OA, a small dataset was pooled together for the meta-analysis (2 studies) compared to Silverwood *et al.* (34 studies) (93). This was particularly apparent for hip OA where only 12 studies assessed this population (8,17,23,30,46,47,48,50,54,71,76,94). Consequently the small dataset influenced the GRADE assessment that determined the evidence as low to moderate, restricting the strength of the associations of risk factors with OA development and progression. Further work may impact our confidence in the estimated effect, for both studies recruiting participants with hip and knee OA. Secondly, the eligibility criteria may have been too restrictive, resulting in limited papers including gait analysis or MOAKS. Wet biomarkers were not included in our analyses. Finally, the inability to pool data was partly attributed to variability in methods to report data. Standardising data collection and reporting is important in conducting meta-analyses. We believe the following should be undertaken to improve data pooling in future work: ensuring group comparisons in studies are selected from the same population (people with confirmed OA) to improve internal validity, observational studies should conduct a power analysis to determine sample sizes and all studies should include absolute frequency of events data rather than summary odds ratios. Such considerations will improve future meta-analyses to identify OA risk factors.

To conclude, our work helps to develop steps towards building a stratification tool for risk factors for knee OA pain and structural damage development. We also highlight the need for collection of core datasets based on defined domains, that has recently also been highlighted by the OMERACT-OARSI

For peer review only - http://bmjopen.bmj.com/site/about/guidelines.xhtml

core domain set for knee and hip OA (13). Collection of future datasets based on standardised core outcomes will assist in more robust identification of risk factors for large joint OA.

Figure and Table Legends

Figure 1: PRISMA flow-chart

Figure 2a: Forest-plot to present the association between gender and presentation of knee OA.
Figure 2b: Forest-plot to present the association between age and presentation of knee OA.
Figure 2c: Forest-plot to present the association between knee effusion score greater or equal to 1 and presentation of knee OA.
Figure 2d: Forest-plot to present the association between BMI and presentation of knee OA.

Table 1: Characteristics of included studies

Supplementary File 1: Search strategy adopted for the EMBASE database search.

Supplementary File 2: Methodological appraisal results based on the Downs and Black non-RCT Checklist

Supplementary File 3: Methodological appraisal results based on the Downs and Black RCT Checklist

DECLARATIONS

Contributorship statement: SS, TS and KT conducted the information searches and primary data analysis for the study. FAH was involved in conception of the study, reviewing the results and assisting in writing the manuscript, NS conceived the study, contributed to data analysis, obtained funding and reviewed the manuscript.

Data sharing statement: Extra data sharing is available by emailing nsofat@sgul.ac.uk

Ethics: No Ethical Approval was required for this study

Patient and Public Involvement: The research team acknowledges the assistance of both the OA tech network and Engineering and Physical Sciences Research Council. The authors also acknowledge receiving assistance from a meeting that enabled a consensus to be met on the eligibility criteria to

For peer review only - http://bmjopen.bmj.com/site/about/guidelines.xhtml

be used, and this meeting consisted of the following people: Dr Angela Kedgley, Mrs Abiola Harrison, Professor Alan Boyde, Professor Alan Silman, Dr Amara Ezeonyeji, Miss Caroline Hing, Professor Cathy Holt, Ms Debbie Rolfe, Dr Enrica Papi, Ms Freija Ter Heegde, Mr Jingsong Wang, Dr John Garcia, Dr Mark Elliott, Professor Mary Sheppard, Miss Natasha Kapella, Mr Richard Rendle, Dr Shafaq Sikandar, Dr Sherif Hosny, Miss Soraia Silva, Miss Soraya Koushesh, Miss Susanna Cooper and Dr Thomas Barrick. No writing assistance was used.

Role of Funding Source: This study was funded by the Engineering and Physical Sciences Research Council (EPSRC) under the reference code 'EP/N027264/1' and The Wellcome Trust ISSF award to NS [Grant number 204809/Z/16/Z]. The funder had no input on the study design, data collection and analysis, manuscript preparation or the choice to submit it for publication.

Competing interests: None of the authors had any relation or contact with companies whose products or services may be related to the topic of the article.

REFERENCES

1. Cisternas M, Murphy L, Sacks, J, Solomon D, Pasta D and Helmick C. Alternative Methods for Defining Osteoarthritis and the Impact on Estimating Prevalence in a US Population-Based Survey. Arthritis Care & Research 2016; 68:574-580.
2. Cross M, Smith E, Hoy D, Nolte S, Ackerman I, Fransen M, Bridgett L, et al. The global burden of hip and knee osteoarthritis: estimates from the Global Burden of Disease 2010 study. Annals of the Rheumatic Diseases 2014; 73:1323-1330.
3. Wu Y, Goh E, Wang D and Ma S. Novel treatments for osteoarthritis: a recent update. Open Access Rheumatology: Research and Reviews 2018; 10:135-140.

For peer review only - http://bmjopen.bmj.com/site/about/guidelines.xhtml

4. Bellamy N, Buchanan WW, Goldsmith CH, Campbell J and Stitt LW. Validation study of WOMAC: a health status instrument for measuring clinically important patient relevant outcomes to antirheumatic drug therapy in patients with osteoarthritis of the hip or knee. J Rheumatol 1988; 15:1833-40.

5. Kraus V, Blanco F, Englund M, Karsdal M and Lohmander L. Call for standardized definitions of osteoarthritis and risk stratification for clinical trials and clinical use. Osteoarthritis and Cartilage 2015;23:1233-1241

6. Jin X, Jones G, Cicuttini F, Wluka A, Zhu Z, Han W, et al. Effect of Vitamin D Supplementation on Tibial Cartilage Volume and Knee Pain Among Patients With Symptomatic Knee Osteoarthritis: A Randomized Clinical Trial. JAMA 2016; 315:1005-13.

7. Hill CL, March LM, Aitken D, Lester SE, Battersby R, Hynes K, et al. Fish oil in knee osteoarthritis: a randomised clinical trial of low dose versus high dose. Ann Rheum Dis 2016; 75:23-9.

8. Maheu E, Cadet C, Marty M, Moyse D, Kerloch I, Coste P, et al. Randomised, controlled trial of avocado-soybean unsaponifiable (Piascledine) effect on structure modification in hip osteoarthritis: the ERADIAS study. Ann.Rheum.Dis 2014;73:376-84.

9. Peterfy C. Imaging Techniques. J Klippel, P Dieppe (Eds.), Rheumatology 2E, 1, Mosby, Philadelphia 1998; 1:14.1-14.18

10. Kellgren J and Lawrence J. Radiological Assessment of Osteo-Arthrosis. Annals of the Rheumatic Disease 1957; 16:494-502.

11. Schiphof D, Boers M and Bierma-Zeinstra SM. Differences in descriptions of Kellgren and Lawrence grades of knee osteoarthritis. Annals of the Rheumatic Disease 2008; 67:1034–1036.

For peer review only - http://bmjopen.bmj.com/site/about/guidelines.xhtml

12. Peterfy C, Guermazi A, Zaim S, Tirman P Miaux Y, White D, et al. Whole-Organ Magnetic Resonance Imaging Score (WORMS) of the knee in osteoarthritis. Osteoarthritis and Cartilage 2004;12:177-190.

13. Smith TO, Hawker GA, Hunter DJ. et al. The OMERACT-OARSI core domain set for measurement in clinical trials of hip and/or knee osteoarthritis. The Journal of Rheumatology 2019, 46(8): 981-989

14. Altman R, Asch E, Bloch D, Bole G, Borenstein D, Brandt K, et al. The American College of Rheumatology criteria for the classification and reporting of osteoarthritis of the knee. Arthritis Rheum 1986; 29: 1039-1049

15. Downs S and Black N. The feasibility of creating a checklist for the assessment of the methodological quality both of randomised and non-randomised studies of health care interventions. Journal of Epidemiology & Community Health 1998; 52:377-384.

16. Deeks JJ. Higgins JPT, Altman DG on behalf of the Cochrane Statistical Methods Group. Chapter 9: Analysing data and undertaking meta-analyses. In: Cochrane Handbook for Systematic Reviews of Interventions. Version 5.1.0 [update March 2011] Ed: Higgins JPT, Green S. The Cochrane Collaboration. Accessed: 02 April 2019. Available at: http://handbook-5-1.cochrane.org/

17. Valdes AM, Doherty SA, Zhang W, Muir KR, Maciewicz RA, Doherty M. Inverse relationship between preoperative radiographic severity and postoperative pain in patients with osteoarthritis who have undergone total joint arthroplasty. Seminars in Arthritis & Rheumatism 2012; 41:568-75.

18. Kinds MB, Marijnissen ACA, Vincken KL, Viergever MA, Drossaers-Bakker KW, Bijlsma JWJ, et al. Evaluation of separate quantitative radiographic features adds to the prediction of incident radiographic osteoarthritis in individuals with recent onset of knee pain: 5-year follow-up in the CHECK cohort. 2012; 20:548-56.

For peer review only - http://bmjopen.bmj.com/site/about/guidelines.xhtml

19. Davis J, Eaton CB, Lo GH, Lu B, Price LL, McAlindon TE, et al. Knee symptoms among adults at risk for accelerated knee osteoarthritis: data from the Osteoarthritis Initiative. Clin.Rheumatol 2017;36:1083-9.

20. Akelman MR, Fadale PD, Hulstyn MJ, Shalvoy RM, Garcia A, Chin KE, et al. Effect of Matching or Overconstraining Knee Laxity During Anterior Cruciate Ligament Reconstruction on Knee Osteoarthritis and Clinical Outcomes: A Randomized Controlled Trial With 84-Month Follow-up. Am.J.Sports Med 2016; 44:1660-70.

21. Yu SP, Williams M, Eyles JP, Chen JS, Makovey J, Hunter DJ. Effectiveness of knee bracing in osteoarthritis: pragmatic trial in a multidisciplinary clinic. Int.J.Rheum.Dis. 2016;19:279-86.

22. Urish KL, Keffalas MG, Durkin JR, Miller DJ, Chu CR, Mosher TJ. T2 texture index of cartilage can predict early symptomatic OA progression: data from the osteoarthritis initiative. Osteoarthritis & Cartilage 2013; 21:1550-7.

23. Rozendaal RM, Koes BW, van Osch GJ, Uitterlinden EJ, Garling EH, Willemsen SP, et al. Effect of glucosamine sulfate on hip osteoarthritis: a randomized trial. Ann.Intern.Med 2008; 148:268-77.

24. Roman-Blas JA, Castaneda S, Sanchez-Pernaute O, Largo R, Herrero-Beaumont G, CS/GS Combined Therapy Study Group. Combined Treatment With Chondroitin Sulfate and Glucosamine Sulfate Shows No Superiority Over Placebo for Reduction of Joint Pain and Functional Impairment in Patients With Knee Osteoarthritis: A Six-Month Multicenter, Randomized, Double-Blind, Placebo-Controlled Clinical Trial. Arthritis Rheumatol 2017; 69:77-85.

25. Riddle DL and Jiranek WA. Knee osteoarthritis radiographic progression and associations with pain and function prior to knee arthroplasty: a multicenter comparative cohort study. Osteoarthritis and Cartilage 2015; 23:391-6

For peer review only

26. Reginster JY, Deroisy R, Rovati LC, Lee RL, Lejeune E, Bruyere O, et al. Long-term effects of glucosamine sulphate on osteoarthritis progression: a randomised, placebo-controlled clinical trial. Lancet 2001;357 North American Edition:251-6.

27. Raynauld JP, Martel-Pelletier J, Haraoui B, Choquette D, Dorais M, Wildi LM, et al. Risk factors predictive of joint replacement in a 2-year multicentre clinical trial in knee osteoarthritis using MRI: results from over 6 years of observation. Ann.Rheum.Dis 2011; 70:1382-8.

28. Podsiadlo P., Cicuttini F.M., Wolski M., Stachowiak G.W., Wluka AE. Trabecular bone texture detected by plain radiography is associated with an increased risk of knee replacement in patients with osteoarthritis: A 6 year prospective follow up study. Osteoarthritis and Cartilage 2014; 22:71-5.

29. Pham T, Le Henanff A, Ravaud P, Dieppe P, Paolozzi L, Dougados M. Evaluation of the symptomatic and structural efficacy of a new hyaluronic acid compound, NRD101, in comparison with diacerein and placebo in a 1 year randomised controlled study in symptomatic knee osteoarthritis. Ann.Rheum.Dis 2004; 63:1611-7.

30. Pavelka K., Gatterova J., Gollerova V., Urbanova Z., Sedlackova M., Altman RD. A 5-year randomized controlled, double-blind study of glycosaminoglycan polysulphuric acid complex (Rumalon) as a structure modifying therapy in osteoarthritis of the hip and knee. Osteoarthritis and Cartilage 2000; 8:335-42.

31. Michel BA, Stucki G, Frey D, De Vathaire F, Vignon E, Bruehlmann P, et al. Chondroitins 4 and 6 sulfate in osteoarthritis of the knee: a randomized, controlled trial. Arthritis Rheum 2005; 52:779-86.

32. Messier SP, Loeser RF, Miller GD, Morgan TM, Rejeski WJ, Sevick MA, et al. Exercise and dietary weight loss in overweight and obese older adults with knee osteoarthritis: the Arthritis, Diet, and Activity Promotion Trial. Arthritis Rheum 2004; 50:1501-10.

For peer review only - http://bmjopen.bmj.com/site/about/guidelines.xhtml

33. McAlindon T, LaValley M, Schneider E, Nuite M, Lee JY, Price LL, et al. Effect of vitamin D supplementation on progression of knee pain and cartilage volume loss in patients with symptomatic osteoarthritis: a randomized controlled trial. JAMA 2013;309:155-62.
34. Marsh JD, Birmingham TB, Giffin JR, Isaranuwatchai W, Hoch JS, Feagan BG, et al. Cost-effectiveness analysis of arthroscopic surgery compared with non-operative management for osteoarthritis of the knee. BMJ Open 2016; 6:e009949,2015-009949.
35. Lohmander LS, Hellot S, Dreher D, Krantz EF, Kruger DS, Guermazi A, et al. Intraarticular sprifermin (recombinant human fibroblast growth factor 18) in knee osteoarthritis: a randomized, double-blind, placebo-controlled trial. Arthritis Rheumatol 2014; 66:1820-31.
36. Kongtharvonskul J, Woratanarat P, McEvoy M, Attia J, Wongsak S, Kawinwonggowit V, et al. Efficacy of glucosamine plus diacerein versus monotherapy of glucosamine: a double-blind, parallel randomized clinical trial. Arthritis Res.Ther 2016; 18:233,016-1124-9.
37. Katz JN, Brophy RH, Chaisson CE, de Chaves L, Cole BJ, Dahm DL, et al. Surgery versus physical therapy for a meniscal tear and osteoarthritis. N.Engl.J.Med 2013;368:1675-84.
38. Karsdal MA, Byrjalsen I, Alexandersen P, Bihlet A, Andersen JR, Riis BJ, et al. Treatment of symptomatic knee osteoarthritis with oral salmon calcitonin: results from two phase 3 trials. Osteoarthritis and Cartilage 2015; 23:532-43.
39. Housman L, Arden N, Schnitzer TJ, Birbara C, Conrozier T, Skrepnik N, et al. Intra-articular hylastan versus steroid for knee osteoarthritis. Knee Surg.Sports Traumatol. Arthrosc 2014;22:1684-92.
40. Henriksen M., Hunter D.J., Dam E.B., Messier S.P., Andriacchi T.P., Lohmander L.S., et al. Is increased joint loading detrimental to obese patients with knee osteoarthritis? A secondary data analysis from a randomized trial. Osteoarthritis and Cartilage 2013; 21:1865-75.
41. Guermazi A., Hayashi D., Roemer F.W., Niu J., Yang M., Lynch J.A., et al. Cyst-like lesions of the knee joint and their relation to incident knee pain and development of radiographic osteoarthritis: The MOST study. Osteoarthritis and Cartilage 2010; 18:1386-92.

For peer review only - http://bmjopen.bmj.com/site/about/guidelines.xhtml

42. Glass NA, Torner JC, Frey Law LA, Wang K, Yang T, Nevitt MC, et al. The relationship between quadriceps muscle weakness and worsening of knee pain in the MOST cohort: a 5-year longitudinal study. Osteoarthritis and Cartilage 2013; 21:1154-9.

43. Filardo G, Di Matteo B, Di Martino A, Merli ML, Cenacchi A, Fornasari P, et al. Platelet-Rich Plasma Intra-articular Knee Injections Show No Superiority Versus Viscosupplementation: A Randomized Controlled Trial. Am.J.Sports Med 2015;43:1575-82.

44. Ettinger WH,Jr, Burns R, Messier SP, Applegate W, Rejeski WJ, Morgan T, et al. A randomized trial comparing aerobic exercise and resistance exercise with a health education program in older adults with knee osteoarthritis. The Fitness Arthritis and Seniors Trial (FAST). JAMA 1997; 277:25-31.

45. Eckstein F, Hitzl W, Duryea J, Kent Kwoh C, Wirth W, OAI investigators. Baseline and longitudinal change in isometric muscle strength prior to radiographic progression in osteoarthritic and pre-osteoarthritic knees--data from the Osteoarthritis Initiative. Arthritis & rheumatology 2013; 21:682-90.

46. Dougados M, Nguyen M, Berdah L, Mazieres B, Vignon E, Lequesne M, et al. Evaluation of the structure-modifying effects of diacerein in hip osteoarthritis: ECHODIAH, a three-year, placebo-controlled trial. Evaluation of the Chondromodulating Effect of Diacerein in OA of the Hip. Arthritis Rheum 2001; 44:2539-47

47. Chandrasekaran S, Gui C, Darwish N, Lodhia P, Suarez-Ahedo C, Domb BG. Outcomes of Hip Arthroscopic Surgery in Patients With Tonnis Grade 1 Osteoarthritis With a Minimum 2-Year Follow-up: Evaluation Using a Matched-Pair Analysis With a Control Group With Tonnis Grade 0. Am.J.Sports Med 2016;44:1781-8.

48. Chandrasekaran S, Darwish N, Gui C, Lodhia P, Suarez-Ahedo C, Domb BG. Outcomes of Hip Arthroscopy in Patients with Tonnis Grade-2 Osteoarthritis at a Mean 2-Year Follow-up: Evaluation Using a Matched-Pair Analysis with Tonnis Grade-0 and Grade-1 Cohorts. Journal of Bone & Joint Surgery 2016; 98:973-82.

For peer review only - http://bmjopen.bmj.com/site/about/guidelines.xhtml

49. Campbell DG, Duncan WW, Ashworth M, Mintz A, Stirling J, Wakefield L, et al. Patellar resurfacing in total knee replacement: a ten-year randomised prospective trial. J.Bone Joint Surg.Br 2006;88:734-9.

50. Brown MT, Murphy FT, Radin DM, Davignon I, Smith MD, West CR. Tanezumab reduces osteoarthritic hip pain: results of a randomized, double-blind, placebo-controlled phase III trial. Arthritis Rheum 2013; 65:1795-803.

51. Brown MT, Murphy FT, Radin DM, Davignon I, Smith MD, West CR. Tanezumab reduces osteoarthritic knee pain: results of a randomized, double-blind, placebo-controlled phase III trial. J.Pain 2012; 13:790-8.

52. Bisicchia S, Bernardi G, Tudisco C. HYADD 4 versus methylprednisolone acetate in symptomatic knee osteoarthritis: a single-centre single blind prospective randomised controlled clinical study with 1-year follow-up. Clin.Exp.Rheumatol 2016;34:857-63.

53. Bingham CO,3rd, Buckland-Wright JC, Garnero P, Cohen SB, Dougados M, Adami S, et al. Risedronate decreases biochemical markers of cartilage degradation but does not decrease symptoms or slow radiographic progression in patients with medial compartment osteoarthritis of the knee: results of the two-year multinational knee osteoarthritis structural arthritis study. Arthritis Rheum 2006; 54:3494-507.

54. Ahedi H, Aitken D, Blizzard L, Cicuttini F, Jones G. A population-based study of the association between hip bone marrow lesions, high cartilage signal, and hip and knee pain. Clin.Rheumatol 2014;33:369-76.

55. Amin S, Guermazi A, Lavalley MP, Niu J, Clancy M, Hunter DJ, et al. Complete anterior cruciate ligament tear and the risk for cartilage loss and progression of symptoms in men and women with knee osteoarthritis. Osteoarthritis Cartilage 2008; 16:897-902

56. Antony B, Driban JB, Price LL, Lo GH, Ward RJ, Nevitt M, et al. The relationship between meniscal pathology and osteoarthritis depends on the type of meniscal damage visible on

For peer review only

For peer review only - http://bmjopen.bmj.com/site/about/guidelines.xhtml

magnetic resonance images: data from the Osteoarthritis Initiative. Osteoarthritis Cartilage 2017; 25:76-84.

57. Arden NK, Cro S, Sheard S, Dore CJ, Bara A, Tebbs SA, et al. The effect of vitamin D supplementation on knee osteoarthritis, the VIDEO study: a randomised controlled trial. Osteoarthritis Cartilage 2016; 24:1858-66.

58. Ayral X, Mackillop N, Genant HK, Kirkpatrick J, Beaulieu A, Pippingskiold P, et al. Arthroscopic evaluation of potential structure-modifying drug in osteoarthritis of the knee. A multicenter, randomized, double-blind comparison of tenidap sodium vs piroxicam. Osteoarthritis Cartilage 2003;11:198-207.

59. Baselga Garcia-Escudero J and Miguel Hernandez Trillos P. Treatment of Osteoarthritis of the Knee with a Combination of Autologous Conditioned Serum and Physiotherapy: A Two-Year Observational Study. 2015;10:e0145551.

60. Bevers K, Vriezekolk JE, Bijlsma JWJ, van den Ende,Cornelia H M., den Broeder AA. Ultrasonographic predictors for clinical and radiological progression in knee osteoarthritis after 2 years of follow-up. Rheumatology 2015;54:2000-3.

61. Birmingham T.B., Giffin J.R., Chesworth B.M., Bryant D.M., Litchfield R.B., Willits K., et al. Medial opening wedge high tibial osteotomy: A prospective cohort study of gait, radiographic, and patient-reported outcomes. Arthritis Care and Research 2009;61:648-57.

62. Brandt KD, Mazzuca SA, Katz BP, Lane KA, Buckwalter KA, Yocum DE, et al. Effects of doxycycline on progression of osteoarthritis: results of a randomized, placebo-controlled, double-blind trial. Arthritis Rheum 2005;52:2015-25.

63. Bruyere O, Pavelka K, Rovati LC, Deroisy R, Olejarova M, Gatterova J, et al. Glucosamine sulfate reduces osteoarthritis progression in postmenopausal women with knee osteoarthritis: evidence from two 3-year studies. Menopause 2004;11:138-43.

64. Conrozier T, Eymard F, Afif N, Balblanc JC, Legre-Boyer V, Chevalier X, et al. Safety and efficacy of intra-articular injections of a combination of hyaluronic acid and mannitol

For peer review only - http://bmjopen.bmj.com/site/about/guidelines.xhtml

(HAnOX-M) in patients with symptomatic knee osteoarthritis: Results of a double-blind, controlled, multicenter, randomized trial. Knee 2016; 23:842-8.

65. Dowsey M.M., Nikpour M., Dieppe P., Choong PFM. Associations between pre-operative radiographic changes and outcomes after total knee joint replacement for osteoarthritis. Osteoarthritis and Cartilage 2012; 20:1095-102.
66. Felson DT, Niu J, Yang T, Torner J, Lewis CE, Aliabadi P, et al. Physical activity, alignment and knee osteoarthritis: data from MOST and the OAI. Osteoarthritis and Cartilage 2013; 21:789-95.
67. Felson DT, Niu J, Guermazi A, Roemer F, Aliabadi P, Clancy M, et al. Correlation of the development of knee pain with enlarging bone marrow lesions on magnetic resonance imaging. Arthritis Rheum 2007; 56:2986-92.
68. Hamilton TW, Pandit HG, Maurer DG, Ostlere SJ, Jenkins C, Mellon SJ, et al. Anterior knee pain and evidence of osteoarthritis of the patellofemoral joint should not be considered contraindications to mobile-bearing unicompartmental knee arthroplasty: a 15-year follow-up. Bone & Joint Journal 2017; 99-B:632-9.
69. Hellio le Graverand MP, Clemmer RS, Redifer P, Brunell RM, Hayes CW, Brandt KD, et al. A 2-year randomised, double-blind, placebo-controlled, multicentre study of oral selective iNOS inhibitor, cindunistat (SD-6010), in patients with symptomatic osteoarthritis of the knee. Ann.Rheum.Dis 2013; 72:187-95.
70. Hochberg MC, Martel-Pelletier J, Monfort J, Moller I, Castillo JR, Arden N, et al. Combined chondroitin sulfate and glucosamine for painful knee osteoarthritis: a multicentre, randomised, double-blind, non-inferiority trial versus celecoxib. Ann.Rheum.Dis 2016; 75:37-44.
71. Hoeksma HL, Dekker J, Ronday HK, Heering A, van der Lubbe N, Vel C, et al. Comparison of manual therapy and exercise therapy in osteoarthritis of the hip: a randomized clinical trial. Arthritis Rheum 2004; 51:722-9.

For peer review only - http://bmjopen.bmj.com/site/about/guidelines.xhtml

72. Huang MH, Lin YS, Yang RC, Lee CL. A comparison of various therapeutic exercises on the functional status of patients with knee osteoarthritis. Semin.Arthritis Rheum 2003; 32:398-406.

73. Huizinga MR, Gorter J, Demmer A, Bierma-Zeinstra SMA, Brouwer RW. Progression of medial compartmental osteoarthritis 2-8 years after lateral closing-wedge high tibial osteotomy. Knee Surgery, Sports Traumatology, Arthroscopy 2017; 25:3679-86.

74. Kahn T.L., Soheili A., Schwarzkopf R. Outcomes of Total Knee Arthroplasty in Relation to Preoperative Patient-Reported and Radiographic Measures: Data From the Osteoarthritis Initiative. Geriatric Orthopaedic Surgery and Rehabilitation 2013; 4:117-26.

75. Kim YH, Park JW, Kim JS. The Clinical Outcome of Computer-Navigated Compared with Conventional Knee Arthroplasty in the Same Patients: A Prospective, Randomized, Double-Blind, Long-Term Study. J.Bone Joint Surg.Am 2017; 99:989-96.

76. Lequesne M, Maheu E, Cadet C, Dreiser R. Structural effect of avocado/soybean unsaponifiables on joint space loss in osteoarthritis of the hip. Arthritis & Rheumatism: Arthritis Care & Research 2002; 47:50-8

77. Messier SP, Gutekunst DJ, Davis C, DeVita P. Weight loss reduces knee-joint loads in overweight and obese older adults with knee osteoarthritis. Arthritis Rheum 2005; 52:2026-32.

78. Messier SP, Mihalko SL, Legault C, Miller GD, Nicklas BJ, DeVita P, et al. Effects of intensive diet and exercise on knee joint loads, inflammation, and clinical outcomes among overweight and obese adults with knee osteoarthritis: the IDEA randomized clinical trial. JAMA 2013;310: 1263-73.

79. Muraki S., Akune T., Nagata K., Ishimoto Y., Yoshida M., Tokimura F., et al. Association of knee osteoarthritis with onset and resolution of pain and physical functional disability: The ROAD study. Modern Rheumatology 2014; 24:966-73.

For peer review only

80. Muraki S, Akune T, Nagata K, Ishimoto Y, Yoshida M, Tokimura F, et al. Does osteophytosis at the knee predict health-related quality of life decline? A 3-year follow-up of the ROAD study. Clin.Rheumatol 2015; 34:1589-97.

81. Pavelka K, Gatterova J, Olejarova M, Machacek S, Giacovelli G, Rovati LC. Glucosamine sulfate use and delay of progression of knee osteoarthritis: a 3-year, randomized, placebo-controlled, double-blind study. Arch.Intern.Med 2002; 162:2113-23.

82. Rat A, Baumann C, Guillemin F. National, multicentre, prospective study of quality of life in patients with osteoarthritis of the knee treated with hylane G-F 20. Clin.Rheumatol 2011; 30:1285-93.

83. Reginster JY, Badurski J, Bellamy N, Bensen W, Chapurlat R, Chevalier X, et al. Efficacy and safety of strontium ranelate in the treatment of knee osteoarthritis: results of a double-blind, randomised placebo-controlled trial. Ann.Rheum.Dis 2013; 72:179-86.

84. Romagnoli S. and Marullo M. Mid-Term Clinical, Functional, and Radiographic Outcomes of 105 Gender-Specific Patellofemoral Arthroplasties, With or Without the Association of Medial Unicompartmental Knee Arthroplasty. J.Arthroplasty 2017; 33:688-695

85. Sanchez-Ramirez DC, van der Leeden M, van der Esch M, Roorda LD, Verschueren S, van Dieen J, et al. Increased knee muscle strength is associated with decreased activity limitations in established knee osteoarthritis: Two-year follow-up study in the Amsterdam osteoarthritis cohort. J.Rehabil.Med 2015; 47:647-54.

86. Sawitzke AD, Shi H, Finco MF, Dunlop DD, Harris CL, Singer NG, et al. Clinical efficacy and safety of glucosamine, chondroitin sulphate, their combination, celecoxib or placebo taken to treat osteoarthritis of the knee: 2-year results from GAIT. Ann.Rheum.Dis 2010; 69:1459-64.

87. Skou ST, Wise BL, Lewis CE, Felson D, Nevitt M, Segal NA, et al. Muscle strength, physical performance and physical activity as predictors of future knee replacement: a prospective cohort study. Osteoarthritis & Cartilage 2016; 24:1350-6.

For peer review only - http://bmjopen.bmj.com/site/about/guidelines.xhtml

88. Sowers M, Karvonen-Gutierrez CA, Jacobson JA, Jiang Y, Yosef M. Associations of anatomical measures from MRI with radiographically defined knee osteoarthritis score, pain, and physical functioning. Journal of Bone & Joint Surgery 2011; 93:241-51.

89. Spector TD, Conaghan PG, Buckland-Wright JC, Garnero P, Cline GA, Beary JF, et al. Effect of risedronate on joint structure and symptoms of knee osteoarthritis: results of the BRISK randomized, controlled trial [ISRCTN01928173. Arthritis Res.Ther 2005; 7:R625-33.

90. Sun SF, Hsu CW, Lin HS, Liou IH, Chen YH, Hung CL. Comparison of Single Intra-Articular Injection of Novel Hyaluronan (HYA-JOINT Plus) with Synvisc-One for Knee Osteoarthritis: A Randomized, Controlled, Double-Blind Trial of Efficacy and Safety. J.Bone Joint Surg.Am 2017; 99:462-71.

91. Weng MC, Lee CL, Chen CH, Hsu JJ, Lee WD, Huang MH, et al. Effects of different stretching techniques on the outcomes of isokinetic exercise in patients with knee osteoarthritis. Kaohsiung J.Med.Sci 2009; 25:306-15.

92. White DK, Neogi T, Nguyen UDT, Niu J, Zhang Y. Trajectories of functional decline in knee osteoarthritis: the Osteoarthritis Initiative. Rheumatology 2016; 55:801-8.

93. Witt C, Brinkhaus B, Jena S, Linde K, Streng A, Wagenpfeil S, et al. Acupuncture in patients with osteoarthritis of the knee: a randomised trial. Lancet 2005; 366:136-43.

94. Yusuf E, Bijsterbosch J, Slagboom PE, Kroon HM, Rosendaal FR, Huizinga TWJ, et al. Association between several clinical and radiological determinants with long-term clinical progression and good prognosis of lower limb osteoarthritis. PLoS ONE 2011; 6:e25426.

95. Silverwood V, Blagojevic-Bucknall M, Jinks C, Jordan J, Protheroe J and Jordan K. Current evidence on risk factors for knee osteoarthritis in older adults: a systematic review and meta-analysis. Osteoarthritis and Cartilage 2015; 23:507-515.

96. Kingsbury S, Corp N, Watt F, Felson D, O'Neill T, Holt C, et al. Harmonising data collection from osteoarthritis studies to enable stratification: recommendations on core data collection from an Arthritis Research UK clinical studies group. Rheumatology 2016; 55:1394-1402.

For peer review only - http://bmjopen.bmj.com/site/about/guidelines.xhtml

For peer review only

97. Nelson A, Fang F, Arbeeva L, Cleveland, R, Schwartz T, Callahan L et al. A machine learning approach to knee osteoarthritis phenotyping: data from the FNIH Biomarkers Consortium. Osteoarthritis and Cartilage 2019; 27:994-1001.

98. Hunter D, Guermazi A, Lo G, Grainger A, Conaghan P, Boudreau R, et al. Evolution of semi-quantitative whole joint assessment of knee OA: MOAKS (MRI Osteoarthritis Knee Score). Osteoarthritis and Cartilage 2011; 19:990-1002.

99. Van der Esch M, van der Leeden M, Roorda LD, Lems WF, Dekker J. Predictors of self-reported knee instability among patients with knee osteoarthritis: results of the Amsrerdam osteoarthritis cohort. Clin Rheumatol. 2016; 35(12): 3007-13

For peer review only - http://bmjopen.bmj.com/site/about/guidelines.xhtml

Table 1: Characteristics of included studies

	Study Design	Number joints (hip/knees)	Gender (male:female)	Country origin	Mean age (years)	Follow-up duration (months)	Pain outcome measures	Functional outcome measures
Ahedi 2014 (54)	Observational cohort	198 hips	111:87	Australia	UTD	132	WOMAC Pain	NA
Akelman 2016 (20)	RCT	107 knee	UTD	USA	23.5	84	KOOS pain; SF-36 Body pain	SF-36 Physical; AP laxity; IKDC2000
Amin 2008 (55)	Observational cohort	265 knees	152:113	USA	67	30	VAS Pain	WOMAC Function
Antony 2017 (56)	Observational cohort	463 knees	245:218	USA	63	24	WOMAC Pain	NA
Arden 2016 (57)	RCT	474 knees	185:289	UK	64	36	WOMAC Pain	WOMAC Function
Ayral 2003 (58)	RCT	665 knees	259:406	Australia, Belgium, Canada, Denmark, Finland, France, Hungary, Norway, Spain, United Kingdom U.S.A.	61.3	12	WOMAC Pain	WOMAC Function
Baselga Garcia-Escudero 2015 (59)	Observational cohort	118 knees	43:75	Spain	59.1	24	NRS; WOMAC Pain	WOMAC Function
Bevers 2015 (60)	Observational cohort	125 knees	57:68	Netherlands	57	24	WOMAC Pain	WOMAC Function
Bingham 2006 (53)	RCT	2483 knees	735:1748	USA Canada Austria Czech Republic France Germany Hungary Ireland Italy Netherlands Poland Croatia	UTD	24	WOMAC Pain	WOMAC Function
Birmingham 2009 (61)	Observational cohort	126 knees	100:26	Canada	47.5	24	KOOS Pain	KOOS Function; SF-36

For peer review only - http://bmjopen.bmj.com/site/about/guidelines.xhtml

								Physical; LEFS
Bisicchia 2016 (52)	RCT	150 knees	47:103	Italy	UTD	12	VAS Pain; SF-36	SF-36
Brandt 2005 (62)	RCT	431 knees	0:431	USA	54.9	30	WOMAC Pain; VAS Pain	WOMAC Function
Brown 2012 (51)	RCT	690 knees	270:420	USA	UTD	32 weeks	WOMAC Pain; NRS weekly pain	WOMAC Function; SF-36 Function
Brown 2013 (50)	RCT	621 hips	237:384	USA	UTD	32 weeks	WOMAC Pain	WOMAC Function
Bruyere 2004 (63)	RCT	319 knee	0:319	Belgium	64.0	36	WOMAC Pain	WOMAC Function
Campbell 2006 (49)	RCT	100 knees	28:72	Australia	UTD	120	American Knee Society Score; WOMAC Pain	American Knee Society Score (function); WOMAC Function
Chandrasekaran 2016A (48)	Case-Control	111 hips	66:45	USA	UTD	24	Modified Harris Hip Score; Nonarthritic hip score; VAS Pin	Modified Harris Hip Score; Nonarthritic hip score; Hip Outcome Score; Sports & ADLs
Chandrasekaran 2016B (47)	Case-Control	186 hips	96:90	USA	UTD	24	Modified Harris Hip Score; Nonarthritic hip score; VAS Pin	Modified Harris Hip Score; Nonarthritic hip score; Hip Outcome Score; Sports & ADLs
Conrozier 2016 (64)	RCT	205 knees	88:117	France	65	26	WOMAC Pain; NRS walking pain	WOMAC Function
Davis 2017 (19)	Case-control	3132 knees	UTD	USA	UTD	48	WOMAC Pain; KOOS Pain	WOMAC Function

For peer review only

For peer review only - http://bmjopen.bmj.com/site/about/guidelines.xhtml

Dougados 2001 (46)	RCT	507 hips	202:305	France	UTD	36	VAS Pain	Lequesne Index
Dowsey 2012 (65)	Observational cohort	478 knees	147:331	Australia	70.8	24	IKSS Pain	IKSS Function
Eckstein 2013 (45)	RCT	1412 knees	611:801	Austria	UTD	48	WOMAC Pain	NA
Ettinger 1997 (44)	RCT	439 knees	131:308	USA	UTD	18	Pain intensity score	Physical Test
Felson 2013 (66)	Observational cohort	3498 knees	867:1206	USA	61.2	30	WOMAC Pain	PASE
Felson 2007 (67)	Observational cohort	330 knees	111:2111	USA	62.1	15	NA	Quadriceps strength (N)
Filardo 2015 (43)	RCT	183 knees	112:71	Italy	UTD	48	KOOS Pain; IKDC	KOOS Function; Tegner; IKDC
Glass 2013 (42)	Observational cohort	4648 knees	918:1486	USA	UTD	24	WOMAC Pain; NRS Pain	WOMAC Function
Guermazi 2010 (41)	Case-control	493 knees	185:308	USA	UTD	60	WOMAC Pain	PASE
Hamilton 2017 (68)	Observational cohort	805 knees	416:289	UK	66	30	WOMAC Pain	WOMAC Function
Hellio le Graverand 2013 (69)	RCT	1457 knees	343:1114	USA Canada Australia, Belgium, Czech Republic, Germany, Hungary, Italy, Poland, Russian Federation, Slovakia, Spain, Argentina Peru	61.0	180	Oxford Knee Score	Oxford Knee Score; American Knee Society Score; Tegner
Henriksen 2013 (40)	RCT	157 knees	28:129	Denmark	UTD	24	WOMAC Pain	WOMAC Function
Hill 2016 (5)	RCT	202 knees	102:100	Australia	61	12	KOO Pain	KOOS Function and kinematic assessment
Hochberg 2016 (70)	RCT	522 knees	84:438	France Germany	62.7	24	WOMAC Pain	WOMAC Function

For peer review only - http://bmjopen.bmj.com/site/about/guidelines.xhtml

				Poland<br>Spain				
Hoeksma 2004 (71)	RCT	109 hips	33:76	Netherlands	72	6	WOMAC Pain; Huskisson's VAS; EQ-5D Pain	WOMAC Function; EQ-5D Function
Housman 2014 (39)	RCT	391 knees	130:261	USA<br>Canada<br>France<br>UK<br>Germany	UTD	6	SF-36 Body Pain; Harris Hip Score; VAS Pain	SF-36 Function; Harris Hip Score; ROM
Huang 2003 (72)	RCT	264 knees	39:93	Taiwan	62	6	WOMAC Pain	NA
Huizinga 2017 (73)	Observational cohort	298 knees	201:97	Netherlands	51	12	VAS Pain	Lequesne index; Walking speed
Jin 2016 (6)	RCT	413 knees	205:208	Australia	63.2	24	WOMAC Pain; VAS Pain	WOMAC Function
Kahn 2013 (74)	Observational cohort	174 knees	70:102	USA	67.0	6	WOMAC Pain	WOMAC Function
Karsdal 2015 (38)	RCT	2207 knees	773:1424	Denmark	UTD	24	WOMAC Pain	WOMAC Function
Katz 2013 (37)	RCT	330 knees	143:187	USA	UTD	12	KOO Pain	WOMAC Function; SF-36 Function
Kim 2017 (75)	RCT	352 knees	9:153	Republic of Korea	68.1	144	WOMAC	Knee Society Knee Score Function; ROM; UCLA Activity
Kinds 2012 (18)	RCT	565 knees	UTD	Netherlands	UTD	60	WOMAC Pain	WOMAC Function
Kongtharvonskul 2016 (36)	RCT	148 knees	25:123	Thailand	UTD	6	WOMAC Pain; VAS Pain	WOMAC Function
Lequesne 2002 (76)	RCT	163 hips	102:61	France	63.2	24	VAS Pain	Lequesne Index
Lohmander 2014 (35)	RCT	170 knees	52:116	Bulgaria<br>Canada<br>Croatia<br>Finland<br>Germany<br>Poland<br>Serbia<br>Africa<br>Sweden	UTD	12	WOMAC Pain	WOMAC Function

For peer review only

				USA				
Maheu 2014 (8)	RCT	345 hips	159:186	France	62.2	36	WOMAC Pain; Global Hip Pain	Lequesne Index; WOMAC Function; Global handicap NRS
Marsh 2016 (34)	RCT	168 knees	57:112	Canada	UTD	24	WOMAC	WOMAC
McAlindion 2013 (33)	RCT	146 knees	57:89	USA	UTD	24	WOMAC Pain	WOMAC Function; Physical Test
Messier 2004 (32)	RCT	316 knees	89:227	USA	UTD	18	WOMAC Pain	WOMAC Function; Physical Test
Messier 2005 (77)	RCT	142 knees	37:105	USA	68.5	18	WOMAC Pain	WOMAC Function; Physical Test
Messier 2013 (78)	RCT	454 knees	128:325	USA	66	18	WOMAC Pain	WOMAC Function; Physical Test; SF-36 Physical
Michel 2005 (31)	RCT	300 knees	146:154	Switzerland	UTD	24	WOMAC Pain	WOMAC Function; Physical Test
Muraki 2014 (79)	Observational cohort	1558 knees	553:1005	Japan	67.0	40	WOMAC Pain	WOMAC Function;
Muraki 2015 (80)	Observational cohort	1525 knees	546:979	Japan	67.0	40	WOMAC Pain	WOMAC Function
Pavelka 2000 (30)	RCT	277 knees; 117 hips	109:285	Czech Republic	58	60	NA	Lequesne Index
Pavelka 2002 (81)	RCT	202 knees	45:157	Czech Republic	UTD	36	WOMAC Pain	WOMAC Function; Lequesne Index
Pham 2004 (29)	Observational cohort	301 knees	97:204	France	UTD	12	VAS Pain	Lequesne Index
Podsiadlo 2014 (28)	Observational cohort	114 knees	49:65	Australia	UTD	72	WOMAC Pain	WOMAC Function
Rat 2011 (82)	RCT	300 knees	118:182	France	67	6	SF-36 Body Pain; OAKHQOL Pain; VAS Pain	Lequense Index; SF-36 Physical; OAKHQOL Physical Activity

For peer review only - http://bmjopen.bmj.com/site/about/guidelines.xhtml

Raynauld 2011 (27)	RCT	123 knees	44:79	Canada	UTD	24	WOMAC Pain	WOMAC Function
Reginster 2001 (26)	RCT	212 knees	50:162	Belgium	UTD	36	WOMAC Pain	WOMAC Function
Reginster 2013 (83)	RCT	1371 knees	425:946	Australia<br>Austria<br>Belgium<br>Canada<br>Czech Republic<br>Denmark<br>Estonia<br>France<br>Germany<br>Italy<br>Lithuania<br>Netherlands<br>Poland<br>Portugal<br>Romania<br>Russian Federation<br>Spain<br>United Kingdom	62.9	36	WOMAC Pain; VAS Pain	WOMAC Function
Riddle 2015 (25)	Observational cohort	467 knees	209:258	USA	UTD	24	KOOS Pain	WOMAC Function
Romagnoli 2017 (84)	Observational cohort	105 knees	16:69	Italy	67.7	66	Knee Society Score Clinical; VAS Pain	Knee Society Score Function; ROM
Roman-Blas 2017 (24)	RCT	158 knees	26:132	Spain	UTD	6	WOMAC Pain; VAS Pain	WOMAC Function
Rozendaal 2008 (31)	RCT	222 hips	68:154	Netherlands	UTD	24	WOMAC Pain; VAS Pain	WOMAC Function
Sanchez-Ramirez 2015 (85)	Observational cohort	186 knees	59:127	Canada	61	24	WOAMC Pain	WOMAC Function; Physical Test
Sawitzke 2010 (86)	RCT	662 knees	215:447	USA	57	24	WOMAC Pain	WOMAC Function
Skou 2016 (87)	Observational cohort	1682 knees	434:818	Denmark	62.2	84	WOMAC Pain	PASE; Physical Test
Sowers 2011 (88)	Observational cohort	724 knees	0:363	USA	56	132	NA	WOMAC Function; Physical Test
Spector 2005 (89)	RCT	284 knees	115:169	UK	63.3	12	WOMAC Pain	WOMAC Function

For peer review only - http://bmjopen.bmj.com/site/about/guidelines.xhtml

Sun 2017 (90)	RCT	121 knees	31:90	Taiwan	63	6	WOMAC Pain; VAS Pain	WOMAC Function; Lequesne Index; Physical Test
Urish 2013 (22)	RCT	336 knees	96:67	USA	UTD	36	WOMAC	WOMAC
Valdes 2012 (17)	Observational cohort	860 knees; 928 hips	UTD	UK	UTD	38	WOMAC Pain	NA
Van der Esch 2016 (99)	Observational cohort	402 knees	64:137	Netherlands	61.2	24	NRS Pain	WOMAC Function; Physical Test
Weng 2009 (91)	RCT	264 knees	26:106	Taiwan	64	12	VAS Pain	Lequesne Index; ROM; Physical Test
White 2016 (92)	Observational cohort	2110 knees	992:118	USA	61.0	84	VAS Pain	WOMAC Function
Witt 2005 (93)	RCT	294 knees	70:154	Germany	64.0	12	WOMAC Pain; SF-36 Body Pain; VAS Pain	WOMAC Function; SF-36 Function
Yu 2016 (21)	Observational cohort	204 knees	74:130	Australia	UTD	12	KOOS Pain; VAS Pain	KOOS ADL; Physical Function
Yusuf 2011 (94)	Observational cohort	74 knees; 31 hips; 11 hip and knees	19:98	Netherlands	60	72	WOMAC Pain; SF-36 Body Pain; Pain on movement	WOMAC Function; SF-36 Function; Physical Test

ADLs – Activities of Daily Living; IKDC - International Knee Documentation Committee; KOOS - Knee Injury and Osteoarthritis Outcome Score; LEFS – Lower Extremity Functional Scale; NA – not applicable; NRS – numerical rating scale; PASE – Physical Activity Scale for the Elderly; RCT – randomised controlled trial; ROM – range of motion; OAKHQOL - osteoarthritis knee and hip quality of life questionnaire; SF-36 – Short Form-36; UCLA Activity - UK – United Kingdom; USA - United States of America; UTD – unable to determine; VAS – visual analogue scale; WOMAC - Western Ontario and McMaster Universities Osteoarthritis Index

For peer review only - http://bmjopen.bmj.com/site/about/guidelines.xhtml

Table 2. Meta-Analysis Results: Exhibit Knee OA

Variable	N	Effect Estimate	P-Value	Statistical Heterogeneity (I^2%)	GRADE Assessment
Gender	823	0.91 (0.48 to 1.72)*	0.78	87	Low quality evidence[1]
Age	823	1.46 (0.26 to 2.66)	0.02	0	Moderate quality evidence[2]
KL ≥2	823	2.04 (1.48 to 2.81)	<0.01	35	Moderate quality evidence[2]
BML Score	330	0.40 (-0.11 to 0.91)	0.13	NE	Low quality evidence[1]
Knee effusion score ≥1	823	1.35 (0.99 to 1.83)	0.05	0	Moderate quality evidence[2]
Baseline function score	330	-11.50 (-20.73 to -2.27)	0.01	NE	Low quality evidence[1]
Ethnicity (white)	493	1.03 (0.59 to 1.82)	0.91	NE	Low quality evidence[1]
BMI	823	-0.08 (-0.75 to 0.58)	0.81	0	Moderate quality evidence[2]
BML ≥1	493	1.44 (0.94 to 2.21)	0.09	NE	Low quality evidence[1]
Synovitis ≥1	493	1.24 (0.83 to 1.85)	0.30	NE	Low quality evidence[1]
Cartilage loss ≥2	493	2.11 (1.18 to 3.79)	0.01	NE	Low quality evidence[1]
Meniscal damage ≥1	493	1.83 (1.23 to 2.71)	0.003	NE	Low quality evidence[1]

BMI – body mass index; KL – Kellgren Lawrence scale; I2 – inconsistency-squared; N- number of participants in analysis; NE – not estimable

* - random effects model analysis

[1]GRADE – Outcomes downgraded one level due to risk of bias, two level due to imprecision and inconsistency; [2]GRADE – Outcomes downgraded one level due to risk of bias

For peer review only

For peer review only - http://bmjopen.bmj.com/site/about/guidelines.xhtml

Figure 1

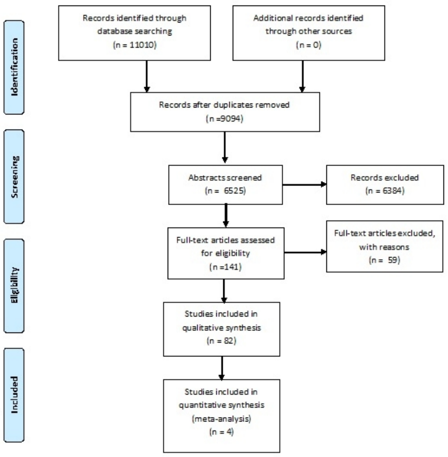

304x190mm (96 x 96 DPI)

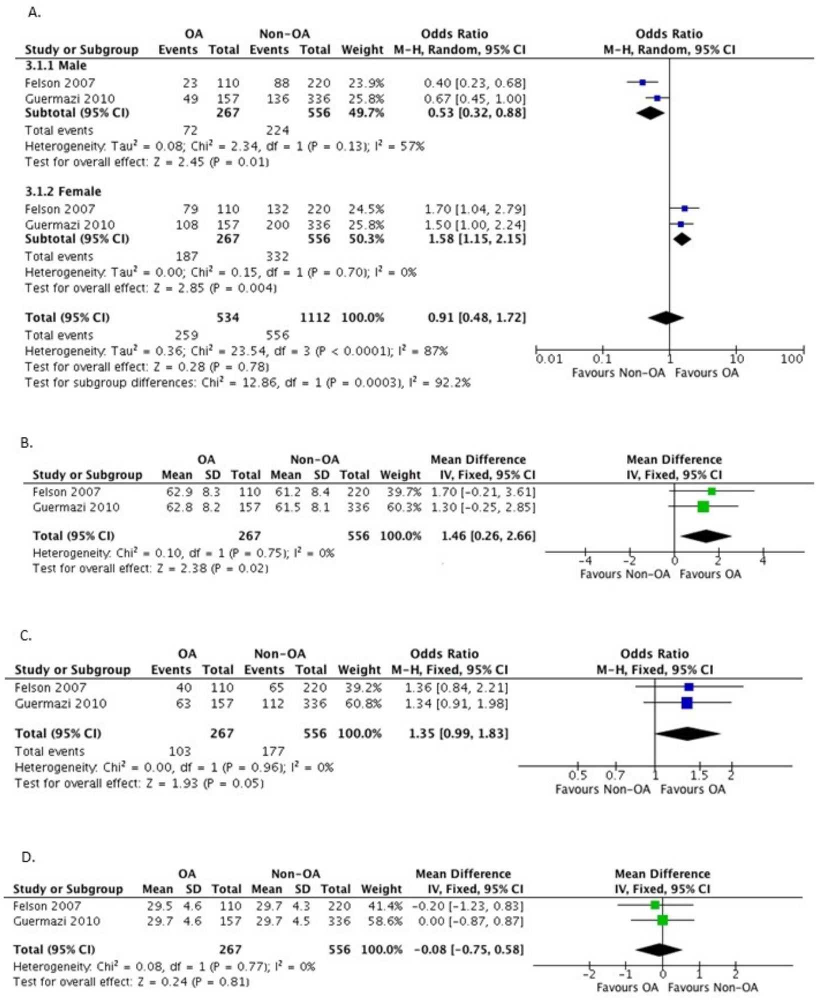

Figure 2a-2d

190x275mm (96 x 96 DPI)

Supplementary File 1: Methodological appraisal results based on the Downs and Black Observational Studies Checklist

	Downs and Black Observational Studies Checklist Items																		
	**1**	**2**	**3**	**4**	**5**	**6**	**7**	**8**	**9**	**10**	**11**	**12**	**13**	**14**	**15**	**16**	**17**	**18**	**Total**
Ahedi 2014	1	1	1	0	1	1	0	1	1	1	1	UTD	1	1	1	UTD	1	0	13
Amin 2008	1	1	0	1	1	1	1	0	0	UTD	1	1	1	1	UTD	1	1	0	12
Antony 2017	1	1	1	2	1	1	1	0	1	0	UTD	UTD	1	1	1	UTD	1	0	13
Baselga Garcia-Escudero 2015	1	1	1	0	1	1	1	1	UTD	UTD	1	1	1	1	UTD	0	1	1	13
Bevers 2015	1	1	1	2	0	1	1	1	UTD	0	1	1	1	1	1	1	1	0	15
Birmingham 2009	1	1	1	1	1	1	1	1	1	1	UTD	1	1	UTD	1	1	1	0	15
Chandrasekaran 2016A	1	1	1	1	1	1	1	1	0	UTD	1	1	1	1	UTD	1	1	1	15
Chandrasekaran 2016B	1	1	1	1	1	1	0	1	0	UTD	1	1	1	1	UTD	1	UTD	1	13
Davis 2017	1	1	1	0	0	1	1	0	1	1	1	UTD	1	1	1	UTD	1	0	12
Dowsey 2012	1	1	1	1	1	1	1	1	1	1	1	UTD	1	1	1	1	1	0	16
Eckstein 2013	1	1	1	2	1	1	1	1	1	1	1	1	1	UTD	1	1	1	0	17
Felson 2013	1	1	1	1	0	1	1	1	0	UTD	1	1	1	UTD	1	1	1	0	13
Filardo 2015	1	1	1	2	0	1	1	1	1	1	1	1	1	UTD	1	1	1	0	16
Glass 2013	1	1	1	2	0	1	1	1	1	1	1	1	1	1	1	1	1	0	17
Guermazin 2010	1	1	1	2	0	1	1	1	1	1	1	1	1	UTD	1	1	1	0	16
Hamilton 2017	1	1	0	0	1	1	1	1	UTD	UTD	1	1	1	1	UTD	UTD	1	1	12
Henriksen 2013	1	1	1	2	1	1	1	1	UTD	UTD	1	1	1	UTD	UTD	1	1	1	15
Huizinga 2017	1	1	1	0	1	1	1	0	UTD	UTD	1	1	1	1	UTD	0	1	0	11
Khan 2013	1	1	1	1	0	1	1	1	1	1	0	1	1	UTD	1	1	1	0	14
Kinds 2012	1	1	1	1	0	1	1	1	1	1	1	1	1	UTD	1	1	1	0	15
Messier 2005	1	1	0	2	1	1	0	1	1	UTD	1	1	1	UTD	1	1	1	0	14
Muraki 2014	1	1	1	1	1	1	1	1	1	UTD	1	1	1	1	0	1	1	0	15
Muraki 2015	1	1	1	2	1	1	1	1	1	UTD	1	1	1	1	0	1	1	0	16
Podsiadlo 2014	1	1	1	1	0	1	1	1	UTD	UTD	1	1	1	UTD	UTD	1	1	0	12
Rat 2011	1	1	1	1	1	1	1	1	UTD	UTD	1	1	1	1	0	UTD	1	1	14
Raynauld 2011	1	0	1	2	1	1	1	1	1	UTD	1	1	1	1	1	1	1	0	16
Riddle 2015	1	1	1	2	1	1	0	0	1	1	0	1	1	1	1	1	1	1	16
Romagnoli 2017	1	0	1	0	0	1	1	1	1	1	1	1	1	UTD	1	UTD	1	1	13

For peer review only

Sanchez-Ramirez 2015	1	1	1	2	1	1	1	1	1	1	1	1	1	UTD	1	1	1	0	17
Skou 2016	1	1	1	2	0	1	1	1	1	1	1	1	1	UTD	1	1	1	0	16
Sowers 2011	1	1	1	0	1	1	1	1	1	UTD	1	1	1	UTD	1	0	0	0	12
Urish 2013	1	1	1	1	1	1	1	1	1	1	1	1	1	1	1	0	1	0	16
Valder 2012	1	1	1	1	1	1	0	1	UTD	UTD	1	1	1	0	1	1	0	0	12
Van der Esch 2016	1	1	1	1	1	1	1	1	1	1	1	1	1	UTD	1	0	1	0	15
White 2016	1	1	1	2	0	1	1	0	1	1	1	1	1	1	0	1	1	0	15
Yu 2016	1	1	1	1	1	1	1	1	1	1	1	1	1	1	1	1	1	0	17
Yusuf 2011	1	1	1	1	1	1	1	0	UTD	UTD	1	1	1	UTD	1	1	1	0	13
Total with score >0	37	35	34	30	15	37	32	30	24	18	33	33	37	20	25	26	34	8	-

Checklist items

1. Is the hypothesis/aim/objective of the study clearly described?
2. Are the main outcomes to be measured clearly described in the Introduction or Methods section?
3. Are the characteristics of the patients included in the study clearly described?
4. Are the distributions of principal confounders in each group of subjects to be compared clearly described?
5. Are the main findings of the study clearly described?
6. Does the study provide estimates of the random variability in the data for the main outcomes?
7. Have the characteristics of patients lost to follow-up been described?
8. Have actual probability values been reported (e.g. 0.035 rather than <0.05) for the main outcomes except where the probability value is less than 0.001?
9. Were the subjects asked to participate in the study representative of the entire population from which they were recruited?
10. Were those subjects who were prepared to participate representative of the entire population from which they were recruited?
11. If any of the results of the study were based on "data dredging", was this made clear?
12. Were the statistical tests used to assess the main outcomes appropriate?
13. Were the main outcome measures used accurate (valid and reliable)?
14. Were study participants in different groups (trials and cohort studies) or were the cases and controls (case-control studies) recruited over the same period of time?
15. Were the participants in different groups (trials and cohort studies) or were the cases and controls (case-control studies) recruited from the same population?
16. Was there adequate adjustment for confounding in the analyses from which the main findings were drawn?

For peer review only - http://bmjopen.bmj.com/site/about/guidelines.xhtml

17. Were losses of patients to follow-up taken into account?
18. Did the study mention having conducted a power analysis to determine the sample size needed to detect a significant difference in effect size for one or more outcome measures?

Footnote

UTD: Unable To Determine
2: Yes
1: Yes/partially
0: No

For peer review only

For peer review only - http://bmjopen.bmj.com/site/about/guidelines.xhtml

Supplementary File 2: Methodological appraisal results based on the Downs and Black non-RCT Checklist

	Downs and Black Non-Randomised Controlled Trial Checklist Items																		
	**1**	**2**	**3**	**4**	**5**	**6**	**7**	**8**	**9**	**10**	**11**	**12**	**13**	**14**	**15**	**16**	**17**	**18**	**Total**
Ahedi (54)	1	1	1	0	1	1	0	1	1	1	1	UC	1	1	1	UC	1	0	13
Amin (55)	1	1	0	1	1	1	1	0	0	UC	1	1	1	1	UC	1	1	0	12
Antony (560	1	1	1	2	1	1	1	0	1	0	UC	UC	1	1	1	UC	1	0	13
Baselga Garcia-Escudero (59)	1	1	1	0	1	1	1	1	UC	UC	1	1	1	1	UC	0	1	1	13
Bevers (60)	1	1	1	2	0	1	1	1	UC	0	1	1	1	1	1	1	1	0	15
Birmingham (61)	1	1	1	1	1	1	1	1	1	1	UC	1	1	UC	1	1	1	0	15
Chandrasekaran (48)	1	1	1	1	1	1	1	1	0	UC	1	1	1	1	UC	1	1	1	15
Chandrasekaran (47)	1	1	1	1	1	1	0	1	0	UC	1	1	1	1	UC	1	UC	1	13
Davis (19)	1	1	1	0	0	1	1	0	1	1	1	UC	1	1	1	UC	1	0	12
Dowsey (65)	1	1	1	1	1	1	1	1	1	1	1	UC	1	1	1	1	1	0	16
Felson (66)	1	1	1	1	0	1	1	1	0	UC	1	1	1	UC	1	1	1	0	13
Felson (67)	1	1	1	1	0	1	1	1	0	UC	1	1	1	UC	1	1	1	0	13
Glass (42)	1	1	1	2	0	1	1	1	1	1	1	1	1	1	1	1	1	0	17
Guermazin (41)	1	1	1	2	0	1	1	1	1	1	1	1	1	UC	1	1	1	0	16
Hamilton (68)	1	1	0	0	1	1	1	1	UC	UC	1	1	1	1	UC	UC	1	1	12
Huizinga (73)	1	1	1	0	1	1	1	0	UC	UC	1	1	1	1	UC	0	1	0	11
Khan (74)	1	1	1	1	0	1	1	1	1	1	0	1	1	UC	1	1	1	0	14
Muraki (79)	1	1	1	1	1	1	1	1	1	UC	1	1	1	1	0	1	1	0	15
Muraki (80)	1	1	1	2	1	1	1	1	1	UC	1	1	1	1	0	1	1	0	16
Pham (29)	1	1	1	1	1	1	1	1	UC	UC	1	1	1	0	1	1	1	0	14
Podsiadlo (28)	1	1	1	1	0	1	1	1	UC	UC	1	1	1	UC	UC	1	1	0	12
Riddle (25)	1	1	1	2	1	1	0	0	1	1	0	1	1	1	1	1	1	1	16
Romagnoli (84)	1	0	1	0	0	1	1	1	1	1	1	1	1	UC	1	UC	1	1	13
Sanchez-Ramirez (85)	1	1	1	2	1	1	1	1	1	1	1	1	1	UC	1	1	1	0	17
Skou (87)	1	1	1	2	0	1	1	1	1	1	1	1	1	UC	1	1	1	0	16
Sowers (88)	1	1	1	0	1	1	1	1	1	UC	1	1	1	UC	1	0	0	0	12
Valder (17)	1	1	1	1	1	1	0	1	UC	UC	1	1	1	0	1	1	0	0	12
Van der Esch (99)	1	1	1	1	1	1	1	1	1	1	1	1	1	UC	1	0	1	0	15
White (92)	1	1	1	2	0	1	1	0	1	1	1	1	1	1	0	1	1	0	15
Yu (21)	1	1	1	1	1	1	1	1	1	1	1	1	1	1	1	1	1	0	17
Yusuf (94)	1	1	1	1	1	1	1	0	UC	UC	1	1	1	UC	1	1	1	0	13

For peer review only

For peer review only - http://bmjopen.bmj.com/site/about/guidelines.xhtml

UC: Unclear; 2: Yes; 1: Yes/partially; 0: No

Checklist items

19. Is the hypothesis/aim/objective of the study clearly described?
20. Are the main outcomes to be measured clearly described in the Introduction or Methods section?
21. Are the characteristics of the patients included in the study clearly described?
22. Are the distributions of principal confounders in each group of subjects to be compared clearly described?
23. Are the main findings of the study clearly described?
24. Does the study provide estimates of the random variability in the data for the main outcomes?
25. Have the characteristics of patients lost to follow-up been described?
26. Have actual probability values been reported (e.g. 0.035 rather than <0.05) for the main outcomes except where the probability value is less than 0.001?
27. Were the subjects asked to participate in the study representative of the entire population from which they were recruited?
28. Were those subjects who were prepared to participate representative of the entire population from which they were recruited?
29. If any of the results of the study were based on "data dredging", was this made clear?
30. Were the statistical tests used to assess the main outcomes appropriate?
31. Were the main outcome measures used accurate (valid and reliable)?
32. Were study participants in different groups (trials and cohort studies) or were the cases and controls (case-control studies) recruited over the same period of time?
33. Were the participants in different groups (trials and cohort studies) or were the cases and controls (case-control studies) recruited from the same population?
34. Was there adequate adjustment for confounding in the analyses from which the main findings were drawn?
35. Were losses of patients to follow-up taken into account?
36. Did the study mention having conducted a power analysis to determine the sample size needed to detect a significant difference in effect size for one or more outcome measures?

For peer review only - http://bmjopen.bmj.com/site/about/guidelines.xhtml

Supplementary File 3: Methodological appraisal results based on the Downs and Black RCT Studies Checklist

	Downs and Black Randomised Controlled Trial Checklist Items																											
	**1**	**2**	**3**	**4**	**5**	**6**	**7**	**8**	**9**	**10**	**11**	**12**	**13**	**14**	**15**	**16**	**17**	**18**	**19**	**20**	**21**	**22**	**23**	**24**	**25**	**26**	**27**	**Total**
Akelman (20)	1	1	1	1	2	1	1	1	1	1	1	1	1	1	1	1	1	1	1	1	0	1	1	1	UC	1	1	**26**
Arden (57)	1	1	1	1	1	1	1	1	1	0	1	1	1	1	1	1	UC	1	1	1	1	1	1	1	0	1	UC	**23**
Ayral (58)	1	1	1	1	1	1	1	1	1	1	0	UC	UC	1	1	1	1	1	1	1	0	1	1	UC	UC	1	0	**20**
Bingham (53)	1	1	1	1	1	1	1	1	1	1	1	1	1	1	UC	1	1	1	1	1	0	1	0	UC	1	1	0	**22**
Bisicchia (52)	1	0	1	1	0	1	1	1	1	1	1	1	1	0	1	UC	1	1	1	1	1	1	1	0	0	1	0	**20**
Brandt (62)	1	1	1	1	1	0	1	1	1	1	UC	UC	UC	UC	1	1	1	1	1	1	UC	1	1	UC	0	1	0	**18**
Brown (50)	1	1	1	1	1	1	1	1	1	0	UC	UC	UC	1	1	1	1	1	1	1	UC	UC	1	UC	UC	UC	1	**18**
Brown (51)	1	1	1	1	1	1	1	1	1	0	UC	UC	UC	1	1	1	1	1	1	1	UC	UC	1	1	UC	1	1	**19**
Bruyere (63)	1	1	1	1	1	0	1	0	1	1	UC	UC	1	1	1	1	1	1	UC	1	UC	UC	1	UC	UC	1	1	**18**
Campbell (49)	1	1	1	1	0	0	0	1	1	0	1	UC	1	1	1	1	1	1	1	1	1	1	1	1	UC	1	0	**20**
Conrozier (64)	1	1	1	1	0	0	1	1	1	1	UC	UC	UC	1	1	1	1	0	1	1	0	1	1	1	0	1	UC	**18**
Dougados (46)	1	1	1	1	1	1	1	1	1	0	1	0	0	UC	UC	1	1	1	1	1	0	UC	1	UC	1	1	UC	**18**
Eckstein (45)	1	1	1	1	2	1	1	1	1	1	1	1	1	1	1	1	1	1	1	1	UC	1	1	1	1	1	0	**26**
Ettinger (44)	1	1	1	1	1	0	1	1	1	1	UC	UC	0	0	UC	1	1	1	UC	1	0	1	1	1	1	1	1	**19**
Filardo (43)	1	1	1	1	2	1	1	1	1	1	1	1	1	1	1	1	1	1	1	1	1	1	1	1	1	1	0	**25**
Hellio le Graverand (69)	1	1	1	1	1	0	1	1	1	1	UC	UC	UC	UC	1	1	UC	1	1	1	0	UC	1	1	UC	1	0	**17**
Henriksen (40)	1	1	1	1	2	1	1	1	0	1	UC	UC	1	0	1	1	1	1	1	1	1	1	1	1	1	1	1	**24**
Hill (5)	1	1	1	1	0	0	1	1	1	1	1	0	UC	1	1	1	1	1	1	1	0	1	1	1	0	1	1	**21**
Hochberg (70)	1	1	1	1	1	1	1	1	1	1	UC	UC	UC	1	UC	1	1	1	UC	1	0	UC	1	1	UC	1	0	**18**
Hoeksma (71)	1	1	1	1	0	1	1	1	1	0	1	1	0	0	1	1	1	1	0	1	1	1	1	1	UC	1	1	**21**
Housman (39)	1	1	1	1	0	0	1	1	1	0	0	UC	0	1	0	1	1	1	UC	1	0	1	1	UC	0	1	1	**16**
Huang (72)	1	1	1	1	0	1	1	0	1	0	UC	UC	UC	UC	1	1	UC	1	1	1	1	UC	1	UC	0	1	1	**16**
Jin (6)	1	1	1	1	0	1	1	1	0	1	UC	UC	0	1	1	1	1	1	UC	1	0	1	1	1	0	1	0	**18**
Karsdal (38)	1	1	1	1	1	0	1	1	1	0	UC	UC	UC	1	1	1	1	1	0	1	0	UC	1	1	1	1	UC	UC
Katz (37)	1	1	1	1	2	1	1	1	1	0	0	0	0	0	0	1	0	1	0	1	0	1	1	0	1	1	UC	**17**
Kim (75)	1	0	1	1	0	1	1	0	1	1	UC	UC	UC	1	1	1	0	1	1	1	UC	1	1	1	0	1	0	**17**

For peer review only - http://bmjopen.bmj.com/site/about/guidelines.xhtml

Kinds (18)	1	1	1	1	1	0	1	1	1	1	1	1	1	1	1	1	1	1	1	1	1	1	1	1	1	1	0	**25**
Kongtharvonskul (36)	1	1	1	1	2	1	1	1	0	1	1	UC	0	1	1	1	1	1	0	1	1	1	1	1	1	1	0	**23**
Lequesne (76)	1	1	1	1	1	1	1	1	1	1	UC	UC	0	1	1	1	1	1	1	1	0	UC	1	1	1	1	0	**21**
Lohmander (35)	1	1	1	1	0	1	1	1	1	1	UC	UC	0	1	1	0	1	1	1	1	0	1	1	1	0	1	1	**20**
Maheu (8)	1	1	1	1	0	1	0	1	0	1	UC	UC	0	1	1	1	1	UC	1	1	0	1	1	1	1	0	0	**17**
Marsh (34)	1	1	0	1	2	1	1	0	1	1	UC	UC	1	0	0	1	1	1	UC	1	UC	UC	UC	UC	1	1	0	**16**
McAlindion (33)	1	1	1	1	1	1	1	1	0	1	0	UC	0	1	1	1	1	1	1	1	0	1	1	1	UC	1	0	**20**
Messier (32)	1	1	1	1	1	0	1	1	0	0	0	UC	0	0	1	1	1	1	0	1	UC	1	1	1	1	0	1	**17**
Meissier (77)	1	1	0	1	2	1	1	1	0	1	1	1	1	1	1	1	1	1	1	1	1	1	1	1	1	1	0	**25**
Messier (78)	1	1	1	1	2	1	1	1	0	1	1	1	0	0	1	1	1	1	0	1	1	1	1	UC	1	1	1	**23**
Michel (31)	1	1	1	1	0	0	1	1	0	1	UC	UC	1	1	1	1	1	1	1	1	UC	1	1	1	0	0	1	**19**
Pavelka (30)	1	1	1	0	1	0	1	1	0	1	UC	UC	0	1	1	1	1	1	1	1	0	1	1	UC	1	1	0	**18**
Pavelka (81)	1	1	1	1	1	0	1	1	1	1	1	UC	1	1	1	1	1	1	1	1	1	1	1	1	1	1	1	**25**
Rat (82)	1	1	1	1	1	1	1	1	1	1	1	1	1	1	1	1	1	1	1	1	1	0	1	1	UC	1	1	**25**
Raynauld (27)	1	1	1	1	2	1	1	1	1	1	1	1	1	0	1	1	1	1	1	1	1	1	1	1	1	1	0	**26**
Reginster (83)	1	1	1	1	1	1	1	1	0	1	UC	UC	0	1	1	1	1	1	1	1	0	1	1	1	1	1	1	**22**
Reginster (26)	1	1	1	1	1	0	1	1	0	1	0	UC	1	1	1	1	1	1	1	1	1	1	1	1	UC	1	1	**22**
Roman-Blas (24)	1	1	1	1	1	0	1	1	1	1	UC	UC	0	1	1	1	1	1	0	1	0	UC	1	1	1	1	0	**19**
Rozendaal (31)	1	1	1	1	2	1	1	1	1	0	UC	UC	0	1	1	0	1	1	1	1	UC	1	1	1	1	1	UC	**21**
Sawitzke (86)	1	0	1	1	2	0	1	1	0	1	1	UC	0	1	1	1	1	1	1	1	UC	UC	1	UC	1	UC	UC	UC
Spector (89)	1	1	1	1	2	0	1	1	1	1	UC	UC	0	UC	UC	1	1	1	0	1	0	UC	1	UC	1	1	0	**17**
Sun (90)	1	1	1	1	1	1	1	1	1	1	UC	UC	1	1	1	1	1	1	1	1	UC	1	1	1	1	1	1	**24**
Urish (22)	1	1	1	1	1	1	1	1	1	1	1	1	1	0	1	1	1	1	1	1	1	1	1	1	0	1	0	**24**
Weng (91)	1	1	1	1	0	1	1	0	1	0	UC	UC	1	0	UC	1	1	1	1	1	UC	UC	1	1	0	1	1	**17**
Witt (93)	1	1	1	1	1	1	1	1	1	1	UC	UC	1	0	0	1	1	1	1	1	UC	1	1	1	1	1	1	**22**

UC: Unclear; 2: Yes; 1: Yes/partially; 0: No

Checklist items

1. Is the hypothesis/aim/objective of the study clearly described?
2. Are the main outcomes to be measured clearly described in the Introduction or Methods section?

For peer review only - http://bmjopen.bmj.com/site/about/guidelines.xhtml

3. Are the characteristics of the patients included in the study clearly described?
4. Are the interventions of interest clearly described?
5. Are the distributions of principal confounders in each group of subjects to be compared clearly described?
6. Are the main findings of the study clearly described?
7. Does the study provide estimates of the random variability in the data for the main outcomes?
8. Have all important adverse events that may be a consequence of the intervention been reported?
9. Have the characteristics of patients lost to follow-up been described?
10. Have actual probability values been reported (e.g. 0.035 rather than <0.05) for the main outcomes except where the probability value is less than 0.001?
11. Were the subjects asked to participate in the study representative of the entire population from which they were recruited?
12. Were those subjects who were prepared to participate representative of the entire population from which they were recruited?
13. Were the staff, places, and facilities where the patients were treated, representative of the treatment the majority of patients receive?
14. Was an attempt made to blind study subjects to the intervention they have received?
15. Was an attempt made to blind those measuring the main outcomes of the Intervention?
16. If any of the results of the study were based on "data dredging", was this made clear?
17. In trials and cohort studies, do the analyses adjust for different lengths of follow-up of patients, or in case-control studies, is the time period between the intervention and outcome the same for cases and controls?
18. Were the statistical tests used to assess the main outcomes appropriate?
19. Was compliance with the intervention/s reliable?
20. Were the main outcome measures used accurate (valid and reliable)?
21. Were the patients in different intervention groups (trials and cohort studies) or were the cases and controls (case-control studies) recruited from the same population?
22. Were study subjects in different intervention groups (trials and cohort studies) or were the cases and controls (case-control studies) recruited over the same period of time?
23. Were study subjects randomized to intervention groups?
24. Was the randomized intervention assignment concealed from both patients and health care staff until recruitment was complete and irrevocable?
25. Was there adequate adjustment for confounding in the analyses from which the main findings were drawn?
26. Were losses of patients to follow-up taken into account?
27. Was there sufficient power to detect treatment effect at significance level of 0.05?

For peer review only - http://bmjopen.bmj.com/site/about/guidelines.xhtml

PRISMA 2009 Checklist

Section/topic	#	Checklist item	Reported on page #
**TITLE**			
Title	1	Identify the report as a systematic review, meta-analysis, or both.	Title
**ABSTRACT**			
Structured summary	2	Provide a structured summary including, as applicable: background; objectives; data sources; study eligibility criteria, participants, and interventions; study appraisal and synthesis methods; results; limitations; conclusions and implications of key findings; systematic review registration number.	Abstract including registration
**INTRODUCTION**			
Rationale	3	Describe the rationale for the review in the context of what is already known.	Introduction, Paragraphs 1-3
Objectives	4	Provide an explicit statement of questions being addressed with reference to participants, interventions, comparisons, outcomes, and study design (PICOS).	Introduction, Paragraph 3
**METHODS**			
Protocol and registration	5	Indicate if a review protocol exists, if and where it can be accessed (e.g., Web address), and, if available, provide registration information including registration number.	Abstract, Registration & Methods Paragraph 1
Eligibility criteria	6	Specify study characteristics (e.g., PICOS, length of follow-up) and report characteristics (e.g., years considered, language, publication status) used as criteria for eligibility, giving rationale.	Methods, Study Identification
Information sources	7	Describe all information sources (e.g., databases with dates of coverage, contact with study authors to identify additional studies) in the search and date last searched.	Methods, Search Strategy
Search	8	Present full electronic search strategy for at least one database, including any limits used, such that it could be repeated.	Supplementary File 1
Study selection	9	State the process for selecting studies (i.e., screening, eligibility, included in systematic review, and, if applicable, included in the meta-analysis).	Methods, Study Identification

For peer review only

PRISMA 2009 Checklist

Data collection process	10	Describe method of data extraction from reports (e.g., piloted forms, independently, in duplicate) and any processes for obtaining and confirming data from investigators.	Methods, Data Extraction
Data items	11	List and define all variables for which data were sought (e.g., PICOS, funding sources) and any assumptions and simplifications made.	Methods, Data Extraction & Methods Outcomes
Risk of bias in individual studies	12	Describe methods used for assessing risk of bias of individual studies (including specification of whether this was done at the study or outcome level), and how this information is to be used in any data synthesis.	Methods, Quality Assessment
Summary measures	13	State the principal summary measures (e.g., risk ratio, difference in means).	Methods, Data Analysis, Paragraph 1
Synthesis of results	14	Describe the methods of handling data and combining results of studies, if done, including measures of consistency (e.g., I^2) for each meta-analysis.	Methods, Data Analysis, Paragraph 1

Section/topic	#	Checklist item	Reported on page #
Risk of bias across studies	15	Specify any assessment of risk of bias that may affect the cumulative evidence (e.g., publication bias, selective reporting within studies).	Methods, Quality Assessment
Additional analyses	16	Describe methods of additional analyses (e.g., sensitivity or subgroup analyses, meta-regression), if done, indicating which were pre-specified.	Methods, Data Analysis, Paragraph 2
**RESULTS**			
Study selection	17	Give numbers of studies screened, assessed for eligibility, and included in the review, with reasons for exclusions at each stage, ideally with a flow diagram.	Results, Search Strategy
Study characteristics	18	For each study, present characteristics for which data were extracted (e.g., study size, PICOS, follow-up period) and provide the citations.	Results, Characteristics of Included Studies

For peer review only

For peer review only - http://bmjopen.bmj.com/site/about/guidelines.xhtml

PRISMA 2009 Checklist

Risk of bias within studies	19	Present data on risk of bias of each study and, if available, any outcome level assessment (see item 12).	Results, Methodological Quality Assessment
Results of individual studies	20	For all outcomes considered (benefits or harms), present, for each study: (a) simple summary data for each intervention group (b) effect estimates and confidence intervals, ideally with a forest plot.	Figures 2a-d
Synthesis of results	21	Present results of each meta-analysis done, including confidence intervals and measures of consistency.	Results Table 2
Risk of bias across studies	22	Present results of any assessment of risk of bias across studies (see Item 15).	Supplementary File 2 and 3; Results, Methodological Quality
Additional analysis	23	Give results of additional analyses, if done (e.g., sensitivity or subgroup analyses, meta-regression [see Item 16]).	Results, Meta-Analysis, Knee
**DISCUSSION**			
Summary of evidence	24	Summarize the main findings including the strength of evidence for each main outcome; consider their relevance to key groups (e.g., healthcare providers, users, and policy makers).	Discussion, Paragraph 1
Limitations	25	Discuss limitations at study and outcome level (e.g., risk of bias), and at review-level (e.g., incomplete retrieval of identified research, reporting bias).	Discussion, Paragraph 5
Conclusions	26	Provide a general interpretation of the results in the context of other evidence, and implications for future research.	Discussion, Paragraph 2-4
**FUNDING**			
Funding	27	Describe sources of funding for the systematic review and other support (e.g., supply of data); role of funders for the systematic review.	Declarations, Funding sources

From: Moher D, Liberati A, Tetzlaff J, Altman DG, The PRISMA Group (2009). Preferred Reporting Items for Systematic Reviews and Meta-Analyses: The PRISMA Statement. PLoS Med 6(7): e1000097. doi:10.1371/journal.pmed1000097

For more information, visit: **www.prisma-statement.org**.

For peer review only - http://bmjopen.bmj.com/site/about/guidelines.xhtml

BMJ Open

Risk factors for pain and functional impairment in people with knee and hip osteoarthritis: a systematic review and meta-analysis

Journal:	*BMJ Open*
Manuscript ID	bmjopen-2020-038720.R2
Article Type:	Original research
Date Submitted by the Author:	03-Jun-2020
Complete List of Authors:	Sandhar, Sandeep; University of London St George's, Institute for Infection and Immunity<br>Smith, Toby O.; University of Oxford, Nuffield Department of Orthopaedics and Musculoskeletal Sciences<br>Toor, Kavanbir; University of London St George's, Institute for Infection and Immunity<br>Howe, Franklyn ; University of London St George's, Molecular and Clinical Sciences Research Institute<br>Sofat, Nidhi; University of London St George's, Institute for Infection and Immunity
**Primary Subject Heading**:	Rheumatology
Secondary Subject Heading:	Diagnostics
Keywords:	Knee < ORTHOPAEDIC & TRAUMA SURGERY, Hip < ORTHOPAEDIC & TRAUMA SURGERY, PAIN MANAGEMENT, RHEUMATOLOGY

SCHOLARONE™
Manuscripts

I, the Submitting Author has the right to grant and does grant on behalf of all authors of the Work (as defined in the below author licence), an exclusive licence and/or a non-exclusive licence for contributions from authors who are: i) UK Crown employees; ii) where BMJ has agreed a CC-BY licence shall apply, and/or iii) in accordance with the terms applicable for US Federal Government officers or employees acting as part of their official duties; on a worldwide, perpetual, irrevocable, royalty-free basis to BMJ Publishing Group Ltd ("BMJ") its licensees and where the relevant Journal is co-owned by BMJ to the co-owners of the Journal, to publish the Work in this journal and any other BMJ products and to exploit all rights, as set out in our licence.

The Submitting Author accepts and understands that any supply made under these terms is made by BMJ to the Submitting Author unless you are acting as an employee on behalf of your employer or a postgraduate student of an affiliated institution which is paying any applicable article publishing charge ("APC") for Open Access articles. Where the Submitting Author wishes to make the Work available on an Open Access basis (and intends to pay the relevant APC), the terms of reuse of such Open Access shall be governed by a Creative Commons licence – details of these licences and which Creative Commons licence will apply to this Work are set out in our licence referred to above.

Other than as permitted in any relevant BMJ Author's Self Archiving Policies, I confirm this Work has not been accepted for publication elsewhere, is not being considered for publication elsewhere and does not duplicate material already published. I confirm all authors consent to publication of this Work and authorise the granting of this licence.

For peer review only

Title: Risk factors for pain and functional impairment in people with knee and hip osteoarthritis: a systematic review and meta-analysis

Concise Title: Factors associated with pain and impaired function in OA

Authors: Sandeep Sandhar,[1] Toby Smith,[2,3] Kavanbir Toor,[1] Franklyn Howe,[4] Nidhi Sofat[1]

Affiliations

1. Institute for Infection and Immunity Research, St Georges, University of London, London, UK

2. Nuffield Department of Orthopaedics, Rheumatology and Musculoskeletal Sciences, University of Oxford, Oxford, UK

3. Faculty of Medicine and Health Sciences, University of East Anglia, Norwich, UK

4. Neurosciences Research Centre, St Georges, University of London, London, UK

Corresponding author: Professor Nidhi Sofat (nsofat@sgul.ac.uk), Institute for Infection and Immunity, St Georges, University of London, London, SW17 0RE

Work done on behalf of the OA Tech working research group.

Email addresses: ssandhar@sgul.ac.uk (Sandeep Sandhar), toby.smith@ndorms.ox.ac.uk (Toby Smith), howefa@sgul.ac.uk (Franklyn Howe), m1604502@sgul.ac.uk (Kavanbir Toor), nsofat@sgul.ac.uk (Nidhi Sofat)

ABSTRACT

Objective: To identify risk factors for pain and functional deterioration in people with knee and hip osteoarthritis (OA) to form the basis of a future 'stratification tool' for OA development or progression.

Design: Systematic review and meta-analysis

Methods: An electronic search of the literature databases: MEDLINE, EMBASE, CINAHL, MEDLINE and Web of Science (1990-February 2020) was conducted. Studies which identified risk factors for pain and functional deterioration to knee and hip OA were included. Where data and study heterogeneity permitted, meta-analyses presenting mean difference (MD) and odd ratios (OR) with corresponding 95% confidence intervals (CI) were undertaken. Where this was not possible, a narrative analysis was undertaken. The Downs & Black tool assessed methodological quality of selected studies before data extraction. Pooled analysis outcomes were assessed and reported using the GRADE approach.

Results: 82 studies (41,810 participants) were included. On meta-analysis: there was moderate quality evidence that knee OA pain was associated with factors including: Kellgren and Lawrence≥ 2 (MD: 2.04, 95% CI:1.48,2.81; p<0.01), increasing age (MD: 1.46, 95% CI:0.26,2.66; p=0.02) and whole-organ MRI scoring method Knee effusion score ≥1 (OR: 1.35, 95% CI: 0.99,1.83; p=0.05). On narrative analysis: knee OA pain was associated with factors including WORMS meniscal damage≥1 (OR: 1.83). Predictors of joint pain in hip OA were large acetabular bone marrow lesions (OR: 5.23), chronic widespread pain (OR: 5.02) and large hip BMLs (OR: 4.43).

For peer review only - http://bmjopen.bmj.com/site/about/guidelines.xhtml

Conclusions: Our study identified risk factors for clinical pain in OA by imaging measures that can assist in predicting and stratifying people with knee/hip OA. A 'stratification tool' combining verified risk factors that we have identified, would allow selective stratification based on pain and structural outcomes in OA.

PROSPERO Registration: CRD42018117643

ARTICLE SUMMARY: strengths and limitations of this study

- This study has been reported in accordance with the Preferred Reporting Items for Systematic Reviews and Meta-Analyses reporting checklist.
- Analyses have been undertaken respecting potential sources of known statistical heterogeneity.
- Searches included both published and unpublished sources of literature to reduce the risk of omitting potentially eligible data.
- There was a paucity of available data to permit meta-analyses of risk factors for pain and functional impairment.
- The variability in methods of assessing risk and reporting of frequency of risk characteristics limited analyses

For peer review only

For peer review only - http://bmjopen.bmj.com/site/about/guidelines.xhtml

INTRODUCTION

It has been reported that over 30.8 million US adults suffer from osteoarthritis (OA)(1). Between 1990-2010, the years lived with disability worldwide caused by OA increased from 10.5 million to 17.1 million, an increase of 62.9%(2). Current OA treatment lacks any disease-modifying treatments with a predominance to manage symptoms rather than modify underlying disease(3). The clinical symptoms of OA can be assessed using several questionnaires, the most common of which is the Western Ontario McMaster Arthritic Index (WOMAC)(4, 5, 6). Although pain is recognised as an important outcome measure in OA, it is not clear what the optimal assessment tools are in OA and how they relate to other risk factors.

OA has various subtypes and since current therapies cannot prevent OA progression, early detection and stratification of those at risk may enable effective pre-symptomatic interventions(7, 8). Several methods are used to define, diagnose and measure OA progression, including imaging techniques [e.g. plain radiography, Computed Tomography (CT) and Magnetic Resonance Imaging (MRI)]. Plain radiography provides high contrast and high resolution images for cortical and trabecular bone, but not for non-ossified structures (e.g. synovial fluid)(9). The most recognised radiographic measure classifying OA severity is Kellgren and Lawrence (KL) grading which assesses osteophytes, joint space narrowing (JSN), sclerosis and bone deformity(10, 11). However, it has been argued that MRI may be more suitable for imaging arthritic joints, providing a whole organ image of the joint(12). Whole-organ MRI scoring method (WORMS) is used in MRI for OA assessing damage, providing a detailed analysis of the joint.

Recently, OMERACT-OARSI (Outcome Measures in Rheumatology-Osteoarthritis Research Society International) have published a core domain set for clinical trials in hip and/or knee OA(13). Six domains were assessed as mandatory in the assessment of OA, including pain, physical function,

For peer review only - http://bmjopen.bmj.com/site/about/guidelines.xhtml

quality of life, patient's global assessment of the target joint, and adverse events including mortality and/or joint structure, depending on the intervention tested. However, there remains a need to identify risk factors for pain and structural damage in OA so that potential interventions can be studied in a timely manner. The purpose of this systematic review was therefore to identify risk factors for pain, worsening function and structural damage that can predict knee/hip OA development and progression. By identifying risk factors for OA pain and structural damage, tools for stratifying specific disease groups could be developed in the future.

METHODS

This systematic review has been reported in accordance with the PRISMA reporting guidelines. The review protocol was registered *a priori* through PROSPERO (Registration: CRD42018117643).

Search Strategy

A systematic search of the literature was undertaken from 1st January 1990 to 1st February 2020 using electronic databases: MEDLINE (Ovid), EMBASE (Ovid), MEDLINE, Web of Science and CINAHL (EBSCO). An example of the EMBASE search strategy of included search terms and Boolean operators is presented in **Supplementary File 1**. Unpublished literature databases including Clinicaltrials.gov, the WHO International Registry of Clinical Trials and OpenGrey were also searched.

Study Identification

Studies were eligible for inclusion if they were a full-text article that satisfied all of the following:

1) 100 or more participants analysed in the study (to increase power for comparisons);
2) convincing definition of OA using American College of Rheumatology criteria(14), based on symptoms of sustained pain and stiffness in the affected joint, radiographic changes

For peer review only

For peer review only - http://bmjopen.bmj.com/site/about/guidelines.xhtml

including osteophytes, cartilage loss, bone cysts/sclerosis and joint space narrowing, with normal inflammatory markers;

3) abstract/title that must refer to pain and/or structure in relation to OA as a primary disease;
4) Knee or hip OA;
5) pain and/or function scores;
6) joint imaged and
7) minimum six-month follow-up of pain/function outcome measures.

Non-English studies, letters, conference articles and reviews were excluded.

The titles and abstracts were reviewed by one reviewer (SS). The full-text for each paper was assessed for eligibility by one reviewer (SS) and double-checked by a second (TS). Any disagreements were addressed through discussion and adjudicated by a third reviewer (NS or FH). All studies which satisfied the criteria were included in the review.

Quality Assessment

To assess the risk of bias and the power of the methodology, the Downs & Black (D&B) tool was applied(15). These tools assessed the following aspects of each study: reporting quality, external validity, internal validity- bias, selection bias and power. The modified D&B tool was used. Accordingly, the 27-item randomised controlled trial (RCT) version was used for RCTs whilst the 18-item non-RCT version was used for non-RCT designs (**Supplementary File 2**). Both 18-item and 27-item tools have been demonstrated to be valid and reliable tools to assess RCT and non-RCT papers(14). Critical appraisal was performed by one reviewer (SS) and verified by a second (KT). Any disagreements were dealt with by discussion and adjudicated through a third reviewer (TS). In previous literature D&B score ranges were given corresponding quality: excellent (scored 26-28); good (scored 20-25); fair (scored 15-19); and poor (scored <14)(14). Item 4 on the non-RCT and Item 5 from the RCT tool are scored two points, hence the total scores equate to 19 and 28 points

For peer review only - http://bmjopen.bmj.com/site/about/guidelines.xhtml

respectively. The D&B tool was used to exclude poor quality studies with a score 15/28 or lower in RCTs and 10/19 or lower in non-RCTs.

Data Extraction

Data were extracted including: subject demographic data, study design, pain and function outcome measures, imaging used, OA severity scores, change in pain and function outcomes and change in OA severity scores. After all relevant data had been extracted, authors of these papers were approached to try and attain individual patient data (IPD) related to baseline and change in pain, function and structural scores for each study. No data was received from authors to inform this analysis.

Outcomes

The primary outcome was to determine the development of pain and functional impairment for those with knee and hip OA. The secondary outcome was to determine which factors are associated with structural changes in knee and hip OA.

Data Analysis

All data were assessed for study heterogeneity through scrutiny of the data extraction tables. These identified that there was minimum study-based heterogeneity based on: population, study design and interventions-exposure variabilities for given outcomes. Where there was study heterogeneity, a narrative analysis was undertaken. In this instance, the odds ratio (OR) of all predictor variables were tabulated with a range of OR presented. Where there was sufficient data to pool (two or more studies with data available to analyse) and study homogeneity evident, a pooled meta-analysis was deemed appropriate. As interpreted by the Cochrane Collaboration(16), when I^2 was 50% or greater representing high-statistical heterogeneity, a random-effects model meta-analysis was undertaken. When I^2 was less than this figure, a fixed effects model approach was adopted. Continuous

For peer review only - http://bmjopen.bmj.com/site/about/guidelines.xhtml

outcomes were assessed using mean difference (MD) scores of measures for developing severe OA, whereas dichotomous variables were assessed through OR data. All data were presented with 95% confidence intervals (CI) and forest-plots.

Due to the presentation of the data, there were minimal data to permit meta-analyses. Where there was insufficient data to pool the analysis (data only available from one study), a narrative analysis was undertaken to assess risk factors for the development of increased pain and functional impairment. Planned subgroup analyses included determine whether there was a difference in risk factors based on: (1) anatomical regions (i.e. difference between hip OA and knee OA); (2) geographical region. Analyses were undertaken on STATA version 14.0 (Stata Corp, Texas, USA) with forest plots constructed using RevMan Review Manager (RevMan) [Computer program]. Version 5.3. Copenhagen: The Nordic Cochrane Centre, The Cochrane Collaboration, 2014.)

Patient and Public Involvement: The research team acknowledges the assistance of both the OA tech network and Engineering and Physical Sciences Research Council. The authors also acknowledge receiving assistance from a meeting that enabled a consensus to be met on the eligibility criteria to be used, and this meeting consisted of the following people: Dr Angela Kedgley, Mrs Abiola Harrison, Professor Alan Boyde, Professor Alan Silman, Dr Amara Ezeonyeji, Miss Caroline Hing, Professor Cathy Holt, Ms Debbie Rolfe, Dr Enrica Papi, Ms Freija Ter Heegde, Mr Jingsong Wang, Dr John Garcia, Dr Mark Elliott, Professor Mary Sheppard, Miss Natasha Kapella, Mr Richard Rendle, Dr Shafaq Sikandar, Dr Sherif Hosny, Miss Soraia Silva, Miss Soraya Koushesh, Miss Susanna Cooper and Dr Thomas Barrick. No writing assistance was used.

For peer review only - http://bmjopen.bmj.com/site/about/guidelines.xhtml

RESULTS

Search Strategy

The results of the search strategy are presented in **Figure 1**. In total, 11,010 citations were identified. Of these, 141 papers were deemed potentially eligible and screened at full-text level. Of these, 82 met the selected criteria and were included(17-98).

Characteristics of Included Studies

A summary of the included studies is presented as **Table 1**. This consisted of 31 non-RCTs (27 observational cohort studies/four case-control studies) and 51 RCTs.

In total, 45,767 knees were included in the analysis. This consisted of 13,870 males and 23,497 females; four studies did not report the gender of their cohorts(17-20). Thirty-six studies were undertaken in the USA; 30 were undertaken in Europe; nine were conducted in Australasia and seven in Asia. Mean age of the cohorts was 61.7 years (standard deviation (SD): 7.56); 36 studies did not report age(17, 21, 22-54). Mean follow-up period was 35.4 months (SD: 33.6). The most common measures of pain were WOMAC pain (n=55; 50%) and Visual Analogue Scale (VAS) Pain (n=21; 19%). The most frequently used measures of function were WOMAC function (n=52; 44%), physical tests (n=16; 14%) and SF-36 (n=10; 9%).

Methodological Quality Assessment

The methodological quality of the evidence was moderate (**Supplementary File 2; Supplementary File 3**). Based on the results of the D&B non-RCT tool (31 studies; **Supplementary File 2**), recurrent strengths of the evidence were clear description of the participants recruited (29 studies; 94%), the representative nature that participants were to the population (31 studies; 100%), and variability in data presented for the main outcomes (31 studies; 100%). Furthermore, the main outcome measures were deemed reliable and valid in all studies (31 studies; 100%) with 89% (27 studies;

For peer review only - http://bmjopen.bmj.com/site/about/guidelines.xhtml

87%) studies adopting appropriate statistical analyses for their datasets. Recurrent limitations were not clearly reporting the main findings (20 studies; 65%), issues regarding the representation of the cohort from the wider public (18 studies; 58%) and only six studies (19%) basing their sample sizes on an *a prior* power calculation.

The results from the D&B RCT checklist (51 studies; **Supplementary File 3**) similarly reported findings with strength of the evidence around clear reporting of the cohort characteristics (49 studies; 96%) and interventions (50 studies; 98%), adoption of reliable/valid outcome measures (51 studies; 100%) and reported high compliance to study processes (37 studies; 73%). Recurrent weaknesses included recruiting cohorts which may not have been reflective of the wider population (19 studies; 37%), in clinic settings which may not have represented typical clinical practice (21 studies; 41%) and poorly adjusting for potential confounders in analyses (26 studies; 51%).

Knee OA

Narrative Review

Findings from the narrative analysis found the following were predictors for worsening joint pain: KL3 or 4 in women (OR: 11.3; 95% CI: 6.2 to 20.4), a WORMS lateral meniscal cyst (MC) score of 1 (OR: 4.3; 95% CI: 1.2 to 15.4), presence of chronic widespread pain (CWP) (OR: 3.2; 95% CI: 1.9 to 5.3), increase of ≥2 in WORMS BML score after 15 months (OR: 3.2; 95% CI: 1.5 to 6.8), meniscal maceration (OR: 2.8; 95% CI: 1.8 to 4.4) or damage ≥2 in WORMS (OR: 1.8; 95% CI: 0.9 to 3.6). We also found the following were the highest predictors of worsening function in people with knee OA: KL of <3 (OR: 3.3; 95% CI: 0.7 to 15.9), modified KL 3a (OR: 1.7; 95% CI: 0.7 to 3.8), modified KL 4a (OR: 1.5; 95% CI: 0.7 to 3.0), presence of osteophytes (OR: 1.3; 95% CI: 0.7 to 2.4), female gender (OR: 1.8 (95% CI: 1.1 to 3.0) to OR: 2.1 (95% CI: 1.2 to 3.5)), ethnicity (OR: 1.03; 95% CI 0.59 to 1.83) and synovitis ≥1 (OR: 1.3; 95% CI: 0.8 to 1.9).

For peer review only - http://bmjopen.bmj.com/site/about/guidelines.xhtml

Meta-Analysis

Two studies were identified where data could be evaluated for OA risk factors by meta-analysis (41,67). Three variables significantly associated with the development of knee OA. As illustrated in **Table 2** and **Figures 2a-d**, age (MD: 1.46, 95% CI: 0.26 to 2.66; p=0.02; N=823), KL of ≥ 2(MD: 2.04, 95% CI: 1.48 to 2.81; p<0.01; N=823) and knee effusion score ≥1 (OR: 1.35, 95% CI: 0.99 to 1.83: p=0.05; N=823) were all associated with the development of knee OA based on moderate quality evidence. The variables of gender and BMI were not shown to be significantly associated with the knee OA development (**Table 2**).

Due to the limited availability of data it was not possible to conduct the planned subgroup analyses to determine whether there was a difference in risk factors based on anatomical or geographical regions.

Hip OA

Narrative Analysis

This was based on low-quality evidence. There was no association between the development of hip BML and BMI or age. Predictors for worsening joint pain for people with hip OA included a large acetabular BML (OR: 5.2; 95% CI: 1.2 to 22.9), a large femoral head BML (OR: 4.4; 95% 1.4 to 19.7) with any large hip BML (OR: 4.4; 95% CI: 1.5 to 13.2), CWP (OR: 5.0; 95% CI: 2.8 to 9.1) and depression (OR: 1.9; 95% CI: 1.2 to 2.9). Baseline knee pain score (MD:-1.4; 95% CI: -1.6 to -1.2) and baseline hip pain score (MD:-0.7; 95% CI: -1.0 to -0.5) were significantly associated with the development of hip BMLs and pain.

Meta-Analysis

There were insufficient data to permit meta-analysis for the hip OA dataset.

For peer review only - http://bmjopen.bmj.com/site/about/guidelines.xhtml

DISCUSSION

Our systematic review and meta-analysis identified risk factors for knee and hip OA pain and structural damage based on evaluation of 82 studies. For the knee, increasing pain in knee OA was associated with KL grade 3 or 4 in women, WORMS lateral MC, presence of CWP, increase of ≥2 in WORMS BML score after 15 months and meniscal maceration. In addition, KL<3, KL 3a, KL 4a, osteophyte presence and female gender were associated with worsening function in people with knee OA. On meta-analysis, age, radiological features (KL score of 2 or more) and knee effusion were associated with development and/or progression of knee OA.

Our meta-analysis identified risk factors that are appreciated only when results were pooled together. These were namely WORMS-defined knee effusion score ≥1. To our knowledge, this is currently the largest and most up to date systematic review of its kind, reviewing 82 primary studies in 41,810 participants. Nonetheless, some risk factors from our meta-analysis have been recognised previously. For example, Silverwood *et al.* reported previous injuries are associated to developing knee OA, supporting the present analysis(95). Kingsbury *et al.* identified age and KL grade as predictive factors for developing knee OA, supporting the present findings(96). The meta-analyses provided both novel and supporting findings for risk factors associated with developing and progressing knee OA. A machine learning study assessed risk factors associated with pain and radiological progression in knee OA found that BMLs, osteophytes, medial meniscal extrusion, female gender and urine CTX-II contributed to progression(97). Nelson *et al's.* work is supported by other studies(95, 96). Therefore, the findings of our analysis support previous findings.

After plain radiography, MRI was the most used modality with WORMS as the commonest scoring reported for MRI. The MRI Osteoarthritis Knee Score (MOAKS)(99), expanded on WORMS by scoring entire sub-regions for BMLs rather than each BML, further division of cartilage regions and refined

For peer review only - http://bmjopen.bmj.com/site/about/guidelines.xhtml

the features assessed in meniscal morphology**.** Due to this progression from WORMS, having no MOAKS studies included in our final selection was surprising. This could be due to the eligibility criteria being too restrictive. A future systematic review and meta-analysis focusing on the imaging aspect of evaluating OA will be important. In hip OA, the evaluation of BML size and location is essential in predicting pain progression and these can be assessed effectively using MRI. We recommend that all MRI studies for hip OA evaluate BML size and location.

Gait analysis is considered a risk factor for pain/function and was therefore included as a target outcome measure. However, few studies included gait analysis measures, which could not be included in the analysis, perhaps due to the minimum sample size (n=100) being too restrictive.

There were several limitations within our study. Firstly, despite identifying novel risk factors for exhibiting knee OA, a small dataset was pooled together for the meta-analysis (two studies) compared to Silverwood *et al.* (34 studies)(93). This was particularly apparent for hip OA where only 12 studies assessed this population(8, 17, 23, 30, 46-48, 50, 54, 71, 76, 94). Consequently, the small dataset influenced the GRADE assessment that determined the evidence as low to moderate, restricting the strength of the associations of risk factors with OA development and progression. Further work may impact our confidence in the estimated effect, for both studies recruiting participants with hip and knee OA. Secondly, the eligibility criteria may have been too restrictive, resulting in limited papers including gait analysis or MOAKS. Wet biomarkers were not included in our analyses. Finally, the inability to pool data was partly attributed to variability in methods to report data. Standardising data collection and reporting is important in conducting meta-analyses. We believe the following should be undertaken to improve data pooling in future work: ensuring group comparisons in studies are selected from the same population (people with confirmed OA) to improve internal validity, observational studies should conduct a power analysis to determine

For peer review only - http://bmjopen.bmj.com/site/about/guidelines.xhtml

For peer review only

sample sizes and all studies should include absolute frequency of events data rather than summary odds ratios. Such considerations will improve future meta-analyses to identify OA risk factors.

To conclude, our work helps to develop steps towards building a stratification tool for risk factors for knee OA pain and structural damage development. We also highlight the need for collection of core datasets based on defined domains, that has recently also been highlighted by the OMERACT-OARSI core domain set for knee and hip OA(13). Collection of future datasets based on standardised core outcomes will assist in more robust identification of risk factors for large joint OA.

DECLARATIONS

Contributorship statement:
Conception and design: NS, FH, TS and SS Analysis and interpretation of the data: TS, SS and KT.
Drafting of the article: SS, TS, FH and NS.
Critical revision of the article: SS, TS, FH and NS Final approval of the article: SS, TS, FH and NS
Provision of study materials or patients: N/A.
Statistical expertise: TS.
Obtaining of funding: NS, FH and TS.
Administrative, technical, or logistic support: NS, TS and FH.
Collection and assembly of data: SS, TS and KT.

Data sharing statement: All data relevant to the study are included in the article or uploaded as supplementary information.

Ethics: No Ethical Approval was required for this study

Role of Funding Source: This study was funded by the Engineering and Physical Sciences Research Council (EPSRC) under the reference code 'EP/N027264/1' and The Wellcome Trust ISSF award to NS [Grant number 204809/Z/16/Z]. The funder had no input on the study design, data collection and analysis, manuscript preparation or the choice to submit it for publication.

For peer review only - http://bmjopen.bmj.com/site/about/guidelines.xhtml

Competing interests: None of the authors had any relation or contact with companies whose products or services may be related to the topic of the article.

REFERENCES

1. Cisternas M, Murphy L, Sacks, J, Solomon D, Pasta D and Helmick C. Alternative Methods for Defining Osteoarthritis and the Impact on Estimating Prevalence in a US Population-Based Survey. Arthritis Care & Research 2016; 68:574-580.
2. Cross M, Smith E, Hoy D, Nolte S, Ackerman I, Fransen M, Bridgett L, et al. The global burden of hip and knee osteoarthritis: estimates from the Global Burden of Disease 2010 study. Annals of the Rheumatic Diseases 2014; 73:1323-1330.
3. Wu Y, Goh E, Wang D and Ma S. Novel treatments for osteoarthritis: a recent update. Open Access Rheumatology: Research and Reviews 2018; 10:135-140.
4. Bellamy N, Buchanan WW, Goldsmith CH, Campbell J and Stitt LW. Validation study of WOMAC: a health status instrument for measuring clinically important patient relevant outcomes to antirheumatic drug therapy in patients with osteoarthritis of the hip or knee. J Rheumatol 1988; 15:1833-40.
5. Kraus V, Blanco F, Englund M, Karsdal M and Lohmander L. Call for standardized definitions of osteoarthritis and risk stratification for clinical trials and clinical use. Osteoarthritis and Cartilage 2015;23:1233-1241
6. Jin X, Jones G, Cicuttini F, Wluka A, Zhu Z, Han W, et al. Effect of Vitamin D Supplementation on Tibial Cartilage Volume and Knee Pain Among Patients With Symptomatic Knee Osteoarthritis: A Randomized Clinical Trial. JAMA 2016; 315:1005-13.
7. Hill CL, March LM, Aitken D, Lester SE, Battersby R, Hynes K, et al. Fish oil in knee osteoarthritis: a randomised clinical trial of low dose versus high dose. Ann Rheum Dis 2016; 75:23-9.

For peer review only - http://bmjopen.bmj.com/site/about/guidelines.xhtml

8. Maheu E, Cadet C, Marty M, Moyse D, Kerloch I, Coste P, et al. Randomised, controlled trial of avocado-soybean unsaponifiable (Piascledine) effect on structure modification in hip osteoarthritis: the ERADIAS study. Ann.Rheum.Dis 2014;73:376-84.
9. Peterfy C. Imaging Techniques. J Klippel, P Dieppe (Eds.), Rheumatology 2E, 1, Mosby, Philadelphia 1998; 1:14.1-14.18
10. Kellgren J and Lawrence J. Radiological Assessment of Osteo-Arthrosis. Annals of the Rheumatic Disease 1957; 16:494-502.
11. Schiphof D, Boers M and Bierma-Zeinstra SM. Differences in descriptions of Kellgren and Lawrence grades of knee osteoarthritis. Annals of the Rheumatic Disease 2008; 67:1034–1036.
12. Peterfy C, Guermazi A, Zaim S, Tirman P Miaux Y, White D, et al. Whole-Organ Magnetic Resonance Imaging Score (WORMS) of the knee in osteoarthritis. Osteoarthritis and Cartilage 2004;12:177-190.
13. Smith TO, Hawker GA, Hunter DJ. et al. The OMERACT-OARSI core domain set for measurement in clinical trials of hip and/or knee osteoarthritis. The Journal of Rheumatology 2019, 46(8): 981-989
14. Altman R, Asch E, Bloch D, Bole G, Borenstein D, Brandt K, et al. The American College of Rheumatology criteria for the classification and reporting of osteoarthritis of the knee. Arthritis Rheum 1986; 29: 1039-1049
15. Downs S and Black N. The feasibility of creating a checklist for the assessment of the methodological quality both of randomised and non-randomised studies of health care interventions. Journal of Epidemiology & Community Health 1998; 52:377-384.
16. Deeks JJ. Higgins JPT, Altman DG on behalf of the Cochrane Statistical Methods Group. Chapter 9: Analysing data and undertaking meta-analyses. In: Cochrane Handbook for Systematic Reviews of Interventions. Version 5.1.0 [update March 2011] Ed: Higgins JPT, Green S. The Cochrane Collaboration. Accessed: 02 April 2019. Available at: http://handbook-5-1.cochrane.org/

For peer review only - http://bmjopen.bmj.com/site/about/guidelines.xhtml

17. Valdes AM, Doherty SA, Zhang W, Muir KR, Maciewicz RA, Doherty M. Inverse relationship between preoperative radiographic severity and postoperative pain in patients with osteoarthritis who have undergone total joint arthroplasty. Seminars in Arthritis & Rheumatism 2012; 41:568-75.

18. Kinds MB, Marijnissen ACA, Vincken KL, Viergever MA, Drossaers-Bakker KW, Bijlsma JWJ, et al. Evaluation of separate quantitative radiographic features adds to the prediction of incident radiographic osteoarthritis in individuals with recent onset of knee pain: 5-year follow-up in the CHECK cohort. 2012; 20:548-56.

19. Davis J, Eaton CB, Lo GH, Lu B, Price LL, McAlindon TE, et al. Knee symptoms among adults at risk for accelerated knee osteoarthritis: data from the Osteoarthritis Initiative. Clin.Rheumatol 2017;36:1083-9.

20. Akelman MR, Fadale PD, Hulstyn MJ, Shalvoy RM, Garcia A, Chin KE, et al. Effect of Matching or Overconstraining Knee Laxity During Anterior Cruciate Ligament Reconstruction on Knee Osteoarthritis and Clinical Outcomes: A Randomized Controlled Trial With 84-Month Follow-up. Am.J.Sports Med 2016; 44:1660-70.

21. Yu SP, Williams M, Eyles JP, Chen JS, Makovey J, Hunter DJ. Effectiveness of knee bracing in osteoarthritis: pragmatic trial in a multidisciplinary clinic. Int.J.Rheum.Dis. 2016;19:279-86.

22. Urish KL, Keffalas MG, Durkin JR, Miller DJ, Chu CR, Mosher TJ. T2 texture index of cartilage can predict early symptomatic OA progression: data from the osteoarthritis initiative. Osteoarthritis & Cartilage 2013; 21:1550-7.

23. Rozendaal RM, Koes BW, van Osch GJ, Uitterlinden EJ, Garling EH, Willemsen SP, et al. Effect of glucosamine sulfate on hip osteoarthritis: a randomized trial. Ann.Intern.Med 2008; 148:268-77.

24. Roman-Blas JA, Castaneda S, Sanchez-Pernaute O, Largo R, Herrero-Beaumont G, CS/GS Combined Therapy Study Group. Combined Treatment With Chondroitin Sulfate and Glucosamine Sulfate Shows No Superiority Over Placebo for Reduction of Joint Pain and

For peer review only - http://bmjopen.bmj.com/site/about/guidelines.xhtml

Functional Impairment in Patients With Knee Osteoarthritis: A Six-Month Multicenter, Randomized, Double-Blind, Placebo-Controlled Clinical Trial. Arthritis Rheumatol 2017; 69:77-85.

25. Riddle DL and Jiranek WA. Knee osteoarthritis radiographic progression and associations with pain and function prior to knee arthroplasty: a multicenter comparative cohort study. Osteoarthritis and Cartilage 2015; 23:391-6

26. Reginster JY, Deroisy R, Rovati LC, Lee RL, Lejeune E, Bruyere O, et al. Long-term effects of glucosamine sulphate on osteoarthritis progression: a randomised, placebo-controlled clinical trial. Lancet 2001;357 North American Edition:251-6.

27. Raynauld JP, Martel-Pelletier J, Haraoui B, Choquette D, Dorais M, Wildi LM, et al. Risk factors predictive of joint replacement in a 2-year multicentre clinical trial in knee osteoarthritis using MRI: results from over 6 years of observation. Ann.Rheum.Dis 2011; 70:1382-8.

28. Podsiadlo P., Cicuttini F.M., Wolski M., Stachowiak G.W., Wluka AE. Trabecular bone texture detected by plain radiography is associated with an increased risk of knee replacement in patients with osteoarthritis: A 6 year prospective follow up study. Osteoarthritis and Cartilage 2014; 22:71-5.

29. Pham T, Le Henanff A, Ravaud P, Dieppe P, Paolozzi L, Dougados M. Evaluation of the symptomatic and structural efficacy of a new hyaluronic acid compound, NRD101, in comparison with diacerein and placebo in a 1 year randomised controlled study in symptomatic knee osteoarthritis. Ann.Rheum.Dis 2004; 63:1611-7.

30. Pavelka K., Gatterova J., Gollerova V., Urbanova Z., Sedlackova M., Altman RD. A 5-year randomized controlled, double-blind study of glycosaminoglycan polysulphuric acid complex (Rumalon) as a structure modifying therapy in osteoarthritis of the hip and knee. Osteoarthritis and Cartilage 2000; 8:335-42.

For peer review only - http://bmjopen.bmj.com/site/about/guidelines.xhtml

31. Michel BA, Stucki G, Frey D, De Vathaire F, Vignon E, Bruehlmann P, et al. Chondroitins 4 and 6 sulfate in osteoarthritis of the knee: a randomized, controlled trial. Arthritis Rheum 2005; 52:779-86.

32. Messier SP, Loeser RF, Miller GD, Morgan TM, Rejeski WJ, Sevick MA, et al. Exercise and dietary weight loss in overweight and obese older adults with knee osteoarthritis: the Arthritis, Diet, and Activity Promotion Trial. Arthritis Rheum 2004; 50:1501-10.

33. McAlindon T, LaValley M, Schneider E, Nuite M, Lee JY, Price LL, et al. Effect of vitamin D supplementation on progression of knee pain and cartilage volume loss in patients with symptomatic osteoarthritis: a randomized controlled trial. JAMA 2013;309:155-62.

34. Marsh JD, Birmingham TB, Giffin JR, Isaranuwatchai W, Hoch JS, Feagan BG, et al. Cost-effectiveness analysis of arthroscopic surgery compared with non-operative management for osteoarthritis of the knee. BMJ Open 2016; 6:e009949,2015-009949.

35. Lohmander LS, Hellot S, Dreher D, Krantz EF, Kruger DS, Guermazi A, et al. Intraarticular sprifermin (recombinant human fibroblast growth factor 18) in knee osteoarthritis: a randomized, double-blind, placebo-controlled trial. Arthritis Rheumatol 2014; 66:1820-31.

36. Kongtharvonskul J, Woratanarat P, McEvoy M, Attia J, Wongsak S, Kawinwonggowit V, et al. Efficacy of glucosamine plus diacerein versus monotherapy of glucosamine: a double-blind, parallel randomized clinical trial. Arthritis Res.Ther 2016; 18:233,016-1124-9.

37. Katz JN, Brophy RH, Chaisson CE, de Chaves L, Cole BJ, Dahm DL, et al. Surgery versus physical therapy for a meniscal tear and osteoarthritis. N.Engl.J.Med 2013;368:1675-84.

38. Karsdal MA, Byrjalsen I, Alexandersen P, Bihlet A, Andersen JR, Riis BJ, et al. Treatment of symptomatic knee osteoarthritis with oral salmon calcitonin: results from two phase 3 trials. Osteoarthritis and Cartilage 2015; 23:532-43.

39. Housman L, Arden N, Schnitzer TJ, Birbara C, Conrozier T, Skrepnik N, et al. Intra-articular hylastan versus steroid for knee osteoarthritis. Knee Surg.Sports Traumatol. Arthrosc 2014;22:1684-92.

For peer review only - http://bmjopen.bmj.com/site/about/guidelines.xhtml

40. Henriksen M., Hunter D.J., Dam E.B., Messier S.P., Andriacchi T.P., Lohmander L.S., et al. Is increased joint loading detrimental to obese patients with knee osteoarthritis? A secondary data analysis from a randomized trial. Osteoarthritis and Cartilage 2013; 21:1865-75.

41. Guermazi A., Hayashi D., Roemer F.W., Niu J., Yang M., Lynch J.A., et al. Cyst-like lesions of the knee joint and their relation to incident knee pain and development of radiographic osteoarthritis: The MOST study. Osteoarthritis and Cartilage 2010; 18:1386-92.

42. Glass NA, Torner JC, Frey Law LA, Wang K, Yang T, Nevitt MC, et al. The relationship between quadriceps muscle weakness and worsening of knee pain in the MOST cohort: a 5-year longitudinal study. Osteoarthritis and Cartilage 2013; 21:1154-9.

43. Filardo G, Di Matteo B, Di Martino A, Merli ML, Cenacchi A, Fornasari P, et al. Platelet-Rich Plasma Intra-articular Knee Injections Show No Superiority Versus Viscosupplementation: A Randomized Controlled Trial. Am.J.Sports Med 2015;43:1575-82.

44. Ettinger WH,Jr, Burns R, Messier SP, Applegate W, Rejeski WJ, Morgan T, et al. A randomized trial comparing aerobic exercise and resistance exercise with a health education program in older adults with knee osteoarthritis. The Fitness Arthritis and Seniors Trial (FAST). JAMA 1997; 277:25-31.

45. Eckstein F, Hitzl W, Duryea J, Kent Kwoh C, Wirth W, OAI investigators. Baseline and longitudinal change in isometric muscle strength prior to radiographic progression in osteoarthritic and pre-osteoarthritic knees--data from the Osteoarthritis Initiative. Arthritis & rheumatology 2013; 21:682-90.

46. Dougados M, Nguyen M, Berdah L, Mazieres B, Vignon E, Lequesne M, et al. Evaluation of the structure-modifying effects of diacerein in hip osteoarthritis: ECHODIAH, a three-year, placebo-controlled trial. Evaluation of the Chondromodulating Effect of Diacerein in OA of the Hip. Arthritis Rheum 2001; 44:2539-47

47. Chandrasekaran S, Gui C, Darwish N, Lodhia P, Suarez-Ahedo C, Domb BG. Outcomes of Hip Arthroscopic Surgery in Patients With Tonnis Grade 1 Osteoarthritis With a Minimum 2-Year

For peer review only - http://bmjopen.bmj.com/site/about/guidelines.xhtml

Follow-up: Evaluation Using a Matched-Pair Analysis With a Control Group With Tonnis Grade 0. Am.J.Sports Med 2016;44:1781-8.

48. Chandrasekaran S, Darwish N, Gui C, Lodhia P, Suarez-Ahedo C, Domb BG. Outcomes of Hip Arthroscopy in Patients with Tonnis Grade-2 Osteoarthritis at a Mean 2-Year Follow-up: Evaluation Using a Matched-Pair Analysis with Tonnis Grade-0 and Grade-1 Cohorts. Journal of Bone & Joint Surgery 2016; 98:973-82.

49. Campbell DG, Duncan WW, Ashworth M, Mintz A, Stirling J, Wakefield L, et al. Patellar resurfacing in total knee replacement: a ten-year randomised prospective trial. J.Bone Joint Surg.Br 2006;88:734-9.

50. Brown MT, Murphy FT, Radin DM, Davignon I, Smith MD, West CR. Tanezumab reduces osteoarthritic hip pain: results of a randomized, double-blind, placebo-controlled phase III trial. Arthritis Rheum 2013; 65:1795-803.

51. Brown MT, Murphy FT, Radin DM, Davignon I, Smith MD, West CR. Tanezumab reduces osteoarthritic knee pain: results of a randomized, double-blind, placebo-controlled phase III trial. J.Pain 2012; 13:790-8.

52. Bisicchia S, Bernardi G, Tudisco C. HYADD 4 versus methylprednisolone acetate in symptomatic knee osteoarthritis: a single-centre single blind prospective randomised controlled clinical study with 1-year follow-up. Clin.Exp.Rheumatol 2016;34:857-63.

53. Bingham CO,3rd, Buckland-Wright JC, Garnero P, Cohen SB, Dougados M, Adami S, et al. Risedronate decreases biochemical markers of cartilage degradation but does not decrease symptoms or slow radiographic progression in patients with medial compartment osteoarthritis of the knee: results of the two-year multinational knee osteoarthritis structural arthritis study. Arthritis Rheum 2006; 54:3494-507.

54. Ahedi H, Aitken D, Blizzard L, Cicuttini F, Jones G. A population-based study of the association between hip bone marrow lesions, high cartilage signal, and hip and knee pain. Clin.Rheumatol 2014;33:369-76.

For peer review only - http://bmjopen.bmj.com/site/about/guidelines.xhtml

55. Amin S, Guermazi A, Lavalley MP, Niu J, Clancy M, Hunter DJ, et al. Complete anterior cruciate ligament tear and the risk for cartilage loss and progression of symptoms in men and women with knee osteoarthritis. Osteoarthritis Cartilage 2008; 16:897-902

56. Antony B, Driban JB, Price LL, Lo GH, Ward RJ, Nevitt M, et al. The relationship between meniscal pathology and osteoarthritis depends on the type of meniscal damage visible on magnetic resonance images: data from the Osteoarthritis Initiative. Osteoarthritis Cartilage 2017; 25:76-84.

57. Arden NK, Cro S, Sheard S, Dore CJ, Bara A, Tebbs SA, et al. The effect of vitamin D supplementation on knee osteoarthritis, the VIDEO study: a randomised controlled trial. Osteoarthritis Cartilage 2016; 24:1858-66.

58. Ayral X, Mackillop N, Genant HK, Kirkpatrick J, Beaulieu A, Pippingskiold P, et al. Arthroscopic evaluation of potential structure-modifying drug in osteoarthritis of the knee. A multicenter, randomized, double-blind comparison of tenidap sodium vs piroxicam. Osteoarthritis Cartilage 2003;11:198-207.

59. Baselga Garcia-Escudero J and Miguel Hernandez Trillos P. Treatment of Osteoarthritis of the Knee with a Combination of Autologous Conditioned Serum and Physiotherapy: A Two-Year Observational Study. 2015;10:e0145551.

60. Bevers K, Vriezekolk JE, Bijlsma JWJ, van den Ende,Cornelia H M., den Broeder AA. Ultrasonographic predictors for clinical and radiological progression in knee osteoarthritis after 2 years of follow-up. Rheumatology 2015;54:2000-3.

61. Birmingham T.B., Giffin J.R., Chesworth B.M., Bryant D.M., Litchfield R.B., Willits K., et al. Medial opening wedge high tibial osteotomy: A prospective cohort study of gait, radiographic, and patient-reported outcomes. Arthritis Care and Research 2009;61:648-57.

62. Brandt KD, Mazzuca SA, Katz BP, Lane KA, Buckwalter KA, Yocum DE, et al. Effects of doxycycline on progression of osteoarthritis: results of a randomized, placebo-controlled, double-blind trial. Arthritis Rheum 2005;52:2015-25.

For peer review only - http://bmjopen.bmj.com/site/about/guidelines.xhtml

63. Bruyere O, Pavelka K, Rovati LC, Deroisy R, Olejarova M, Gatterova J, et al. Glucosamine sulfate reduces osteoarthritis progression in postmenopausal women with knee osteoarthritis: evidence from two 3-year studies. Menopause 2004;11:138-43.

64. Conrozier T, Eymard F, Afif N, Balblanc JC, Legre-Boyer V, Chevalier X, et al. Safety and efficacy of intra-articular injections of a combination of hyaluronic acid and mannitol (HAnOX-M) in patients with symptomatic knee osteoarthritis: Results of a double-blind, controlled, multicenter, randomized trial. Knee 2016; 23:842-8.

65. Dowsey M.M., Nikpour M., Dieppe P., Choong PFM. Associations between pre-operative radiographic changes and outcomes after total knee joint replacement for osteoarthritis. Osteoarthritis and Cartilage 2012; 20:1095-102.

66. Felson DT, Niu J, Yang T, Torner J, Lewis CE, Aliabadi P, et al. Physical activity, alignment and knee osteoarthritis: data from MOST and the OAI. Osteoarthritis and Cartilage 2013; 21:789-95.

67. Felson DT, Niu J, Guermazi A, Roemer F, Aliabadi P, Clancy M, et al. Correlation of the development of knee pain with enlarging bone marrow lesions on magnetic resonance imaging. Arthritis Rheum 2007; 56:2986-92.

68. Hamilton TW, Pandit HG, Maurer DG, Ostlere SJ, Jenkins C, Mellon SJ, et al. Anterior knee pain and evidence of osteoarthritis of the patellofemoral joint should not be considered contraindications to mobile-bearing unicompartmental knee arthroplasty: a 15-year follow-up. Bone & Joint Journal 2017; 99-B:632-9.

69. Hellio le Graverand MP, Clemmer RS, Redifer P, Brunell RM, Hayes CW, Brandt KD, et al. A 2-year randomised, double-blind, placebo-controlled, multicentre study of oral selective iNOS inhibitor, cindunistat (SD-6010), in patients with symptomatic osteoarthritis of the knee. Ann.Rheum.Dis 2013; 72:187-95.

70. Hochberg MC, Martel-Pelletier J, Monfort J, Moller I, Castillo JR, Arden N, et al. Combined chondroitin sulfate and glucosamine for painful knee osteoarthritis: a multicentre,

For peer review only - http://bmjopen.bmj.com/site/about/guidelines.xhtml

For peer review only

randomised, double-blind, non-inferiority trial versus celecoxib. Ann.Rheum.Dis 2016; 75:37-44.

71. Hoeksma HL, Dekker J, Ronday HK, Heering A, van der Lubbe N, Vel C, et al. Comparison of manual therapy and exercise therapy in osteoarthritis of the hip: a randomized clinical trial. Arthritis Rheum 2004; 51:722-9.

72. Huang MH, Lin YS, Yang RC, Lee CL. A comparison of various therapeutic exercises on the functional status of patients with knee osteoarthritis. Semin.Arthritis Rheum 2003; 32:398-406.

73. Huizinga MR, Gorter J, Demmer A, Bierma-Zeinstra SMA, Brouwer RW. Progression of medial compartmental osteoarthritis 2-8 years after lateral closing-wedge high tibial osteotomy. Knee Surgery, Sports Traumatology, Arthroscopy 2017; 25:3679-86.

74. Kahn T.L., Soheili A., Schwarzkopf R. Outcomes of Total Knee Arthroplasty in Relation to Preoperative Patient-Reported and Radiographic Measures: Data From the Osteoarthritis Initiative. Geriatric Orthopaedic Surgery and Rehabilitation 2013; 4:117-26.

75. Kim YH, Park JW, Kim JS. The Clinical Outcome of Computer-Navigated Compared with Conventional Knee Arthroplasty in the Same Patients: A Prospective, Randomized, Double-Blind, Long-Term Study. J.Bone Joint Surg.Am 2017; 99:989-96.

76. Lequesne M, Maheu E, Cadet C, Dreiser R. Structural effect of avocado/soybean unsaponifiables on joint space loss in osteoarthritis of the hip. Arthritis & Rheumatism: Arthritis Care & Research 2002; 47:50-8

77. Messier SP, Gutekunst DJ, Davis C, DeVita P. Weight loss reduces knee-joint loads in overweight and obese older adults with knee osteoarthritis. Arthritis Rheum 2005; 52:2026-32.

78. Messier SP, Mihalko SL, Legault C, Miller GD, Nicklas BJ, DeVita P, et al. Effects of intensive diet and exercise on knee joint loads, inflammation, and clinical outcomes among

For peer review only

For peer review only - http://bmjopen.bmj.com/site/about/guidelines.xhtml

overweight and obese adults with knee osteoarthritis: the IDEA randomized clinical trial. JAMA 2013;310: 1263-73.

79. Muraki S., Akune T., Nagata K., Ishimoto Y., Yoshida M., Tokimura F., et al. Association of knee osteoarthritis with onset and resolution of pain and physical functional disability: The ROAD study. Modern Rheumatology 2014; 24:966-73.

80. Muraki S, Akune T, Nagata K, Ishimoto Y, Yoshida M, Tokimura F, et al. Does osteophytosis at the knee predict health-related quality of life decline? A 3-year follow-up of the ROAD study. Clin.Rheumatol 2015; 34:1589-97.

81. Pavelka K, Gatterova J, Olejarova M, Machacek S, Giacovelli G, Rovati LC. Glucosamine sulfate use and delay of progression of knee osteoarthritis: a 3-year, randomized, placebo-controlled, double-blind study. Arch.Intern.Med 2002; 162:2113-23.

82. Rat A, Baumann C, Guillemin F. National, multicentre, prospective study of quality of life in patients with osteoarthritis of the knee treated with hylane G-F 20. Clin.Rheumatol 2011; 30:1285-93.

83. Reginster JY, Badurski J, Bellamy N, Bensen W, Chapurlat R, Chevalier X, et al. Efficacy and safety of strontium ranelate in the treatment of knee osteoarthritis: results of a double-blind, randomised placebo-controlled trial. Ann.Rheum.Dis 2013; 72:179-86.

84. Romagnoli S. and Marullo M. Mid-Term Clinical, Functional, and Radiographic Outcomes of 105 Gender-Specific Patellofemoral Arthroplasties, With or Without the Association of Medial Unicompartmental Knee Arthroplasty. J.Arthroplasty 2017; 33:688-695

85. Sanchez-Ramirez DC, van der Leeden M, van der Esch M, Roorda LD, Verschueren S, van Dieen J, et al. Increased knee muscle strength is associated with decreased activity limitations in established knee osteoarthritis: Two-year follow-up study in the Amsterdam osteoarthritis cohort. J.Rehabil.Med 2015; 47:647-54.

86. Sawitzke AD, Shi H, Finco MF, Dunlop DD, Harris CL, Singer NG, et al. Clinical efficacy and safety of glucosamine, chondroitin sulphate, their combination, celecoxib or placebo taken

For peer review only - http://bmjopen.bmj.com/site/about/guidelines.xhtml

to treat osteoarthritis of the knee: 2-year results from GAIT. Ann.Rheum.Dis 2010; 69:1459-64.

87. Skou ST, Wise BL, Lewis CE, Felson D, Nevitt M, Segal NA, et al. Muscle strength, physical performance and physical activity as predictors of future knee replacement: a prospective cohort study. Osteoarthritis & Cartilage 2016; 24:1350-6.

88. Sowers M, Karvonen-Gutierrez CA, Jacobson JA, Jiang Y, Yosef M. Associations of anatomical measures from MRI with radiographically defined knee osteoarthritis score, pain, and physical functioning. Journal of Bone & Joint Surgery 2011; 93:241-51.

89. Spector TD, Conaghan PG, Buckland-Wright JC, Garnero P, Cline GA, Beary JF, et al. Effect of risedronate on joint structure and symptoms of knee osteoarthritis: results of the BRISK randomized, controlled trial [ISRCTN01928173. Arthritis Res.Ther 2005; 7:R625-33.

90. Sun SF, Hsu CW, Lin HS, Liou IH, Chen YH, Hung CL. Comparison of Single Intra-Articular Injection of Novel Hyaluronan (HYA-JOINT Plus) with Synvisc-One for Knee Osteoarthritis: A Randomized, Controlled, Double-Blind Trial of Efficacy and Safety. J.Bone Joint Surg.Am 2017; 99:462-71.

91. Weng MC, Lee CL, Chen CH, Hsu JJ, Lee WD, Huang MH, et al. Effects of different stretching techniques on the outcomes of isokinetic exercise in patients with knee osteoarthritis. Kaohsiung J.Med.Sci 2009; 25:306-15.

92. White DK, Neogi T, Nguyen UDT, Niu J, Zhang Y. Trajectories of functional decline in knee osteoarthritis: the Osteoarthritis Initiative. Rheumatology 2016; 55:801-8.

93. Witt C, Brinkhaus B, Jena S, Linde K, Streng A, Wagenpfeil S, et al. Acupuncture in patients with osteoarthritis of the knee: a randomised trial. Lancet 2005; 366:136-43.

94. Yusuf E, Bijsterbosch J, Slagboom PE, Kroon HM, Rosendaal FR, Huizinga TWJ, et al. Association between several clinical and radiological determinants with long-term clinical progression and good prognosis of lower limb osteoarthritis. PLoS ONE 2011; 6:e25426.

For peer review only - http://bmjopen.bmj.com/site/about/guidelines.xhtml

95. Silverwood V, Blagojevic-Bucknall M, Jinks C, Jordan J, Protheroe J and Jordan K. Current evidence on risk factors for knee osteoarthritis in older adults: a systematic review and meta-analysis. Osteoarthritis and Cartilage 2015; 23:507-515.

96. Kingsbury S, Corp N, Watt F, Felson D, O'Neill T, Holt C, et al. Harmonising data collection from osteoarthritis studies to enable stratification: recommendations on core data collection from an Arthritis Research UK clinical studies group. Rheumatology 2016; 55:1394-1402.

97. Nelson A, Fang F, Arbeeva L, Cleveland, R, Schwartz T, Callahan L et al. A machine learning approach to knee osteoarthritis phenotyping: data from the FNIH Biomarkers Consortium. Osteoarthritis and Cartilage 2019; 27:994-1001.

98. Van der Esch M, van der Leeden M, Roorda LD, Lems WF, Dekker J. Predictors of self-reported knee instability among patients with knee osteoarthritis: results of the Amsterdam osteoarthritis cohort. Clin Rheumatol. 2016; 35(12): 3007-13

99. Hunter D, Guermazi A, Lo G, Grainger A, Conaghan P, Boudreau R, et al. Evolution of semi-quantitative whole joint assessment of knee OA: MOAKS (MRI Osteoarthritis Knee Score). Osteoarthritis and Cartilage 2011; 19:990-1002.

For peer review only

Table 1: Characteristics of included studies

	Study Design	Number joints (hip/knees)	Gender (male:female)	Country origin	Mean age (years)	Follow-up duration (months)	Pain outcome measures	Functional outcome measures
Ahedi 2014 (54)	Observational cohort	198 hips	111:87	Australia	UTD	132	WOMAC Pain	NA
Akelman 2016 (20)	RCT	107 knee	UTD	USA	23.5	84	KOOS pain; SF-36 Body pain	SF-36 Physical; AP laxity; IKDC2000
Amin 2008 (55)	Observational cohort	265 knees	152:113	USA	67	30	VAS Pain	WOMAC Function
Antony 2017 (56)	Observational cohort	463 knees	245:218	USA	63	24	WOMAC Pain	NA
Arden 2016 (57)	RCT	474 knees	185:289	UK	64	36	WOMAC Pain	WOMAC Function
Ayral 2003 (58)	RCT	665 knees	259:406	Australia, Belgium, Canada, Denmark, Finland, France, Hungary, Norway, Spain, United Kingdom U.S.A.	61.3	12	WOMAC Pain	WOMAC Function
Baselga Garcia-Escudero 2015 (59)	Observational cohort	118 knees	43:75	Spain	59.1	24	NRS; WOMAC Pain	WOMAC Function
Bevers 2015 (60)	Observational cohort	125 knees	57:68	Netherlands	57	24	WOMAC Pain	WOMAC Function
Bingham 2006 (53)	RCT	2483 knees	735:1748	USA Canada Austria Czech Republic France Germany Hungary Ireland Italy Netherlands Poland Croatia	UTD	24	WOMAC Pain	WOMAC Function
Birmingham 2009 (61)	Observational cohort	126 knees	100:26	Canada	47.5	24	KOOS Pain	KOOS Function; SF-36

For peer review only - http://bmjopen.bmj.com/site/about/guidelines.xhtml

								Physical; LEFS
Bisicchia 2016 (52)	RCT	150 knees	47:103	Italy	UTD	12	VAS Pain; SF-36	SF-36
Brandt 2005 (62)	RCT	431 knees	0:431	USA	54.9	30	WOMAC Pain; VAS Pain	WOMAC Function
Brown 2012 (51)	RCT	690 knees	270:420	USA	UTD	32 weeks	WOMAC Pain; NRS weekly pain	WOMAC Function; SF-36 Function
Brown 2013 (50)	RCT	621 hips	237:384	USA	UTD	32 weeks	WOMAC Pain	WOMAC Function
Bruyere 2004 (63)	RCT	319 knee	0:319	Belgium	64.0	36	WOMAC Pain	WOMAC Function
Campbell 2006 (49)	RCT	100 knees	28:72	Australia	UTD	120	American Knee Society Score; WOMAC Pain	American Knee Society Score (function); WOMAC Function
Chandraseka ran 2016A (48)	Case-Control	111 hips	66:45	USA	UTD	24	Modified Harris Hip Score; Nonarthr itic hip score; VAS Pin	Modified Harris Hip Score; Nonarthr itic hip score; Hip Outcome Score; Sports & ADLs
Chandraseka ran 2016B (47)	Case-Control	186 hips	96:90	USA	UTD	24	Modified Harris Hip Score; Nonarthr itic hip score; VAS Pin	Modified Harris Hip Score; Nonarthr itic hip score; Hip Outcome Score; Sports & ADLs
Conrozier 2016 (64)	RCT	205 knees	88:117	France	65	26	WOMAC Pain; NRS walking pain	WOMAC Function
Davis 2017 (19)	Case-control	3132 knees	UTD	USA	UTD	48	WOMAC Pain; KOOS Pain	WOMAC Function

For peer review only - http://bmjopen.bmj.com/site/about/guidelines.xhtml

Dougados 2001 (46)	RCT	507 hips	202:305	France	UTD	36	VAS Pain	Lequesne Index
Dowsey 2012 (65)	Observational cohort	478 knees	147:331	Australia	70.8	24	IKSS Pain	IKSS Function
Eckstein 2013 (45)	RCT	1412 knees	611:801	Austria	UTD	48	WOMAC Pain	NA
Ettinger 1997 (44)	RCT	439 knees	131:308	USA	UTD	18	Pain intensity score	Physical Test
Felson 2013 (66)	Observational cohort	3498 knees	867:1206	USA	61.2	30	WOMAC Pain	PASE
Felson 2007 (67)	Observational cohort	330 knees	111:2111	USA	62.1	15	NA	Quadriceps strength (N)
Filardo 2015 (43)	RCT	183 knees	112:71	Italy	UTD	48	KOOS Pain; IKDC	KOOS Function; Tegner; IKDC
Glass 2013 (42)	Observational cohort	4648 knees	918:1486	USA	UTD	24	WOMAC Pain; NRS Pain	WOMAC Function
Guermazi 2010 (41)	Case-control	493 knees	185:308	USA	UTD	60	WOMAC Pain	PASE
Hamilton 2017 (68)	Observational cohort	805 knees	416:289	UK	66	30	WOMAC Pain	WOMAC Function
Hellio le Graverand 2013 (69)	RCT	1457 knees	343:1114	USA Canada Australia, Belgium, Czech Republic, Germany, Hungary, Italy, Poland, Russian Federation, Slovakia, Spain, Argentina Peru	61.0	180	Oxford Knee Score	Oxford Knee Score; American Knee Society Score; Tegner
Henriksen 2013 (40)	RCT	157 knees	28:129	Denmark	UTD	24	WOMAC Pain	WOMAC Function
Hill 2016 (5)	RCT	202 knees	102:100	Australia	61	12	KOO Pain	KOOS Function and kinematic assessment
Hochberg 2016 (70)	RCT	522 knees	84:438	France Germany	62.7	24	WOMAC Pain	WOMAC Function

For peer review only - http://bmjopen.bmj.com/site/about/guidelines.xhtml

				Poland Spain				
Hoeksma 2004 (71)	RCT	109 hips	33:76	Netherlands	72	6	WOMAC Pain; Huskisson's VAS; EQ-5D Pain	WOMAC Function; EQ-5D Function
Housman 2014 (39)	RCT	391 knees	130:261	USA Canada France UK Germany	UTD	6	SF-36 Body Pain; Harris Hip Score; VAS Pain	SF-36 Function; Harris Hip Score; ROM
Huang 2003 (72)	RCT	264 knees	39:93	Taiwan	62	6	WOMAC Pain	NA
Huizinga 2017 (73)	Observational cohort	298 knees	201:97	Netherlands	51	12	VAS Pain	Lequesne index; Walking speed
Jin 2016 (6)	RCT	413 knees	205:208	Australia	63.2	24	WOMAC Pain; VAS Pain	WOMAC Function
Kahn 2013 (74)	Observational cohort	174 knees	70:102	USA	67.0	6	WOMAC Pain	WOMAC Function
Karsdal 2015 (38)	RCT	2207 knees	773:1424	Denmark	UTD	24	WOMAC Pain	WOMAC Function
Katz 2013 (37)	RCT	330 knees	143:187	USA	UTD	12	KOO Pain	WOMAC Function; SF-36 Function
Kim 2017 (75)	RCT	352 knees	9:153	Republic of Korea	68.1	144	WOMAC	Knee Society Knee Score Function; ROM; UCLA Activity
Kinds 2012 (18)	RCT	565 knees	UTD	Netherlands	UTD	60	WOMAC Pain	WOMAC Function
Kongtharvonskul 2016 (36)	RCT	148 knees	25:123	Thailand	UTD	6	WOMAC Pain; VAS Pain	WOMAC Function
Lequesne 2002 (76)	RCT	163 hips	102:61	France	63.2	24	VAS Pain	Lequesne Index
Lohmander 2014 (35)	RCT	170 knees	52:116	Bulgaria Canada Croatia Finland Germany Poland Serbia Africa Sweden	UTD	12	WOMAC Pain	WOMAC Function

For peer review only - http://bmjopen.bmj.com/site/about/guidelines.xhtml

				USA				
Maheu 2014 (8)	RCT	345 hips	159:186	France	62.2	36	WOMAC Pain; Global Hip Pain	Lequesne Index; WOMAC Function; Global handicap NRS
Marsh 2016 (34)	RCT	168 knees	57:112	Canada	UTD	24	WOMAC	WOMAC
McAlindion 2013 (33)	RCT	146 knees	57:89	USA	UTD	24	WOMAC Pain	WOMAC Function; Physical Test
Messier 2004 (32)	RCT	316 knees	89:227	USA	UTD	18	WOMAC Pain	WOMAC Function; Physical Test
Messier 2005 (77)	RCT	142 knees	37:105	USA	68.5	18	WOMAC Pain	WOMAC Function; Physical Test
Messier 2013 (78)	RCT	454 knees	128:325	USA	66	18	WOMAC Pain	WOMAC Function; Physical Test; SF-36 Physical
Michel 2005 (31)	RCT	300 knees	146:154	Switzerland	UTD	24	WOMAC Pain	WOMAC Function; Physical Test
Muraki 2014 (79)	Observational cohort	1558 knees	553:1005	Japan	67.0	40	WOMAC Pain	WOMAC Function;
Muraki 2015 (80)	Observational cohort	1525 knees	546:979	Japan	67.0	40	WOMAC Pain	WOMAC Function
Pavelka 2000 (30)	RCT	277 knees; 117 hips	109:285	Czech Republic	58	60	NA	Lequesne Index
Pavelka 2002 (81)	RCT	202 knees	45:157	Czech Republic	UTD	36	WOMAC Pain	WOMAC Function; Lequesne Index
Pham 2004 (29)	Observational cohort	301 knees	97:204	France	UTD	12	VAS Pain	Lequesne Index
Podsiadlo 2014 (28)	Observational cohort	114 knees	49:65	Australia	UTD	72	WOMAC Pain	WOMAC Function
Rat 2011 (82)	RCT	300 knees	118:182	France	67	6	SF-36 Body Pain; OAKHQOL Pain; VAS Pain	Lequense Index; SF-36 Physical; OAKHQOL Physical Activity

For peer review only - http://bmjopen.bmj.com/site/about/guidelines.xhtml

Raynauld 2011 (27)	RCT	123 knees	44:79	Canada	UTD	24	WOMAC Pain	WOMAC Function
Reginster 2001 (26)	RCT	212 knees	50:162	Belgium	UTD	36	WOMAC Pain	WOMAC Function
Reginster 2013 (83)	RCT	1371 knees	425:946	Australia Austria Belgium Canada Czech Republic Denmark Estonia France Germany Italy Lithuania Netherlands Poland Portugal Romania Russian Federation Spain United Kingdom	62.9	36	WOMAC Pain; VAS Pain	WOMAC Function
Riddle 2015 (25)	Observational cohort	467 knees	209:258	USA	UTD	24	KOOS Pain	WOMAC Function
Romagnoli 2017 (84)	Observational cohort	105 knees	16:69	Italy	67.7	66	Knee Society Score Clinical; VAS Pain	Knee Society Score Function; ROM
Roman-Blas 2017 (24)	RCT	158 knees	26:132	Spain	UTD	6	WOMAC Pain; VAS Pain	WOMAC Function
Rozendaal 2008 (31)	RCT	222 hips	68:154	Netherlands	UTD	24	WOMAC Pain; VAS Pain	WOMAC Function
Sanchez-Ramirez 2015 (85)	Observational cohort	186 knees	59:127	Canada	61	24	WOAMC Pain	WOMAC Function; Physical Test
Sawitzke 2010 (86)	RCT	662 knees	215:447	USA	57	24	WOMAC Pain	WOMAC Function
Skou 2016 (87)	Observational cohort	1682 knees	434:818	Denmark	62.2	84	WOMAC Pain	PASE; Physical Test
Sowers 2011 (88)	Observational cohort	724 knees	0:363	USA	56	132	NA	WOMAC Function; Physical Test
Spector 2005 (89)	RCT	284 knees	115:169	UK	63.3	12	WOMAC Pain	WOMAC Function

For peer review only - http://bmjopen.bmj.com/site/about/guidelines.xhtml

For peer review only

Sun 2017 (90)	RCT	121 knees	31:90	Taiwan	63	6	WOMAC Pain; VAS Pain	WOMAC Function; Lequesne Index; Physical Test
Urish 2013 (22)	RCT	336 knees	96:67	USA	UTD	36	WOMAC	WOMAC
Valdes 2012 (17)	Observational cohort	860 knees; 928 hips	UTD	UK	UTD	38	WOMAC Pain	NA
Van der Esch 2016 (98)	Observational cohort	402 knees	64:137	Netherlands	61.2	24	NRS Pain	WOMAC Function; Physical Test
Weng 2009 (91)	RCT	264 knees	26:106	Taiwan	64	12	VAS Pain	Lequesne Index; ROM; Physical Test
White 2016 (92)	Observational cohort	2110 knees	992:118	USA	61.0	84	VAS Pain	WOMAC Function
Witt 2005 (93)	RCT	294 knees	70:154	Germany	64.0	12	WOMAC Pain; SF-36 Body Pain; VAS Pain	WOMAC Function; SF-36 Function
Yu 2016 (21)	Observational cohort	204 knees	74:130	Australia	UTD	12	KOOS Pain; VAS Pain	KOOS ADL; Physical Function
Yusuf 2011 (94)	Observational cohort	74 knees; 31 hips; 11 hip and knees	19:98	Netherlands	60	72	WOMAC Pain; SF-36 Body Pain; Pain on movement	WOMAC Function; SF-36 Function; Physical Test

ADLs – Activities of Daily Living; IKDC - International Knee Documentation Committee; KOOS - Knee Injury and Osteoarthritis Outcome Score; LEFS – Lower Extremity Functional Scale; NA – not applicable; NRS – numerical rating scale; PASE – Physical Activity Scale for the Elderly; RCT – randomised controlled trial; ROM – range of motion; OAKHQOL - osteoarthritis knee and hip quality of life questionnaire; SF-36 – Short Form-36; UCLA Activity - UK – United Kingdom; USA - United States of America; UTD – unable to determine; VAS – visual analogue scale; WOMAC - Western Ontario and McMaster Universities Osteoarthritis Index

For peer review only

For peer review only - http://bmjopen.bmj.com/site/about/guidelines.xhtml

Table 2. Meta-Analysis Results: Exhibit Knee OA

Variable	N	Effect Estimate	P-Value	Statistical Heterogeneity (I^2%)	GRADE Assessment
Gender	823	0.91 (0.48 to 1.72)*	0.78	87	Low quality evidence[1]
Age	823	1.46 (0.26 to 2.66)	0.02	0	Moderate quality evidence[2]
KL ≥2	823	2.04 (1.48 to 2.81)	<0.01	35	Moderate quality evidence[2]
Knee effusion score ≥1	823	1.35 (0.99 to 1.83)	0.05	0	Moderate quality evidence[2]
BMI	823	-0.08 (-0.75 to 0.58)	0.81	0	Moderate quality evidence[2]

BMI – body mass index; KL – Kellgren Lawrence scale; I2 – inconsistency-squared; N- number of participants in analysis; NE – not estimable
* - random effects model analysis
[1]GRADE – Outcomes downgraded one level due to risk of bias, two level due to imprecision and inconsistency; [2]GRADE – Outcomes downgraded one level due to risk of bias

For peer review only - http://bmjopen.bmj.com/site/about/guidelines.xhtml

Figure and Table Legends

Figure 1: PRISMA flow-chart

Figure 2a: Forest-plot to present the association between gender and presentation of knee OA.
Figure 2b: Forest-plot to present the association between age and presentation of knee OA.
Figure 2c: Forest-plot to present the association between knee effusion score greater or equal to 1 and presentation of knee OA.
Figure 2d: Forest-plot to present the association between BMI and presentation of knee OA.

Table 1: Characteristics of included studies

Supplementary File 1: Search strategy adopted for the EMBASE database search.

Supplementary File 2: Methodological appraisal results based on the Downs and Black non-RCT Checklist

Supplementary File 3: Methodological appraisal results based on the Downs and Black RCT Checklist

For peer review only

For peer review only - http://bmjopen.bmj.com/site/about/guidelines.xhtml

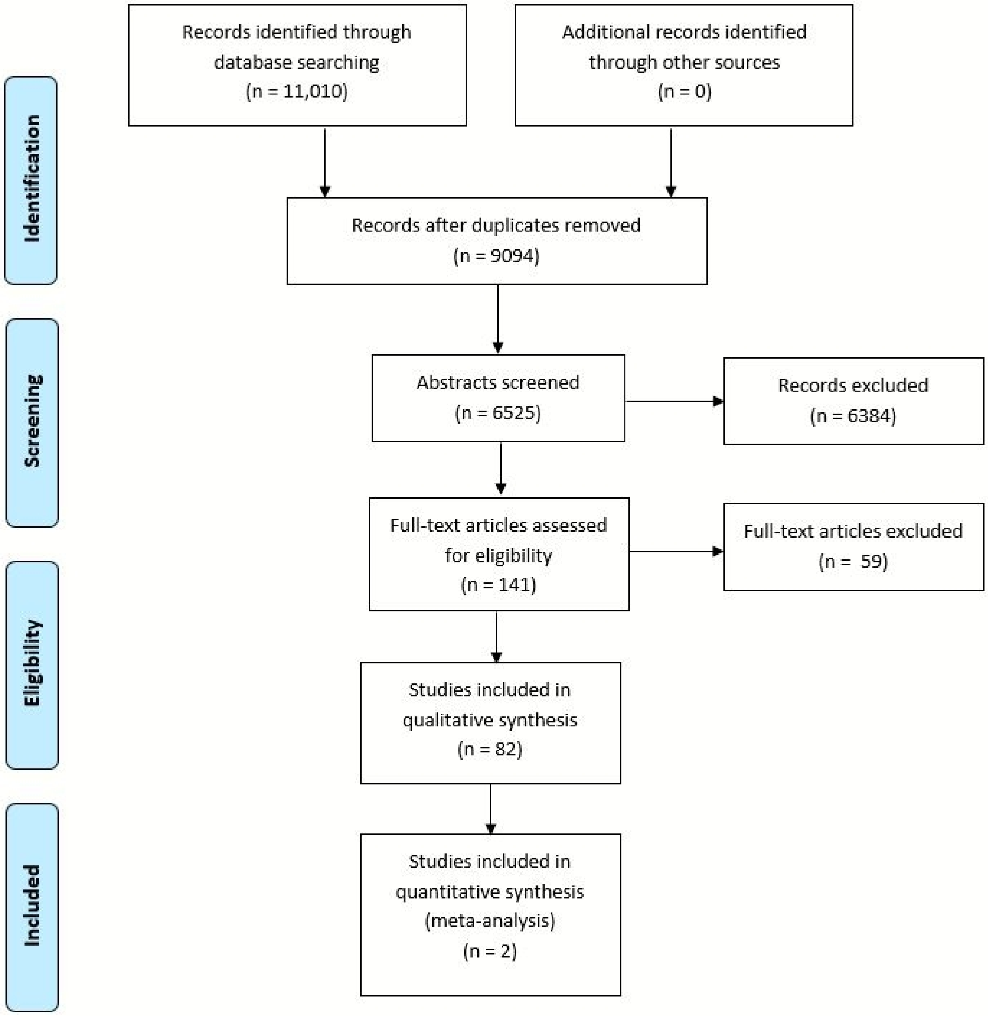

Figure 1

183x193mm (96 x 96 DPI)

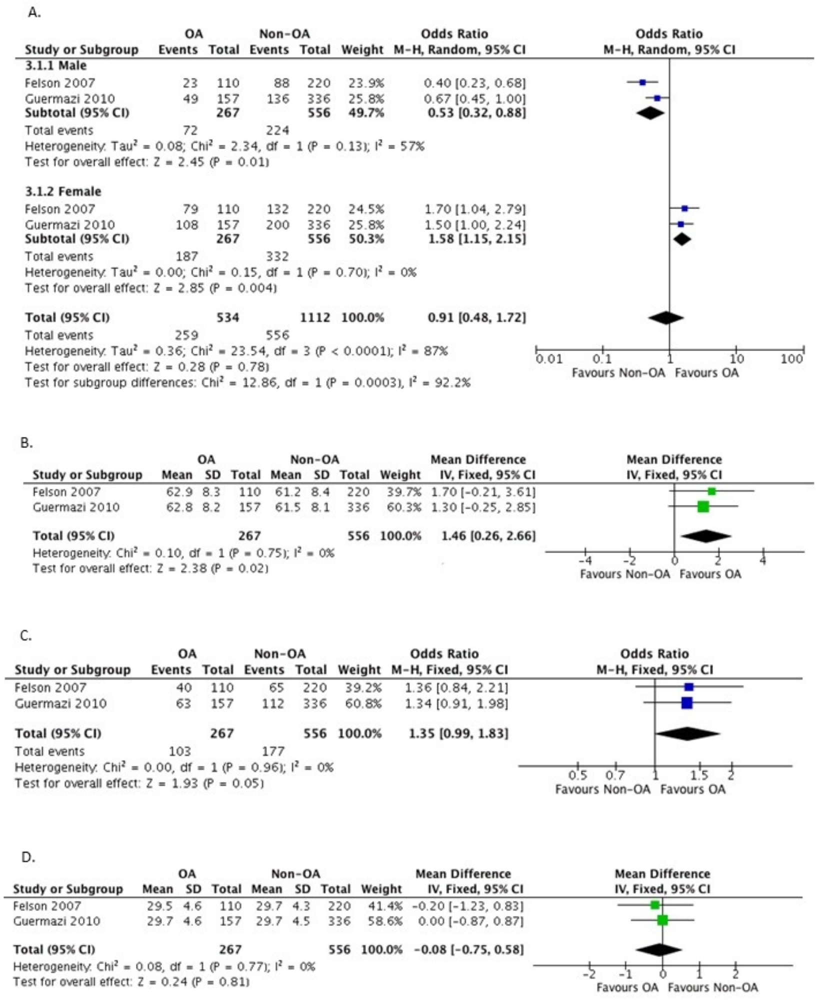

Figure 2a-2d

190x275mm (96 x 96 DPI)

Supplementary File 1: Methodological appraisal results based on the Downs and Black Observational Studies Checklist

	Downs and Black Observational Studies Checklist Items																		
	**1**	**2**	**3**	**4**	**5**	**6**	**7**	**8**	**9**	**10**	**11**	**12**	**13**	**14**	**15**	**16**	**17**	**18**	**Total**
Ahedi 2014	1	1	1	0	1	1	0	1	1	1	1	UTD	1	1	1	UTD	1	0	13
Amin 2008	1	1	0	1	1	1	1	0	0	UTD	1	1	1	1	UTD	1	1	0	12
Antony 2017	1	1	1	2	1	1	1	0	1	0	UTD	UTD	1	1	1	UTD	1	0	13
Baselga Garcia-Escudero 2015	1	1	1	0	1	1	1	1	UTD	UTD	1	1	1	1	UTD	0	1	1	13
Bevers 2015	1	1	1	2	0	1	1	1	UTD	0	1	1	1	1	1	1	1	0	15
Birmingham 2009	1	1	1	1	1	1	1	1	1	1	UTD	1	1	UTD	1	1	1	0	15
Chandrasekaran 2016A	1	1	1	1	1	1	1	1	0	UTD	1	1	1	1	UTD	1	1	1	15
Chandrasekaran 2016B	1	1	1	1	1	1	0	1	0	UTD	1	1	1	1	UTD	1	UTD	1	13
Davis 2017	1	1	1	0	0	1	1	0	1	1	1	UTD	1	1	1	UTD	1	0	12
Dowsey 2012	1	1	1	1	1	1	1	1	1	1	1	UTD	1	1	1	1	1	0	16
Eckstein 2013	1	1	1	2	1	1	1	1	1	1	1	1	1	UTD	1	1	1	0	17
Felson 2013	1	1	1	1	0	1	1	1	0	UTD	1	1	1	UTD	1	1	1	0	13
Filardo 2015	1	1	1	2	0	1	1	1	1	1	1	1	1	UTD	1	1	1	0	16
Glass 2013	1	1	1	2	0	1	1	1	1	1	1	1	1	1	1	1	1	0	17
Guermazin 2010	1	1	1	2	0	1	1	1	1	1	1	1	1	UTD	1	1	1	0	16
Hamilton 2017	1	1	0	0	1	1	1	1	UTD	UTD	1	1	1	1	UTD	UTD	1	1	12
Henriksen 2013	1	1	1	2	1	1	1	1	UTD	UTD	1	1	1	UTD	UTD	1	1	1	15
Huizinga 2017	1	1	1	0	1	1	1	0	UTD	UTD	1	1	1	1	UTD	0	1	0	11
Khan 2013	1	1	1	1	0	1	1	1	1	1	0	1	1	UTD	1	1	1	0	14
Kinds 2012	1	1	1	1	0	1	1	1	1	1	1	1	1	UTD	1	1	1	0	15
Messier 2005	1	1	0	2	1	1	0	1	1	UTD	1	1	1	UTD	1	1	1	0	14
Muraki 2014	1	1	1	1	1	1	1	1	1	UTD	1	1	1	1	0	1	1	0	15
Muraki 2015	1	1	1	2	1	1	1	1	1	UTD	1	1	1	1	0	1	1	0	16
Podsiadlo 2014	1	1	1	1	0	1	1	1	UTD	UTD	1	1	1	UTD	UTD	1	1	0	12
Rat 2011	1	1	1	1	1	1	1	1	UTD	UTD	1	1	1	1	0	UTD	1	1	14
Raynauld 2011	1	0	1	2	1	1	1	1	1	UTD	1	1	1	1	1	1	1	0	16
Riddle 2015	1	1	1	2	1	1	0	0	1	1	0	1	1	1	1	1	1	1	16
Romagnoli 2017	1	0	1	0	0	1	1	1	1	1	1	1	1	UTD	1	UTD	1	1	13

For peer review only - http://bmjopen.bmj.com/site/about/guidelines.xhtml

Sanchez-Ramirez 2015	1	1	1	2	1	1	1	1	1	1	1	1	1	UTD	1	1	1	0	17
Skou 2016	1	1	1	2	0	1	1	1	1	1	1	1	1	UTD	1	1	1	0	16
Sowers 2011	1	1	1	0	1	1	1	1	1	UTD	1	1	1	UTD	1	0	0	0	12
Urish 2013	1	1	1	1	1	1	1	1	1	1	1	1	1	1	1	0	1	0	16
Valder 2012	1	1	1	1	1	1	0	1	UTD	UTD	1	1	1	0	1	1	0	0	12
Van der Esch 2016	1	1	1	1	1	1	1	1	1	1	1	1	1	UTD	1	0	1	0	15
White 2016	1	1	1	2	0	1	1	0	1	1	1	1	1	1	0	1	1	0	15
Yu 2016	1	1	1	1	1	1	1	1	1	1	1	1	1	1	1	1	1	0	17
Yusuf 2011	1	1	1	1	1	1	1	0	UTD	UTD	1	1	1	UTD	1	1	1	0	13
Total with score >0	37	35	34	30	15	37	32	30	24	18	33	33	37	20	25	26	34	8	-

Checklist items

1. Is the hypothesis/aim/objective of the study clearly described?
2. Are the main outcomes to be measured clearly described in the Introduction or Methods section?
3. Are the characteristics of the patients included in the study clearly described?
4. Are the distributions of principal confounders in each group of subjects to be compared clearly described?
5. Are the main findings of the study clearly described?
6. Does the study provide estimates of the random variability in the data for the main outcomes?
7. Have the characteristics of patients lost to follow-up been described?
8. Have actual probability values been reported (e.g. 0.035 rather than <0.05) for the main outcomes except where the probability value is less than 0.001?
9. Were the subjects asked to participate in the study representative of the entire population from which they were recruited?
10. Were those subjects who were prepared to participate representative of the entire population from which they were recruited?
11. If any of the results of the study were based on “data dredging”, was this made clear?
12. Were the statistical tests used to assess the main outcomes appropriate?
13. Were the main outcome measures used accurate (valid and reliable)?
14. Were study participants in different groups (trials and cohort studies) or were the cases and controls (case-control studies) recruited over the same period of time?
15. Were the participants in different groups (trials and cohort studies) or were the cases and controls (case-control studies) recruited from the same population?
16. Was there adequate adjustment for confounding in the analyses from which the main findings were drawn?

For peer review only

For peer review only - http://bmjopen.bmj.com/site/about/guidelines.xhtml

17. Were losses of patients to follow-up taken into account?
18. Did the study mention having conducted a power analysis to determine the sample size needed to detect a significant difference in effect size for one or more outcome measures?

Footnote

UTD: Unable To Determine
2: Yes
1: Yes/partially
0: No

For peer review only

For peer review only - http://bmjopen.bmj.com/site/about/guidelines.xhtml

Supplementary File 2: Methodological appraisal results based on the Downs and Black non-RCT Checklist

	Downs and Black Non-Randomised Controlled Trial Checklist Items																		
	**1**	**2**	**3**	**4**	**5**	**6**	**7**	**8**	**9**	**10**	**11**	**12**	**13**	**14**	**15**	**16**	**17**	**18**	**Total**
Ahedi (54)	1	1	1	0	1	1	0	1	1	1	1	UC	1	1	1	UC	1	0	13
Amin (55)	1	1	0	1	1	1	1	0	0	UC	1	1	1	1	UC	1	1	0	12
Antony (560	1	1	1	2	1	1	1	0	1	0	UC	UC	1	1	1	UC	1	0	13
Baselga Garcia-Escudero (59)	1	1	1	0	1	1	1	1	UC	UC	1	1	1	1	UC	0	1	1	13
Bevers (60)	1	1	1	2	0	1	1	1	UC	0	1	1	1	1	1	1	1	0	15
Birmingham (61)	1	1	1	1	1	1	1	1	1	1	UC	1	1	UC	1	1	1	0	15
Chandrasekaran (48)	1	1	1	1	1	1	1	1	0	UC	1	1	1	1	UC	1	1	1	15
Chandrasekaran (47)	1	1	1	1	1	1	0	1	0	UC	1	1	1	1	UC	1	UC	1	13
Davis (19)	1	1	1	0	0	1	1	0	1	1	1	UC	1	1	1	UC	1	0	12
Dowsey (65)	1	1	1	1	1	1	1	1	1	1	1	UC	1	1	1	1	1	0	16
Felson (66)	1	1	1	1	0	1	1	1	0	UC	1	1	1	UC	1	1	1	0	13
Felson (67)	1	1	1	1	0	1	1	1	0	UC	1	1	1	UC	1	1	1	0	13
Glass (42)	1	1	1	2	0	1	1	1	1	1	1	1	1	1	1	1	1	0	17
Guermazin (41)	1	1	1	2	0	1	1	1	1	1	1	1	1	UC	1	1	1	0	16
Hamilton (68)	1	1	0	0	1	1	1	1	UC	UC	1	1	1	1	UC	UC	1	1	12
Huizinga (73)	1	1	1	0	1	1	1	0	UC	UC	1	1	1	1	UC	0	1	0	11
Khan (74)	1	1	1	1	0	1	1	1	1	1	0	1	1	UC	1	1	1	0	14
Muraki (79)	1	1	1	1	1	1	1	1	1	UC	1	1	1	1	0	1	1	0	15
Muraki (80)	1	1	1	2	1	1	1	1	1	UC	1	1	1	1	0	1	1	0	16
Pham (29)	1	1	1	1	1	1	1	1	UC	UC	1	1	1	0	1	1	1	0	14
Podsiadlo (28)	1	1	1	1	0	1	1	1	UC	UC	1	1	1	UC	UC	1	1	0	12
Riddle (25)	1	1	1	2	1	1	0	0	1	1	0	1	1	1	1	1	1	1	16
Romagnoli (84)	1	0	1	0	0	1	1	1	1	1	1	1	1	UC	1	UC	1	1	13
Sanchez-Ramirez (85)	1	1	1	2	1	1	1	1	1	1	1	1	1	UC	1	1	1	0	17
Skou (87)	1	1	1	2	0	1	1	1	1	1	1	1	1	UC	1	1	1	0	16
Sowers (88)	1	1	1	0	1	1	1	1	1	UC	1	1	1	UC	1	0	0	0	12
Valder (17)	1	1	1	1	1	1	0	1	UC	UC	1	1	1	0	1	1	0	0	12
Van der Esch (99)	1	1	1	1	1	1	1	1	1	1	1	1	1	UC	1	0	1	0	15
White (92)	1	1	1	2	0	1	1	0	1	1	1	1	1	1	0	1	1	0	15
Yu (21)	1	1	1	1	1	1	1	1	1	1	1	1	1	1	1	1	1	0	17
Yusuf (94)	1	1	1	1	1	1	1	0	UC	UC	1	1	1	UC	1	1	1	0	13

For peer review only

UC: Unclear; 2: Yes; 1: Yes/partially; 0: No

Checklist items

19. Is the hypothesis/aim/objective of the study clearly described?
20. Are the main outcomes to be measured clearly described in the Introduction or Methods section?
21. Are the characteristics of the patients included in the study clearly described?
22. Are the distributions of principal confounders in each group of subjects to be compared clearly described?
23. Are the main findings of the study clearly described?
24. Does the study provide estimates of the random variability in the data for the main outcomes?
25. Have the characteristics of patients lost to follow-up been described?
26. Have actual probability values been reported (e.g. 0.035 rather than <0.05) for the main outcomes except where the probability value is less than 0.001?
27. Were the subjects asked to participate in the study representative of the entire population from which they were recruited?
28. Were those subjects who were prepared to participate representative of the entire population from which they were recruited?
29. If any of the results of the study were based on "data dredging", was this made clear?
30. Were the statistical tests used to assess the main outcomes appropriate?
31. Were the main outcome measures used accurate (valid and reliable)?
32. Were study participants in different groups (trials and cohort studies) or were the cases and controls (case-control studies) recruited over the same period of time?
33. Were the participants in different groups (trials and cohort studies) or were the cases and controls (case-control studies) recruited from the same population?
34. Was there adequate adjustment for confounding in the analyses from which the main findings were drawn?
35. Were losses of patients to follow-up taken into account?
36. Did the study mention having conducted a power analysis to determine the sample size needed to detect a significant difference in effect size for one or more outcome measures?

For peer review only - http://bmjopen.bmj.com/site/about/guidelines.xhtml

Supplementary File 3: Methodological appraisal results based on the Downs and Black RCT Studies Checklist

	Downs and Black Randomised Controlled Trial Checklist Items																											
	**1**	**2**	**3**	**4**	**5**	**6**	**7**	**8**	**9**	**10**	**11**	**12**	**13**	**14**	**15**	**16**	**17**	**18**	**19**	**20**	**21**	**22**	**23**	**24**	**25**	**26**	**27**	**Total**
Akelman (20)	1	1	1	1	2	1	1	1	1	1	1	1	1	1	1	1	1	1	1	1	0	1	1	1	UC	1	1	**26**
Arden (57)	1	1	1	1	1	1	1	1	1	0	1	1	1	1	1	1	UC	1	1	1	1	1	1	1	0	1	UC	**23**
Ayral (58)	1	1	1	1	1	1	1	1	1	1	0	UC	UC	1	1	1	1	1	1	1	0	1	1	UC	UC	1	0	**20**
Bingham (53)	1	1	1	1	1	1	1	1	1	1	1	1	1	1	UC	1	1	1	1	1	0	1	0	UC	1	1	0	**22**
Bisicchia (52)	1	0	1	1	0	1	1	1	1	1	1	1	1	0	1	UC	1	1	1	1	1	1	1	0	0	1	0	**20**
Brandt (62)	1	1	1	1	1	0	1	1	1	1	UC	UC	UC	UC	1	1	1	1	1	1	UC	1	1	UC	0	1	0	**18**
Brown (50)	1	1	1	1	1	1	1	1	1	0	UC	UC	UC	1	1	1	1	1	1	1	UC	UC	1	UC	UC	UC	1	**18**
Brown (51)	1	1	1	1	1	1	1	1	1	0	UC	UC	UC	1	1	1	1	1	1	1	UC	UC	1	1	UC	1	1	**19**
Bruyere (63)	1	1	1	1	1	0	1	0	1	1	UC	UC	1	1	1	1	1	1	UC	1	UC	UC	1	UC	UC	1	1	**18**
Campbell (49)	1	1	1	1	0	0	0	1	1	0	1	UC	1	1	1	1	1	1	1	1	1	1	1	1	UC	1	0	**20**
Conrozier (64)	1	1	1	1	0	0	1	1	1	1	UC	UC	UC	1	1	1	1	0	1	1	0	1	1	1	0	1	UC	**18**
Dougados (46)	1	1	1	1	1	1	1	1	1	0	1	0	0	UC	UC	1	1	1	1	1	0	UC	1	UC	1	1	UC	**18**
Eckstein (45)	1	1	1	1	2	1	1	1	1	1	1	1	1	1	1	1	1	1	1	1	UC	1	1	1	1	1	0	**26**
Ettinger (44)	1	1	1	1	1	0	1	1	1	1	UC	UC	0	0	UC	1	1	1	UC	1	0	1	1	1	1	1	1	**19**
Filardo (43)	1	1	1	1	2	1	1	1	1	1	1	1	1	1	1	1	1	1	1	1	1	1	1	1	1	1	0	**25**
Hellio le Graverand (69)	1	1	1	1	1	0	1	1	1	1	UC	UC	UC	UC	1	1	UC	1	1	1	0	UC	1	1	UC	1	0	**17**
Henriksen (40)	1	1	1	1	2	1	1	1	0	1	UC	UC	1	0	1	1	1	1	1	1	1	1	1	1	1	1	1	**24**
Hill (5)	1	1	1	1	0	0	1	1	1	1	1	0	UC	1	1	1	1	1	1	1	0	1	1	1	0	1	1	**21**
Hochberg (70)	1	1	1	1	1	1	1	1	1	1	UC	UC	UC	1	UC	1	1	1	UC	1	0	UC	1	1	UC	1	0	**18**
Hoeksma (71)	1	1	1	1	0	1	1	1	1	0	1	1	0	0	1	1	1	1	0	1	1	1	1	1	UC	1	1	**21**
Housman (39)	1	1	1	1	0	0	1	1	1	0	0	UC	0	1	0	1	1	1	UC	1	0	1	1	UC	0	1	1	**16**
Huang (72)	1	1	1	1	0	1	1	0	1	0	UC	UC	UC	UC	1	1	UC	1	1	1	1	UC	1	UC	0	1	1	**16**
Jin (6)	1	1	1	1	0	1	1	1	0	1	UC	UC	0	1	1	1	1	1	UC	1	0	1	1	1	0	1	0	**18**
Karsdal (38)	1	1	1	1	1	0	1	1	1	0	UC	UC	UC	1	1	1	1	1	0	1	0	UC	1	1	1	1	UC	UC
Katz (37)	1	1	1	1	2	1	1	1	1	0	0	0	0	0	0	1	0	1	0	1	0	1	1	0	1	1	UC	**17**
Kim (75)	1	0	1	1	0	1	1	0	1	1	UC	UC	UC	1	1	1	0	1	1	1	UC	1	1	1	0	1	0	**17**

Kinds (18)	1	1	1	1	1	0	1	1	1	1	1	1	1	1	1	1	1	1	1	1	1	1	1	1	1	1	0	**25**
Kongtharvonskul (36)	1	1	1	1	2	1	1	1	0	1	1	UC	0	1	1	1	1	1	0	1	1	1	1	1	1	1	0	**23**
Lequesne (76)	1	1	1	1	1	1	1	1	1	1	UC	UC	0	1	1	1	1	1	1	1	0	UC	1	1	1	1	0	**21**
Lohmander (35)	1	1	1	1	0	1	1	1	1	1	UC	UC	0	1	1	0	1	1	1	1	0	1	1	1	0	1	1	**20**
Maheu (8)	1	1	1	1	0	1	0	1	0	1	UC	UC	0	1	1	1	1	UC	1	1	0	1	1	1	1	0	0	**17**
Marsh (34)	1	1	0	1	2	1	1	0	1	1	UC	UC	1	0	0	1	1	1	UC	1	UC	UC	UC	UC	1	1	0	**16**
McAlindion (33)	1	1	1	1	1	1	1	1	0	1	0	UC	0	1	1	1	1	1	1	1	0	1	1	1	UC	1	0	**20**
Messier (32)	1	1	1	1	1	0	1	1	0	0	0	UC	0	0	1	1	1	1	0	1	UC	1	1	1	1	0	1	**17**
Meissier (77)	1	1	0	1	2	1	1	1	0	1	1	1	1	1	1	1	1	1	1	1	1	1	1	1	1	1	0	**25**
Messier (78)	1	1	1	1	2	1	1	1	0	1	1	1	0	0	1	1	1	1	0	1	1	1	1	UC	1	1	1	**23**
Michel (31)	1	1	1	1	0	0	1	1	0	1	UC	UC	1	1	1	1	1	1	1	1	UC	1	1	1	0	0	1	**19**
Pavelka (30)	1	1	1	0	1	0	1	1	0	1	UC	UC	0	1	1	1	1	1	1	1	0	1	1	UC	1	1	0	**18**
Pavelka (81)	1	1	1	1	1	0	1	1	1	1	1	UC	1	1	1	1	1	1	1	1	1	1	1	1	1	1	1	**25**
Rat (82)	1	1	1	1	1	1	1	1	1	1	1	1	1	1	1	1	1	1	1	1	1	0	1	1	UC	1	1	**25**
Raynauld (27)	1	1	1	1	2	1	1	1	1	1	1	1	1	0	1	1	1	1	1	1	1	1	1	1	1	1	0	**26**
Reginster (83)	1	1	1	1	1	1	1	1	0	1	UC	UC	0	1	1	1	1	1	1	1	0	1	1	1	1	1	1	**22**
Reginster (26)	1	1	1	1	1	0	1	1	0	1	0	UC	1	1	1	1	1	1	1	1	1	1	1	1	UC	1	1	**22**
Roman-Blas (24)	1	1	1	1	1	0	1	1	1	1	UC	UC	0	1	1	1	1	1	0	1	0	UC	1	1	1	1	0	**19**
Rozendaal (31)	1	1	1	1	2	1	1	1	1	0	UC	UC	0	1	1	0	1	1	1	1	UC	1	1	1	1	1	UC	**21**
Sawitzke (86)	1	0	1	1	2	0	1	1	0	1	1	UC	0	1	1	1	1	1	1	1	UC	UC	1	UC	1	UC	UC	UC
Spector (89)	1	1	1	1	2	0	1	1	1	1	UC	UC	0	UC	UC	1	1	1	0	1	0	UC	1	UC	1	1	0	**17**
Sun (90)	1	1	1	1	1	1	1	1	1	1	UC	UC	1	1	1	1	1	1	1	1	UC	1	1	1	1	1	1	**24**
Urish (22)	1	1	1	1	1	1	1	1	1	1	1	1	1	0	1	1	1	1	1	1	1	1	1	1	0	1	0	**24**
Weng (91)	1	1	1	1	0	1	1	0	1	0	UC	UC	1	0	UC	1	1	1	1	1	UC	UC	1	1	0	1	1	**17**
Witt (93)	1	1	1	1	1	1	1	1	1	1	UC	UC	1	0	0	1	1	1	1	1	UC	1	1	1	1	1	1	**22**

UC: Unclear; 2: Yes; 1: Yes/partially; 0: No

Checklist items

1. Is the hypothesis/aim/objective of the study clearly described?
2. Are the main outcomes to be measured clearly described in the Introduction or Methods section?

For peer review only - http://bmjopen.bmj.com/site/about/guidelines.xhtml

3. Are the characteristics of the patients included in the study clearly described?
4. Are the interventions of interest clearly described?
5. Are the distributions of principal confounders in each group of subjects to be compared clearly described?
6. Are the main findings of the study clearly described?
7. Does the study provide estimates of the random variability in the data for the main outcomes?
8. Have all important adverse events that may be a consequence of the intervention been reported?
9. Have the characteristics of patients lost to follow-up been described?
10. Have actual probability values been reported (e.g. 0.035 rather than <0.05) for the main outcomes except where the probability value is less than 0.001?
11. Were the subjects asked to participate in the study representative of the entire population from which they were recruited?
12. Were those subjects who were prepared to participate representative of the entire population from which they were recruited?
13. Were the staff, places, and facilities where the patients were treated, representative of the treatment the majority of patients receive?
14. Was an attempt made to blind study subjects to the intervention they have received?
15. Was an attempt made to blind those measuring the main outcomes of the Intervention?
16. If any of the results of the study were based on “data dredging”, was this made clear?
17. In trials and cohort studies, do the analyses adjust for different lengths of follow-up of patients, or in case-control studies, is the time period between the intervention and outcome the same for cases and controls?
18. Were the statistical tests used to assess the main outcomes appropriate?
19. Was compliance with the intervention/s reliable?
20. Were the main outcome measures used accurate (valid and reliable)?
21. Were the patients in different intervention groups (trials and cohort studies) or were the cases and controls (case-control studies) recruited from the same population?
22. Were study subjects in different intervention groups (trials and cohort studies) or were the cases and controls (case-control studies) recruited over the same period of time?
23. Were study subjects randomized to intervention groups?
24. Was the randomized intervention assignment concealed from both patients and health care staff until recruitment was complete and irrevocable?
25. Was there adequate adjustment for confounding in the analyses from which the main findings were drawn?
26. Were losses of patients to follow-up taken into account?
27. Was there sufficient power to detect treatment effect at significance level of 0.05?

For peer review only

For peer review only - http://bmjopen.bmj.com/site/about/guidelines.xhtml

PRISMA 2009 Checklist

Section/topic	#	Checklist item	Reported on page #
**TITLE**			
Title	1	Identify the report as a systematic review, meta-analysis, or both.	Title
**ABSTRACT**			
Structured summary	2	Provide a structured summary including, as applicable: background; objectives; data sources; study eligibility criteria, participants, and interventions; study appraisal and synthesis methods; results; limitations; conclusions and implications of key findings; systematic review registration number.	Abstract page 2-3, registration, page 3, line 23-45
**INTRODUCTION**			
Rationale	3	Describe the rationale for the review in the context of what is already known.	Page 4, line 19-24, line 28-51
Objectives	4	Provide an explicit statement of questions being addressed with reference to participants, interventions, comparisons, outcomes, and study design (PICOS).	Page 5, line 5-17
**METHODS**			
Protocol and registration	5	Indicate if a review protocol exists, if and where it can be accessed (e.g., Web address), and, if available, provide registration information including registration number.	Page 5, line 25-29, line 32-44
Eligibility criteria	6	Specify study characteristics (e.g., PICOS, length of follow-up) and report characteristics (e.g., years considered, language, publication status) used as criteria for eligibility, giving rationale.	Page 5, line 48-58, page 6, line 1-20
Information sources	7	Describe all information sources (e.g., databases with dates of coverage, contact with study authors to identify additional studies) in the search and date last searched.	Page 5, line 32-45
Search	8	Present full electronic search strategy for at least one database, including any limits used, such that it could be repeated.	Supplementary File 1
Study selection	9	State the process for selecting studies (i.e., screening, eligibility, included in systematic review, and, if applicable, included in the meta-analysis).	Page 5, line 32-45, Page 6, line 24-31
Data collection process	10	Describe method of data extraction from reports (e.g., piloted forms, independently, in duplicate) and any processes for obtaining and confirming data from investigators.	Page 5, line 47-59, Page 6, line 1-31
Data items	11	List and define all variables for which data were sought (e.g., PICOS, funding sources) and any assumptions and simplifications made.	Page 5, line 32-58, page 6, line 1-31
Risk of bias in individual studies	12	Describe methods used for assessing risk of bias of individual studies (including specification of whether this was done at the study or outcome level), and how this information is to be used in any data synthesis.	Page 6, line 24-58, page 7, line 1-6

For peer review only

PRISMA 2009 Checklist

Summary measures	13	State the principal summary measures (e.g., risk ratio, difference in means).	Page 7, line 28-60
Synthesis of results	14	Describe the methods of handling data and combining results of studies, if done, including measures of consistency (e.g., I^2) for each meta-analysis.	Page 7, line 28-60, page 8, line 1-8

Section/topic	#	Checklist item	Reported on page #
Risk of bias across studies	15	Specify any assessment of risk of bias that may affect the cumulative evidence (e.g., publication bias, selective reporting within studies).	Page 6, line 35-60, page 7, line 1-6
Additional analyses	16	Describe methods of additional analyses (e.g., sensitivity or subgroup analyses, meta-regression), if done, indicating which were pre-specified.	Page 8, line 48-60, page 9, line 1-15
**RESULTS**			
Study selection	17	Give numbers of studies screened, assessed for eligibility, and included in the review, with reasons for exclusions at each stage, ideally with a flow diagram.	Page 8, line 37-45, Figure 1
Study characteristics	18	For each study, present characteristics for which data were extracted (e.g., study size, PICOS, follow-up period) and provide the citations.	Page 8, line 48-60, page 9, line 1-15
Risk of bias within studies	19	Present data on risk of bias of each study and, if available, any outcome level assessment (see item 12).	Page 9, line 18-60
Results of individual studies	20	For all outcomes considered (benefits or harms), present, for each study: (a) simple summary data for each intervention group (b) effect estimates and confidence intervals, ideally with a forest plot.	Figures 2a-d
Synthesis of results	21	Present results of each meta-analysis done, including confidence intervals and measures of consistency.	Results Table 2
Risk of bias across studies	22	Present results of any assessment of risk of bias across studies (see Item 15).	Supplementary File 2 and 3; Page 9, line 18-60
Additional analysis	23	Give results of additional analyses, if done (e.g., sensitivity or subgroup analyses, meta-regression [see Item 16]).	Page 10, line 34-60
**DISCUSSION**			
Summary of evidence	24	Summarize the main findings including the strength of evidence for each main outcome; consider their relevance to key groups (e.g., healthcare providers, users, and policy makers).	Page 11, line 38-60, page

For peer review only - http://bmjopen.bmj.com/site/about/guidelines.xhtml

PRISMA 2009 Checklist

			12, line 1-24
Limitations	25	Discuss limitations at study and outcome level (e.g., risk of bias), and at review-level (e.g., incomplete retrieval of identified research, reporting bias).	Page 12, line 27-56
Conclusions	26	Provide a general interpretation of the results in the context of other evidence, and implications for future research.	Page 11, line 36-60, page 13, line 31-51
**FUNDING**			
Funding	27	Describe sources of funding for the systematic review and other support (e.g., supply of data); role of funders for the systematic review.	Page 14, line 45-53

From: Moher D, Liberati A, Tetzlaff J, Altman DG, The PRISMA Group (2009). Preferred Reporting Items for Systematic Reviews and Meta-Analyses: The PRISMA Statement. PLoS Med 6(7): e1000097. doi:10.1371/journal.pmed1000097

For more information, visit: **www.prisma-statement.org**.

For peer review only

For peer review only - http://bmjopen.bmj.com/site/about/guidelines.xhtml
